# CCL7 and olfactory transduction pathway activation play an important role in the formation of CaOx and CaP kidney stones

**DOI:** 10.3389/fgene.2023.1267545

**Published:** 2024-01-03

**Authors:** Qiankun Zhang, Hhuiling Wei, Gang Huang, Lie Jin

**Affiliations:** ^1^ Division of Nephrology, The Fifth Affiliated Hospital of Wenzhou Medical University, Lishui Central Hospital, Lishui Hospital of Zhejiang University, Lishui, China; ^2^ Division of Traditional Chinese Medicine, The Fifth Affiliated Hospital of Wenzhou Medical University, Lishui Central Hospital, Lishui Hospital of Zhejiang University, Lishui, China

**Keywords:** kidney stone disease (KSD), CCL7, olfactory transduction pathway, hub gene, OR10A5

## Abstract

**Background:** The deposition of calcium oxalate (CaOx) and calcium phosphate (CaP) is the most common cause of kidney stone disease (KSD). Whether KSDs caused by CaOx and CaP have common genetic targets or signaling pathways remained unclear.

**Methods:** The present study utilized public data GSE73680 to analyze differentially expressed genes between CaOx or CaP tissues and normal tissues, respectively. Gene Ontology (GO) analysis and Kyoto Encyclopedia of Genes and Genomes (KEGG) pathway analysis of co-DEGs were performed. The protein-protein interaction (PPI) network was constructed to identify hub genes, and the top hub gene was selected for gene set enrichment analysis (GSEA). Finally, real-time PCR of patients’ urine was performed to validate the bioinformatic results.

**Results:** In total, 155 significantly co-upregulated DEGs and 64 co-downregulated DEGs were obtained from the datasets. The Gene Ontology analysis showed that DEGs were significantly enriched in chemical stimulus in sensory perception, detection of chemical stimulus in sensory perception of smell, and olfactory receptor activity. The KEGG analysis showed that the olfactory transduction pathway was significantly enriched. According to protein-protein interaction, 10 genes were identified as the hub genes, and CCL7 was the top hub gene. The olfactory transduction, maturity-onset diabetes of the young, linoleic acid metabolism, and fat digestion and absorption were significantly enriched in the high-CCL7 subgroup by GSEA. In total, 9 patients who had primarily CaOx mixed with some CaP stones and 9 healthy subjects were enrolled. The RT-PCR results showed that CCL7 level in the stone group was significantly higher than that in the control group (*p* < 0.05). For the olfactory transduction pathway, the expression of OR10A5, OR9A2, and OR1L3 was significantly upregulated in the stone group compared with the control group (*p* < 0.05).

**Conclusion:** CCL7 may play a key role in the development of both CaOx and CaP, and this process may depend on olfactory transduction pathway activation.

## 1 Introduction

Kidney stone disease (KSD) is a common disease leading to urinary tract obstruction and even renal failure, causing a substantial economic burden (Jeong et al., 2011). The prevalence of KSD is nearly 9% in the adult population and continues to increase worldwide (Sorokin et al., 2017; Scales et al., 2012). Calcium stones, particularly calcium oxalate (CaOx) or calcium phosphate (CaP) alone or in combination, are responsible for 85%–90% of all kidney stones (Khan et al., 2016; Leaf, 2010). They have common pathogenic factors, including supersaturation of calcium salts, low urine volume, and hypocitraturia (Worcester and Coe, 2010; Arcidiacono et al., 2014). However, there are some differences in physicochemical factors, metabolic factors, and histopathological factors underlying the formation of CaOx and CaP crystals (Daudon et al., 2016; Dessombz et al., 2015).

CaOx stones seem to develop on the kidney papillary surface attached to a CaP sub-epithelial plaque, known as Randal’s plaques (RP) (Evan et al., 2003). In contrast, patients with CaP stones often have early intratubular CaP deposits in their inner medullary collecting ducts, which have distinct microscopic features compared with RP (Evan et al., 2014). Moreover, CaOx or CaP as the main component may correspond to very different clinical implications (Parks et al., 2004). Thus seeking similar or different targets and signaling pathways provides a deeper insight into the molecular mechanisms of KSD.

## 2 Methods

### 2.1 Microarray data

Gene expression profiles of GSE73680 were downloaded from the Gene Expression Omnibus (GEO) database, and expression profiling arrays were generated using GPL17077. Biopsies were taken from human renal papillary tip tissues during endoscopic kidney stone surgery. In total, 24 samples were extracted in our study, including 13 CaOx plaque tissues, 5 CaP plaque tissues, and 6 normal control tissues. Then, the following steps were sequentially applied: removing the control probe, removing the probes that matched no gene symbol, removing the gene symbol with LOC or MT, and averaging duplicated gene probes. Finally, 28,892 genes were used for further analysis.

### 2.2 Identification of differentially expressed genes (DEGs)

To characterize DEGs, the limma package in the R software (version 4.2.2) was used to identify DEGs between the CaOx group and the control group with the threshold of *p*-value < 0.05 and |log2 fold change (FC)| >1. The limma package was also used to identify DEGs between the CaP group and the control group with the threshold of *p*-value < 0.05 and | FC| >1. The Venn diagram was constructed using the Venn diagram package. The volcano plot of DEGs was drawn by the ggplot2 package, and the heatmap of DEGs was generated using the pheatmap package in the R software.

### 2.3 Gene ontology and KEGG pathway analysis

To identify the potential functions and pathways between these DEGs, Gene Ontology (GO) and the Kyoto Encyclopedia of Genes and Genomes (KEGG) enrichment analyses were conducted using David, and results were visualized using R ggplot2 package. The GO terms of biological processes (BP), molecular functions (MF), and cellular components (CC) were respectively evaluated. The top ten terms were visualized if there were more than ten terms.

### 2.4 Construction of protein-protein interaction (PPI) network and identification of hub genes

To systematically analyze the biological functions of the obtained DEGs, the DEGs identified previously were mapped into the online search tool STRING database, which predicted the protein functional associations and PPI. Then, the Cytoscape software (version 3.7.2) was used for constructing and visualizing the transcriptional regulatory network of common DEGs. To identify hub genes, the sides of each node were estimated and the genes were sorted based on the rank of the connection number of each gene.

### 2.5 Gene set enrichment analysis (GSEA)

In our study, CCL7 was finally identified as the top hub gene for GSEA. Thirteen CaOx plaque tissues and 5 CaP plaque tissues were divided into high- and low-expression groups by the median expression value of CCL7. Then, GSEA analysis was applied to compare the differences in enhanced functions or pathways between high- and low-CCL7 expression groups using clusterprofiler package in the R software. Adjusted *p*-value < 0.05 was considered statistically significant.

### 2.6 Patients for experimental verification

In total, 9 patients with primarily CaOx mixed with CaP stones and 9 healthy subjects with similar gender and age were enrolled from April to October 2023. Patients’ kidney stone samples were collected after percutaneous nephrolithotomy, and stone composition was confirmed by the infrared spectrum analysis method. Patients with any evidence of kidney infection or dysfunction, a history of urinary tumors, or those younger than 18 years were excluded. On admission, midstream morning urine was collected from all participants before giving any medicine. This study was approved by the Human Ethics Committee of the Lishui Central Hospital (approved number 2023054). All patients signed the informed consent before participation.

### 2.7 Urine RNA isolation

Fresh urine was centrifuged 5000 rpm for 5 min, then discarded the supernatant. Total RNA was extracted from urine using RNAUrineKit (Solarbio, R1300-100T, China) according to the manufacturer’s instruction. The quality of all RNA samples was checked before downstream molecular biology applications. We used a tool for RNA quality assessment, called the RNA Integrity Number (RIN) which developed by Agilent Technologies. The RIN ranging from1 (degraded) to 10 (intact) was established by Agilent Bioanalyzer 2,100(Agilent Technologies). A RIN higher than five was used for downstream application. The concentration was assessed by NanoDrop 2000 (Thermo Fisher, United States). Extracted RNA samples with more than 50 ng/μL concentrations were used for analysis.

### 2.8 Real-time PCR analysis

The reverse transcription kit (Takara, RR037A, Japan) was applied to synthesize cDNA (CCL7, OR10A5, OR9A2, and OR1L3) according to the manufacturer’s protocol. RT-PCR was accomplished using HotStart™ 2X SYBR Green qPCR Master Mix (APExBIO, K1070, USA) to amplify these genes with primer pairs (Table 1). Total RNA of 1 μg was used for cDNA synthesis. All primers were synthesized by Sangon Biotech. formula 2^-△△Ct^ was used to analyze the relative changes in mRNA expression from RT-PCR (Pfaffl, 2001).

**TABLE 1:** List of primer pairs used in RT-PCR.

Primer names	Sequence (5’→3′)
CCL7-F	AGT​GTC​ACC​GAC​ATT​TAC​CTC​C
CCL7-R	AAG​GCG​GTA​GTG​AAT​TTG​CAC
OR10A5-F	CAT​TCT​TCA​CCT​CGC​TAT​TTC​TC
OR10A5-R	ATC​TTG​TGT​TCC​TAT​ACT​CGC​A
OR1L3-F	TTT​CCC​ATC​AAA​GTG​ACC​AG
OR1L3-R	TCC​AAC​CTG​ACA​AGA​CTC​TC
OR9A2-F	GAA​ATC​CAA​ACA​CCC​ATG​AC
OR9A2-R	CCA​TGT​ATT​TCT​TCC​TCA​GCC
GADPH-F	GGA​GCG​AGA​TCC​CTC​CAA​AAT
GADPH-R	GGC​TGT​TGT​CAT​ACT​TCT​CAT​GG

### 2.9 Statistical analysis

Statistical analysis was performed through R software (version 4.2.2) and GraphPad Prism software (version 9, San Diego, CA). Continuous normally distributed variables are presented in mean ± standard deviation (SD), and experimental results are expressed as mean ± standard error (SEM). Student’s t-test was applied to compare the stone and control group samples, with *p* < 0.05 showing a statistically significant difference. Receiver Operating Characteristic Curve (ROC) was constructed and used to evaluate the diagnostic value of CCL7 for patients with KSD.

## 3 Results

### 3.1 Identification of DEGs

**FIGURE 1:**
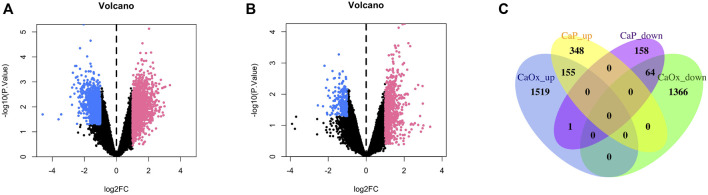
Volcano plot and Venn diagram of DEGs in the mRNA expression profiling datasets. **(A)** CaOx and normal renal papillary tip tissue; **(B)** CaP and normal renal papillary tip tissue; **(C)** Venn diagrams.

**FIGURE 2:**
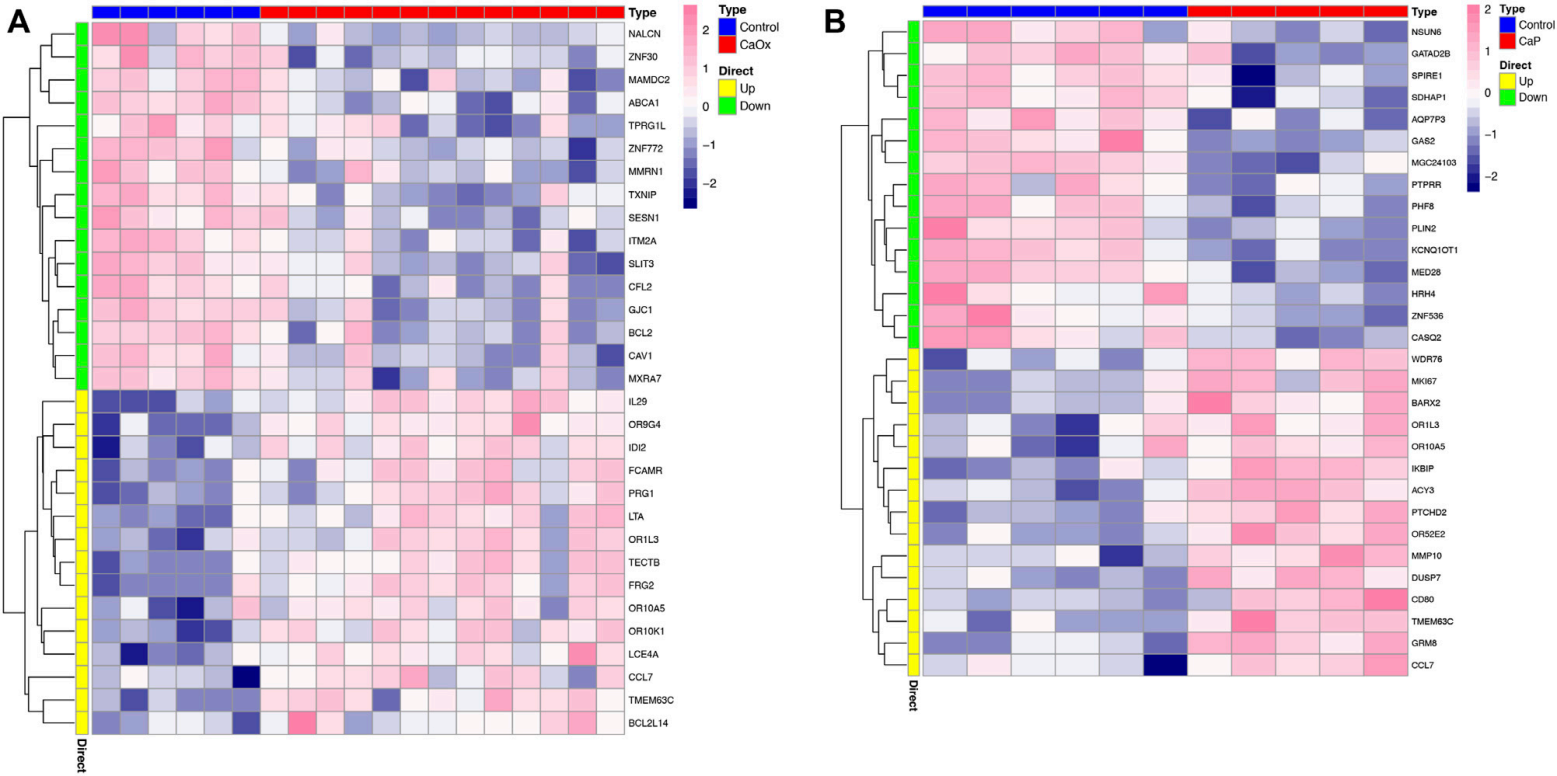
Heatmap of the top 30 DEGs. **(A)** CaOx and normal tissue; **(B)** CaP and normal tissue.

In total, 2,885 and 506 DEGs were identified from CaOx vs. control samples and CaP vs. control samples, respectively (see Supplementary Tables S1, S2). Volcano plots showed the distribution of these DEGs (Figures 1A, B). Subsequently, the intersection of these DEGs was identified using the Venn diagram, containing 155 significantly co-up-regulated genes and 64 co-down-regulated genes (Figure 1C and Supplementary Table S3). Therefore, the number of co-up-regulated DEGs was more than the number of co-down-regulated DEGs. The 30 most upregulated or downregulated DEGs were visualized in a heatmap (Figures 2A, B).

### 3.2 GO functional and KEGG pathway enrichment analyses of DEGs

**FIGURE 3:**
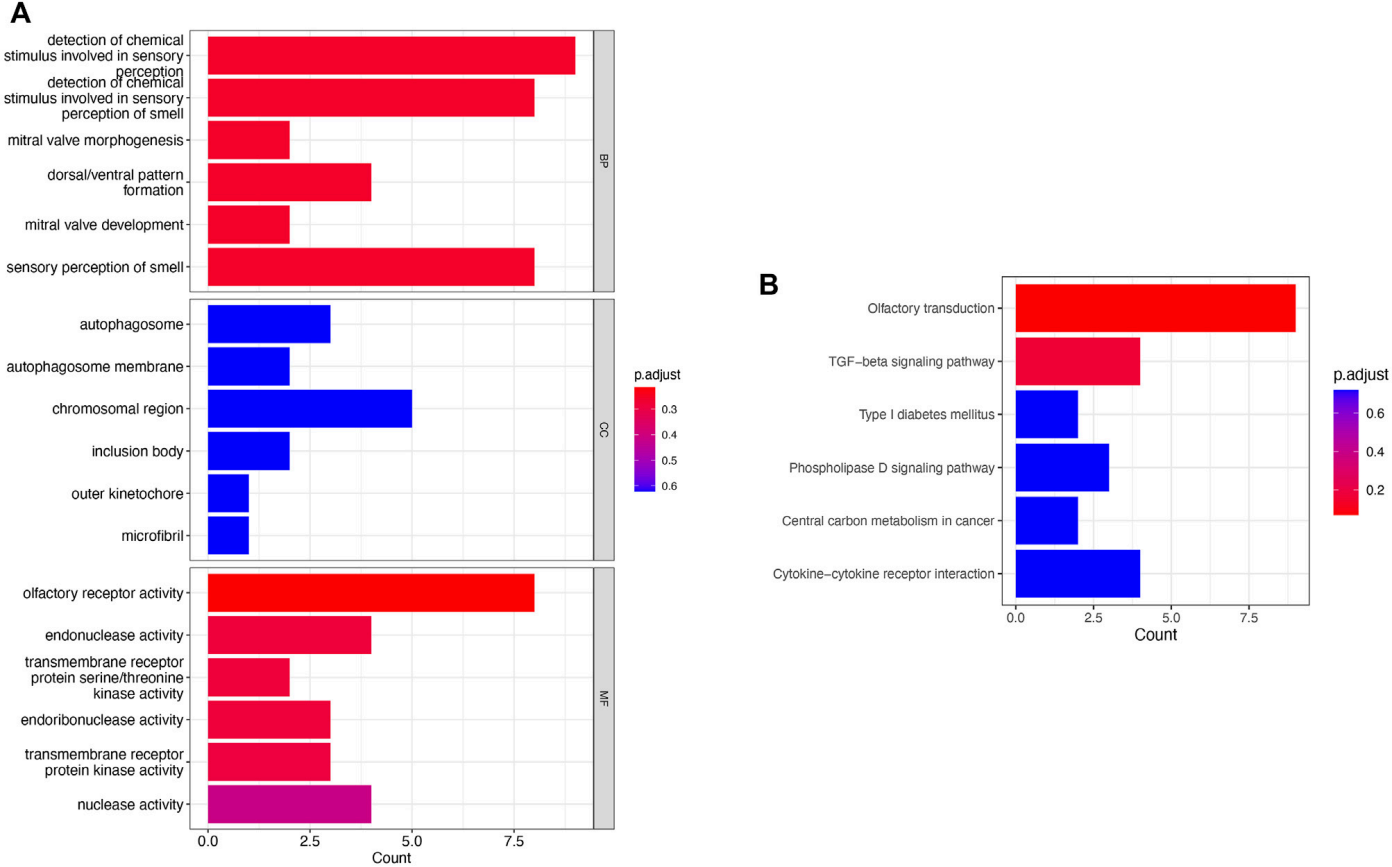
Pathway enrichment analysis. **(A)** GO enrichment analysis of the obtained 219 genes; **(B)** KEGG analysis of the obtained 219 genes.

To investigate the functions and mechanisms of these DEGs, GO and KEGG pathway enrichment analyses of upregulated and downregulated genes were performed. According to the results of enrichment analysis, 18 GO terms of DEGs (*p* < 0.05), including 6 BP, 6 CC, and 6 MF, were displayed (Figure 3A). The GO analysis showed that DEGs were significantly enriched in detection of chemical stimulus in sensory perception, detection of chemical stimulus in sensory perception of smell, and olfactory receptor activity (see Supplementary Table S4). Moreover, 6 KEGG pathways were over-represented in the DEGs, and the olfactory transduction pathway was significantly enriched. The results of the KEGG enrichment analysis are shown in Figure 3B and Supplementary Table S5.

### 3.3 Construction of PPI network and identification of hub genes

**FIGURE 4:**
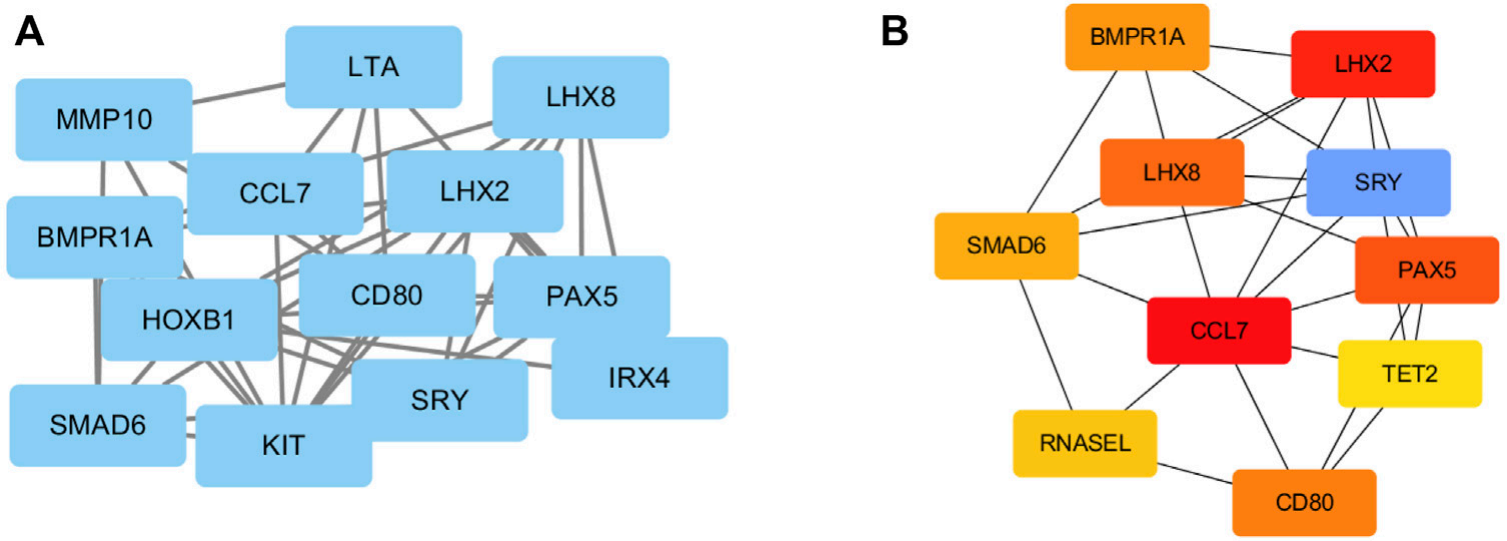
PPI network construction of DEGs. **(A)** The PPI network was drawn using Cytoscape, and the network nodes represent proteins. **(B)** The hub genes were obtained from the PPI network.

To investigate the relationships among these DEGs at the protein level, the PPI of the DEGs was obtained using STRING with a confidence score>0.4. Then, the PPI network of these DEGs was visualized using Cytoscape. Figure 4A presented a PPI network with 13 nodes and 27 edges, including 10 upregulated genes and 3 downregulated genes. In total, 10 genes were identified as hub genes according to the cytoHubba plugin of Cytoscape. They were all upregulated genes, and CCL7 was the top hub gene. The gene symbols and the detailed nodes and edges of the PPI network are shown in Figure 4B.

### 3.4 GSEA based on CCL7 expression

**FIGURE 5:**
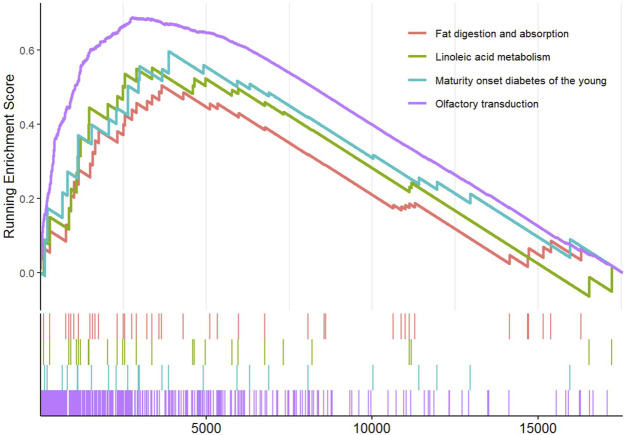
GSEA based on CCL7 expression levels.

Eighteen RP papillary tissues were divided into two subgroups based on the median expression of CCL7. The olfactory transduction, maturity-onset diabetes of the young, linoleic acid metabolism, and fat digestion and absorption were significantly enriched in the high-CCL7 subgroup (Figure 5).

### 3.5 Validation of identified key mRNAs in the stone group compared with the control group

**FIGURE 6:**
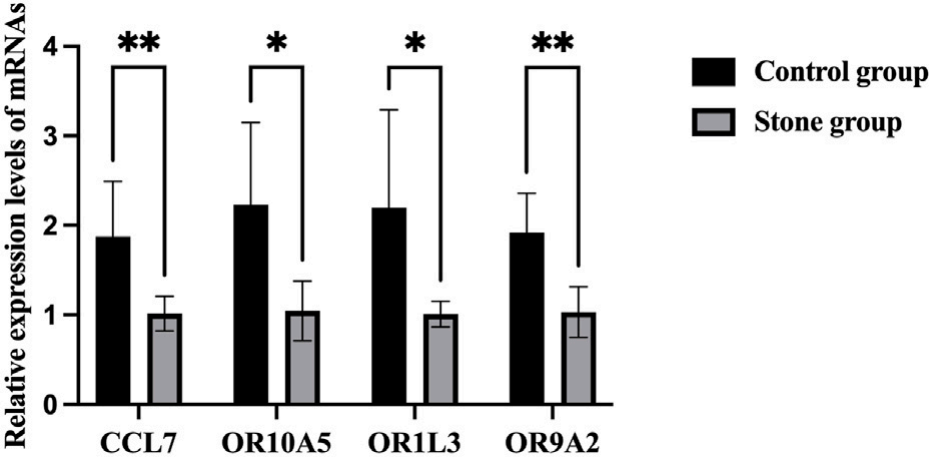
RT-PCR results of CCL7 and olfactory transduction pathway in cells of urine. *p < 0.05, **p < 0.01.

There was no significant difference in sex (both M/F 5:4) and age (52.8 ± 10.5 vs 54.2 ± 11.4 years) between the stone group and the control group (Table 1). The results of RT-PCR showed that CCL7 level in the stone group was significantly higher than that in the control group, which was consistent with the results of bioinformatic analysis. For the olfactory transduction pathway, the expression of OR10A5, OR9A2, and OR1L3 was significantly upregulated in the stone group compared with the control group (Figure 6), which was also consistent with bioinformatic results.

### 3.6 ROC curve for the diagnostic value of CCL7 in patients with KSD

**FIGURE 7:**
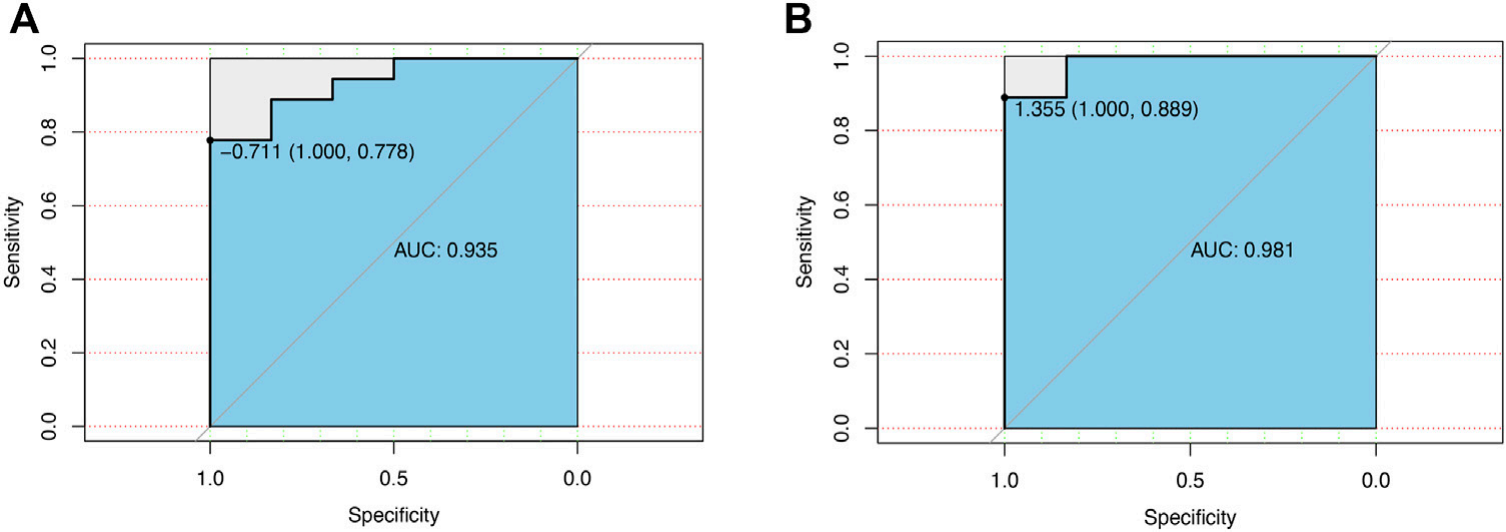
ROC curve for the diagnostic value of CCL7 in patients with KSD. **(A)** CCL7 in renal papillary tip tissues. **(B)** CCL7 in urine samples from our PCR results.

The value of CCL7 in both renal papillary tip tissues (24 patients) and urine samples from our PCR results (18 patients) in diagnosing KSD was investigated by plotting a ROC curve. As shown in Figures 7A, B, CCL7 in renal papillary tip tissues predicted the extent of KSD with AUC = 0.935 (*p* < 0.001), and CCL7 in urine samples predicted the extent of KSD with AUC = 0.985 (*p* < 0.001). They both had high sensitivity and a specificity.

## 4 Discussion

Most of the kidney stones are composed of calcium crystals, with CaOx as the most frequent composition, followed by CaP. Exploring the similarities and differences in molecular mechanisms responsible for the formation of various calcium crystals can improve the treatment of KSD. However, whether KSD caused by different crystals has similar molecular mechanisms remained unclear. Therefore, we analyzed the shared genes and signaling pathways responsible for the formation of two calcium crystals to explore common targets in kidney stones with different etiologies.

CCL7 gene plays a key biological function in both CaOx and CaP kidney stones, indicating that CCL7 may play a critical role in the development of calcium crystals. CCL7 (C-C Motif Chemokine Ligand 7) is a member of the C-C subfamily of chemokines, encoding monocyte chemotactic protein 3 (MCP3), a secreted chemokine which attracts macrophages during inflammation and metastasis. CCL7 can summon macrophages and monocytes, amplify inflammatory processes and contribute to disease progression in kidney diseases, cardiovascular diseases, and diabetes mellitus (Chang, 2022). Previous studies demonstrated the relationships between CCL7 and various kidney diseases, such as acute kidney injury, renal fibrosis, kidney stone, and end-stage renal disease (ESRD) (Chang, 2022; Zhou et al., 2020). Among 92 patients with kidney stone who underwent percutaneous nephrolithotomy, CCL7 expression was markedly enhanced in the papillary tips *versus* urine samples (Sun et al., 2018). In another study, treatment with oxalate upregulated CCL7 expression in human renal proximal tubular epithelial cells (Xia et al., 2021). Therefore, CCL7 may contribute to the development of both CaOx and CaP stones.

In our study, olfactory receptor activity and olfactory transduction pathway were significantly enriched in GO functional and KEGG pathway enrichment analyses. Using GSEA, we indicated that olfactory transduction was significantly enriched in the high-CCL7 subgroup. These findings indicated that the olfactory transduction pathway was significantly activated in patients with both CaOx and CaP stones. Olfactory receptors (ORs), belonging to a super-family of G-protein-coupled receptors, are chemo-sensors that sense smells in the nose (Massberg and Hatt, 2018). In addition to this specialized role in the olfactory epithelium, recent studies suggested that ORs are also found in non-sensory organs like kidneys (Rajkumar et al., 2014; Shepard and Pluznick, 2016). Few studies have specifically focused on their role in renal function. Pluznick et al. demonstrated that major components of the olfactory signaling pathway were present in the kidney and played important roles in regulating glomerular filtration rate (GFR) and renin release (Pluznick et al., 2009). Furthermore, Pluznick et al. determined that Olfr78 regulates blood pressure by interacting with byproducts of gut microbiota (Pluznick et al., 2013). Moreover, a recent study by Motahharynia et al. revealed that olfactory receptors contribute to the progression of kidney fibrosis (Motahharynia et al., 2022).

The above-mentioned findings demonstrated the role of these receptors in normal renal function, but no study focused on their function in the progression of KSD. We found 7 hub genes in this study, including CCL7, LTA, MMP3, IL9, CD80, MMP10, and IL31. They were all inflammatory factors. Considering the function of ORs in macrophage response and their potential role in inflammation (Vadevoo et al., 2021; Orecchioni et al., 2022), we speculated that the activated olfactory transduction pathway plays an important role in the progression of KSD. The results of RT-PCR indicated that the tendency of CCL7 and several genes of olfactory receptors (OR10A5, OR9A2, and OR1L3) were consistent with bioinformatic results.

In some cases drugs are necessary to reduce the risk of calcium stone formation, but no new drugs have been developed for calcium stone prevention in the past few decades. Our results suggested that olfactory receptor maybe a potential targets for calcium stones, and olfactory receptor inhibitors Phenirat^®^(Busse et al., 2014) and citral (Araneda et al., 2004) or silencing of olfactory receptor may provide a promising strategy to prevent and treat kidney calcium stones. However, as well as improving receptor pharmacology, work must also be done to confirm which receptors are medically viable targets.

However, our study has several limitations. First, there is a fundamental difference between urine and kidney. It is difficult to obtain kidney tissue of KSD patients since the progress of minimally invasive surgical technique, and kidney tissue of CaOx animal model or in cells may not really consistent with pathological alterations in clinic CaOx patients. In addition, to investigated the common pathogenic mechanism in KSD caused by CaOx and CaP, we chose patients with mixed CaOx and CaP, which can’t be achieved in animal model or in cells. Metabolite is highly correlated with CaOx kidney stone and the urine sample is one of the most direct and convenient body fluid for test. In a previous study, Liang et al. collected urine of urolithiasis patients and investigated lncRNA-miRNA-mRNA expression, they found most of these detected RNAs had been found to be associated with the stones formation (Liang et al., 2019). So mRNAs observed in urine may consistent with the natural pathogenesis of CaOx/CaP stone formation in kidney. Second, although we have enlarged the samples size, the size is still small, this may limit the statistical power and generalizability of the results. Third, a animal model or cell line validation to analyze detailed signalling pathway or detailed biological process is needed in the future.

## 5 Conclusion

In summary, our comprehensive bioinformatic analysis identified several molecular targets in KSD. Despite limitations, the major findings of this study are that CCL7 may play a key role in the development of both CaOx and CaP KSD, and this process relies on olfactory transduction pathway activation.

## Data Availability

The datasets presented in this study can be found in online repositories. The names of the repository/repositories and accession number(s) can be found in the article/Supplementary Material.
