# Supplementary material for: CCL7 and olfactory transduction pathway activation play an important role in the formation of CaOx and CaP kidney stones
Source: Front Genet. 2024 Jan 3;14:1267545. doi: 10.3389/fgene.2023.1267545 (PMC10791818; doi:10.3389/fgene.2023.1267545)
Supplement: Supplementary file 1 [file Table1.docx]

Table 1 DEGs of CaOx *vs* Control

| Gene.symbol | logFC | P.Value | adj.P.Val | Disease |
| --- | --- | --- | --- | --- |
| C14orf37 | -2.045836555 | 4.96696E-06 | 0.107232333 | Down |
| XLOC_l2_003753 | 2.028610132 | 7.42323E-06 | 0.107232333 | Up |
| C1orf198 | -1.610136885 | 2.23101E-05 | 0.20523476 | Down |
| OR9G4 | 1.621215607 | 2.84623E-05 | 0.20523476 | Up |
| NALCN | -2.268569162 | 4.49195E-05 | 0.20523476 | Down |
| XLOC_010492 | 2.158769334 | 6.67505E-05 | 0.20523476 | Up |
| OR10K1 | 1.487279792 | 6.93875E-05 | 0.20523476 | Up |
| XLOC_001499 | 1.839693246 | 7.02816E-05 | 0.20523476 | Up |
| ITM2A | -1.824895102 | 9.59202E-05 | 0.20523476 | Down |
| IDI2 | 1.573696098 | 0.000123999 | 0.20523476 | Up |
| XLOC_000699 | 1.561248264 | 0.000136716 | 0.20523476 | Up |
| TECTB | 1.850524043 | 0.000142832 | 0.20523476 | Up |
| TMEM63C | 1.709624347 | 0.000145063 | 0.20523476 | Up |
| XLOC_l2_009508 | 2.27400551 | 0.000169506 | 0.20523476 | Up |
| XLOC_007000 | 1.345681905 | 0.00018165 | 0.20523476 | Up |
| ZNF772 | -1.618684719 | 0.000186406 | 0.20523476 | Down |
| XLOC_010243 | 1.626597823 | 0.000199387 | 0.20523476 | Up |
| GJC1 | -2.498463551 | 0.000230836 | 0.20523476 | Down |
| MMRN1 | -2.573149815 | 0.000231432 | 0.20523476 | Down |
| CXorf36 | -1.573054208 | 0.000240257 | 0.20523476 | Down |
| FRG2 | 2.121345199 | 0.000246987 | 0.20523476 | Up |
| XLOC_004669 | 2.303414452 | 0.0002894 | 0.20523476 | Up |
| MAMDC2 | -2.270095602 | 0.000300135 | 0.20523476 | Down |
| IL29 | 1.213276441 | 0.000303857 | 0.20523476 | Up |
| XLOC_005945 | 1.550500326 | 0.00033038 | 0.20523476 | Up |
| FCAMR | 1.946483839 | 0.000368345 | 0.20523476 | Up |
| LCE4A | 1.461551659 | 0.000372518 | 0.20523476 | Up |
| SLIT3 | -1.731833979 | 0.000376972 | 0.20523476 | Down |
| TXNIP | -1.612489187 | 0.000379849 | 0.20523476 | Down |
| BCL2 | -1.907785764 | 0.000382105 | 0.20523476 | Down |
| SESN1 | -1.421197467 | 0.000408406 | 0.20523476 | Down |
| XLOC_009484 | 1.71412659 | 0.000429244 | 0.20523476 | Up |
| CFL2 | -2.715968247 | 0.000431344 | 0.20523476 | Down |
| PRG1 | 2.153540165 | 0.000449206 | 0.20523476 | Up |
| ZNF30 | -1.411503953 | 0.000463672 | 0.20523476 | Down |
| LTA | 2.018911509 | 0.000466039 | 0.20523476 | Up |
| XLOC_001767 | 1.393121047 | 0.000511384 | 0.20523476 | Up |
| XLOC_002530 | 1.571073567 | 0.00051265 | 0.20523476 | Up |
| XLOC_004374 | 1.284900106 | 0.00051565 | 0.20523476 | Up |
| XLOC_001912 | 2.397064541 | 0.000520954 | 0.20523476 | Up |
| ABCA1 | -1.097089303 | 0.000528671 | 0.20523476 | Down |
| RSPH4A | 1.047612214 | 0.000541121 | 0.20523476 | Up |
| CAV1 | -2.179235158 | 0.000543836 | 0.20523476 | Down |
| XLOC_l2_015221 | 1.709720052 | 0.000544344 | 0.20523476 | Up |
| XLOC_004211 | 1.482112875 | 0.000548405 | 0.20523476 | Up |
| XLOC_004074 | 1.448561126 | 0.000550156 | 0.20523476 | Up |
| XLOC_000564 | 2.211445145 | 0.000576929 | 0.20523476 | Up |
| MXRA7 | -1.914118134 | 0.000604801 | 0.20523476 | Down |
| FBN1 | -2.432475116 | 0.000615297 | 0.20523476 | Down |
| CYP3A4 | 1.214999478 | 0.000643659 | 0.20523476 | Up |
| XLOC_006421 | 1.626598872 | 0.00064651 | 0.20523476 | Up |
| OTP | 1.448640519 | 0.000650633 | 0.20523476 | Up |
| ASB15 | 1.865939981 | 0.00067447 | 0.20523476 | Up |
| SPARC | -2.52953048 | 0.000675008 | 0.20523476 | Down |
| XLOC_001497 | 1.491870684 | 0.00068301 | 0.20523476 | Up |
| XLOC_001768 | 1.646843149 | 0.000690388 | 0.20523476 | Up |
| ANKRD29 | -1.492135024 | 0.00070169 | 0.20523476 | Down |
| XLOC_014351 | 1.419321072 | 0.000702773 | 0.20523476 | Up |
| XLOC_012637 | 2.219762737 | 0.000712525 | 0.20523476 | Up |
| PRUNE | -1.433960106 | 0.000735581 | 0.20523476 | Down |
| XLOC_014122 | 1.261721702 | 0.000736366 | 0.20523476 | Up |
| XLOC_013268 | 1.868174765 | 0.000748215 | 0.20523476 | Up |
| CLLU1OS | 1.880442412 | 0.000751023 | 0.20523476 | Up |
| XLOC_010141 | 1.981248756 | 0.000779703 | 0.20523476 | Up |
| LNPEP | -1.535900153 | 0.000787931 | 0.20523476 | Down |
| XLOC_011706 | 2.086105986 | 0.000789135 | 0.20523476 | Up |
| XLOC_003574 | 1.406660623 | 0.000797748 | 0.20523476 | Up |
| LMOD2 | 1.599164924 | 0.000816603 | 0.20523476 | Up |
| XLOC_009363 | 2.762963839 | 0.000831785 | 0.20523476 | Up |
| OR51V1 | 1.560571341 | 0.000836436 | 0.20523476 | Up |
| PI15 | -2.389887387 | 0.000838953 | 0.20523476 | Down |
| XLOC_l2_001529 | -1.390724155 | 0.000842791 | 0.20523476 | Down |
| KDELC1 | -1.799564574 | 0.000843929 | 0.20523476 | Down |
| CCDC23 | -1.169536088 | 0.000865063 | 0.20523476 | Down |
| XLOC_l2_003475 | 1.763265428 | 0.000877049 | 0.20523476 | Up |
| ECM2 | -2.015794043 | 0.000883938 | 0.20523476 | Down |
| FLJ38773 | 1.201466329 | 0.000890878 | 0.20523476 | Up |
| GPX5 | 1.741173633 | 0.000918248 | 0.20523476 | Up |
| XLOC_010468 | 1.539575058 | 0.000927261 | 0.20523476 | Up |
| GFAP | 1.890751962 | 0.000933767 | 0.20523476 | Up |
| XLOC_013576 | 1.070722346 | 0.00094037 | 0.20523476 | Up |
| XLOC_006080 | 1.328987714 | 0.000943793 | 0.20523476 | Up |
| XLOC_006316 | 1.888289309 | 0.000947078 | 0.20523476 | Up |
| XLOC_008552 | 1.436531755 | 0.000951912 | 0.20523476 | Up |
| XLOC_005394 | 1.432000418 | 0.000956808 | 0.20523476 | Up |
| AMN1 | -1.233802038 | 0.000975623 | 0.20523476 | Down |
| SPIRE1 | -1.635848228 | 0.000984994 | 0.20523476 | Down |
| CST1 | 2.231673591 | 0.001015778 | 0.20523476 | Up |
| ELAVL2 | 1.916590069 | 0.001030669 | 0.20523476 | Up |
| RAB22A | -1.627109634 | 0.001033281 | 0.20523476 | Down |
| JPX | -1.045859286 | 0.001036752 | 0.20523476 | Down |
| XLOC_012196 | 1.462209119 | 0.001038409 | 0.20523476 | Up |
| XLOC_l2_007119 | 1.603978698 | 0.00103948 | 0.20523476 | Up |
| TTC29 | 1.398728227 | 0.001047758 | 0.20523476 | Up |
| TIMP2 | -1.821990396 | 0.001050665 | 0.20523476 | Down |
| XLOC_005024 | 1.57884191 | 0.001074601 | 0.20523476 | Up |
| FNBP1 | -1.826146476 | 0.001077461 | 0.20523476 | Down |
| XLOC_003223 | -1.837653018 | 0.00107917 | 0.20523476 | Down |
| LHFP | -2.370599936 | 0.001084488 | 0.20523476 | Down |
| XLOC_005912 | 1.194329002 | 0.001088028 | 0.20523476 | Up |
| MMP10 | 1.879383321 | 0.001092445 | 0.20523476 | Up |
| XLOC_005622 | 1.765774451 | 0.001102716 | 0.20523476 | Up |
| tAKR | 1.639619107 | 0.001115022 | 0.20523476 | Up |
| GAS6 | -1.431723307 | 0.001126301 | 0.20523476 | Down |
| XLOC_003522 | 2.432904115 | 0.001136081 | 0.20523476 | Up |
| XLOC_l2_002281 | 1.325721393 | 0.001141513 | 0.20523476 | Up |
| TREML1 | 2.473441638 | 0.001145034 | 0.20523476 | Up |
| XLOC_006320 | 1.19902442 | 0.00115347 | 0.20523476 | Up |
| KRTAP4-2 | 1.653440101 | 0.001156162 | 0.20523476 | Up |
| SIAH2 | -1.142794699 | 0.001156695 | 0.20523476 | Down |
| CTSK | -2.017192168 | 0.001159066 | 0.20523476 | Down |
| XLOC_005511 | 1.060433378 | 0.001163321 | 0.20523476 | Up |
| AHNAK2 | -1.683033996 | 0.001164664 | 0.20523476 | Down |
| LHX2 | 1.932191919 | 0.001166337 | 0.20523476 | Up |
| KIRREL3 | 1.437589714 | 0.001166964 | 0.20523476 | Up |
| POM121L9P | 1.365793133 | 0.00116774 | 0.20523476 | Up |
| XLOC_003477 | 1.561914935 | 0.001168494 | 0.20523476 | Up |
| XLOC_008401 | 2.236596082 | 0.001171904 | 0.20523476 | Up |
| XLOC_010235 | 1.791112925 | 0.001173206 | 0.20523476 | Up |
| RNF180 | -2.262406814 | 0.001181066 | 0.20523476 | Down |
| XLOC_006112 | 1.419799817 | 0.001190042 | 0.20523476 | Up |
| XLOC_007278 | 2.664422816 | 0.001199892 | 0.20523476 | Up |
| VENTXP7 | 1.305687372 | 0.001203737 | 0.20523476 | Up |
| PCP4 | -1.87462104 | 0.001228202 | 0.20523476 | Down |
| RTBDN | 2.014353492 | 0.001236351 | 0.20523476 | Up |
| ZDHHC22 | 1.808593186 | 0.001246865 | 0.20523476 | Up |
| XLOC_009628 | 1.02748305 | 0.001250798 | 0.20523476 | Up |
| XLOC_012525 | 1.590894931 | 0.001253094 | 0.20523476 | Up |
| XLOC_002961 | 1.341213994 | 0.001254798 | 0.20523476 | Up |
| XLOC_008510 | 2.210229541 | 0.001264855 | 0.20523476 | Up |
| XLOC_008693 | 1.813522482 | 0.001267098 | 0.20523476 | Up |
| FGF7 | -1.754439626 | 0.001268749 | 0.20523476 | Down |
| DNAJC27-AS1 | 1.236357363 | 0.001270823 | 0.20523476 | Up |
| XLOC_004004 | 1.614784972 | 0.001273416 | 0.20523476 | Up |
| COL3A1 | -2.646465823 | 0.001287309 | 0.20523476 | Down |
| XLOC_003275 | 1.366861666 | 0.001289901 | 0.20523476 | Up |
| EML1 | -1.409277091 | 0.001297951 | 0.20523476 | Down |
| XLOC_011616 | 1.33522416 | 0.001309832 | 0.20523476 | Up |
| B3GAT2 | -1.1694555 | 0.001310375 | 0.20523476 | Down |
| XLOC_009434 | 1.32836283 | 0.001318516 | 0.20523476 | Up |
| OR52E2 | 1.105336129 | 0.001325844 | 0.20523476 | Up |
| FAM75C2 | 1.273190429 | 0.001329065 | 0.20523476 | Up |
| XLOC_001667 | 2.310199194 | 0.001331518 | 0.20523476 | Up |
| XLOC_010321 | 1.66878528 | 0.001340163 | 0.20523476 | Up |
| GNAT3 | 1.177262587 | 0.001341191 | 0.20523476 | Up |
| LYSMD1 | -1.629179073 | 0.001342397 | 0.20523476 | Down |
| LRP6 | -1.420487849 | 0.00135348 | 0.20523476 | Down |
| GOLGA6A | 3.330626994 | 0.001354762 | 0.20523476 | Up |
| ZNF568 | -1.605272056 | 0.001357953 | 0.20523476 | Down |
| COLEC11 | -2.045902992 | 0.001364432 | 0.20523476 | Down |
| PPP1R14D | 1.446249059 | 0.001368491 | 0.20523476 | Up |
| ATP2B3 | 1.444131968 | 0.001381928 | 0.20523476 | Up |
| XLOC_004041 | 1.332734151 | 0.001397307 | 0.20523476 | Up |
| GATA6 | -1.99409499 | 0.001398932 | 0.20523476 | Down |
| ADAL | -1.443981516 | 0.001406001 | 0.20523476 | Down |
| C1orf21 | -1.363900745 | 0.001416808 | 0.20523476 | Down |
| TAB2 | -1.025732563 | 0.00141728 | 0.20523476 | Down |
| XLOC_l2_006548 | 1.667364455 | 0.001419669 | 0.20523476 | Up |
| XLOC_000149 | 1.882125818 | 0.001421847 | 0.20523476 | Up |
| RECK | -1.776434171 | 0.001435408 | 0.20523476 | Down |
| XLOC_010462 | 1.776124866 | 0.001438175 | 0.20523476 | Up |
| SMOC2 | -2.08882696 | 0.00145137 | 0.20523476 | Down |
| XLOC_002338 | 1.624474647 | 0.001452613 | 0.20523476 | Up |
| VCPIP1 | -1.006970255 | 0.00145565 | 0.20523476 | Down |
| OR10H5 | 1.836913942 | 0.001460599 | 0.20523476 | Up |
| XLOC_002390 | 1.24788568 | 0.001465618 | 0.20523476 | Up |
| C2orf27A | 2.473562608 | 0.001473448 | 0.20523476 | Up |
| TMEM191C | 1.278383066 | 0.001479576 | 0.20523476 | Up |
| DLX1 | 1.732598275 | 0.001506493 | 0.20523476 | Up |
| XLOC_003402 | 1.862442804 | 0.001510085 | 0.20523476 | Up |
| MPZ | 1.584481705 | 0.00152027 | 0.20523476 | Up |
| XLOC_l2_000773 | 1.400100597 | 0.001522506 | 0.20523476 | Up |
| XLOC_009544 | -1.31295841 | 0.001545627 | 0.20523476 | Down |
| OR2T6 | 1.686179364 | 0.001546908 | 0.20523476 | Up |
| OR11H12 | 3.040581286 | 0.001568258 | 0.20523476 | Up |
| XLOC_003961 | 1.390153267 | 0.001569473 | 0.20523476 | Up |
| XLOC_l2_002030 | 1.602034476 | 0.001569761 | 0.20523476 | Up |
| XLOC_008388 | 1.51102613 | 0.001569947 | 0.20523476 | Up |
| OR7A17 | 2.267992185 | 0.001577512 | 0.20523476 | Up |
| VWF | -1.493947474 | 0.001600497 | 0.20523476 | Down |
| XLOC_000619 | 1.568760289 | 0.001600856 | 0.20523476 | Up |
| GPR119 | 1.363725727 | 0.001604386 | 0.20523476 | Up |
| C10orf71 | 2.270423343 | 0.001617613 | 0.20523476 | Up |
| XLOC_011287 | 1.287362363 | 0.001627863 | 0.20523476 | Up |
| XLOC_010315 | 1.497346843 | 0.001629861 | 0.20523476 | Up |
| CEP63 | -1.130491945 | 0.001638464 | 0.20523476 | Down |
| XLOC_012912 | 1.349632049 | 0.001644067 | 0.20523476 | Up |
| TMEM158 | -2.151543686 | 0.001649562 | 0.20523476 | Down |
| ZNF383 | -1.162388694 | 0.001666219 | 0.20523476 | Down |
| OR2T34 | 2.938886862 | 0.001670078 | 0.20523476 | Up |
| EFHA2 | -2.023104321 | 0.001671161 | 0.20523476 | Down |
| PDX1 | 2.051543369 | 0.001672861 | 0.20523476 | Up |
| KATNAL1 | -1.379156166 | 0.001680258 | 0.20523476 | Down |
| IFNA2 | 1.428710032 | 0.001685663 | 0.20523476 | Up |
| LPP | -1.444366982 | 0.001693988 | 0.20523476 | Down |
| GUCY1A2 | -2.758707803 | 0.001695416 | 0.20523476 | Down |
| LYST | -1.144715863 | 0.001695472 | 0.20523476 | Down |
| CDRT7 | 1.527992157 | 0.001696654 | 0.20523476 | Up |
| XLOC_004598 | -1.567942713 | 0.001708076 | 0.20523476 | Down |
| XLOC_l2_013883 | 1.238721942 | 0.001709996 | 0.20523476 | Up |
| LTK | 1.788826241 | 0.001728109 | 0.20523476 | Up |
| OGT | -1.429305649 | 0.00173222 | 0.20523476 | Down |
| XLOC_007585 | 1.082105805 | 0.001737908 | 0.20523476 | Up |
| C9orf30-TMEFF1 | -1.280681272 | 0.001752597 | 0.20523476 | Down |
| XLOC_009312 | 1.560288582 | 0.001780175 | 0.20523476 | Up |
| XLOC_001378 | 2.849743126 | 0.001784394 | 0.20523476 | Up |
| CYP2C19 | 2.177521674 | 0.001788949 | 0.20523476 | Up |
| COL1A2 | -2.492651511 | 0.001801687 | 0.20523476 | Down |
| MZT2B | -1.137824888 | 0.001815947 | 0.20523476 | Down |
| COMMD10 | -1.064613424 | 0.001819167 | 0.20523476 | Down |
| XLOC_013714 | 1.847507816 | 0.001820531 | 0.20523476 | Up |
| CDKN3 | 1.387987555 | 0.001826114 | 0.20523476 | Up |
| VAV3 | -1.474259282 | 0.001826595 | 0.20523476 | Down |
| XLOC_000880 | 1.354346813 | 0.001831779 | 0.20523476 | Up |
| SMAD6 | -1.756331102 | 0.001832417 | 0.20523476 | Down |
| CD5L | 1.603580586 | 0.001835485 | 0.20523476 | Up |
| XLOC_l2_000920 | 1.842914888 | 0.001837298 | 0.20523476 | Up |
| SAMD5 | -1.379651073 | 0.001837822 | 0.20523476 | Down |
| DRD5 | 1.783133347 | 0.00184569 | 0.20523476 | Up |
| OR1L3 | 1.93655965 | 0.001846807 | 0.20523476 | Up |
| GOLGA7 | -1.181235567 | 0.001890187 | 0.20523476 | Down |
| XLOC_005571 | 1.21569925 | 0.001893751 | 0.20523476 | Up |
| GPR12 | 1.40022544 | 0.001904332 | 0.20523476 | Up |
| CALD1 | -2.228455894 | 0.001904405 | 0.20523476 | Down |
| LINC00478 | -1.656272341 | 0.001909237 | 0.20523476 | Down |
| GOLIM4 | -1.28642903 | 0.001918011 | 0.20523476 | Down |
| XLOC_008212 | 1.443245291 | 0.001920635 | 0.20523476 | Up |
| XLOC_012387 | 2.041041884 | 0.00193207 | 0.20523476 | Up |
| UVRAG | -1.232908848 | 0.001941212 | 0.20523476 | Down |
| FAM83C | 2.57576384 | 0.001945964 | 0.20523476 | Up |
| XLOC_005211 | 1.740668678 | 0.001946962 | 0.20523476 | Up |
| GLI3 | -1.799540501 | 0.00196422 | 0.20523476 | Down |
| C5orf22 | 1.113416189 | 0.001968424 | 0.20523476 | Up |
| XLOC_000017 | 1.956238199 | 0.001968709 | 0.20523476 | Up |
| RFTN1 | -1.164161998 | 0.001982267 | 0.20523476 | Down |
| POLI | -1.301262205 | 0.001991579 | 0.20523476 | Down |
| RNF17 | 1.35072532 | 0.001992165 | 0.20523476 | Up |
| RAI2 | -1.645024099 | 0.001992325 | 0.20523476 | Down |
| COL6A1 | -2.13335977 | 0.001994789 | 0.20523476 | Down |
| MRPS25 | -1.106276242 | 0.001996621 | 0.20523476 | Down |
| ULBP1 | 1.461120672 | 0.001997002 | 0.20523476 | Up |
| FLJ22763 | 1.496686382 | 0.002008583 | 0.20523476 | Up |
| KRT4 | 1.70561848 | 0.002018799 | 0.20523476 | Up |
| XLOC_008923 | 1.410191766 | 0.002023016 | 0.20523476 | Up |
| KIAA1530 | 1.289432249 | 0.002028629 | 0.20523476 | Up |
| XLOC_l2_014853 | 1.36262631 | 0.002031599 | 0.20523476 | Up |
| C20orf85 | 1.18265025 | 0.002036105 | 0.20523476 | Up |
| WNT1 | 2.201938358 | 0.002060529 | 0.20523476 | Up |
| C17orf6 | 1.432388069 | 0.002069688 | 0.20523476 | Up |
| COPZ2 | -2.148141143 | 0.002069938 | 0.20523476 | Down |
| SDHAP1 | -1.122893951 | 0.002084624 | 0.20523476 | Down |
| IL9 | 1.091574046 | 0.002088571 | 0.20523476 | Up |
| XLOC_006737 | 1.490897157 | 0.002096144 | 0.20523476 | Up |
| COL4A2 | -1.846750148 | 0.002114049 | 0.20523476 | Down |
| XLOC_009411 | 2.200839159 | 0.002125169 | 0.20523476 | Up |
| SLC25A12 | -1.197612338 | 0.002132076 | 0.20523476 | Down |
| C14orf2 | -1.137300126 | 0.002142166 | 0.20523476 | Down |
| HIRA | -1.365309914 | 0.002144958 | 0.20523476 | Down |
| MGC15885 | 1.316135452 | 0.002167396 | 0.20523476 | Up |
| NFIX | -1.513439509 | 0.002168668 | 0.20523476 | Down |
| ACYP1 | -1.578387505 | 0.002195059 | 0.20523476 | Down |
| XLOC_009713 | 1.402285223 | 0.002199406 | 0.20523476 | Up |
| GNPAT | -1.293997432 | 0.002210599 | 0.20523476 | Down |
| XLOC_001465 | 1.291241863 | 0.002210619 | 0.20523476 | Up |
| MC4R | 1.284147665 | 0.002212099 | 0.20523476 | Up |
| CCDC155 | 1.194324514 | 0.002218926 | 0.20523476 | Up |
| VWA3B | 1.229103439 | 0.002261875 | 0.20523476 | Up |
| XLOC_013737 | 1.202510021 | 0.002277574 | 0.20523476 | Up |
| FAM182A | 1.712603078 | 0.002284206 | 0.20523476 | Up |
| XLOC_005442 | 1.63697279 | 0.002294015 | 0.20523476 | Up |
| TMCC1 | -1.733671698 | 0.002294222 | 0.20523476 | Down |
| NOD2 | -1.642747051 | 0.002294481 | 0.20523476 | Down |
| BAG2 | -2.080019527 | 0.002314788 | 0.20523476 | Down |
| CORO1C | -1.224519544 | 0.002327909 | 0.20523476 | Down |
| ANKRD40 | -1.078068672 | 0.002335231 | 0.20523476 | Down |
| XLOC_014217 | 1.438754832 | 0.002336741 | 0.20523476 | Up |
| CAV2 | -1.752574234 | 0.002369109 | 0.20523476 | Down |
| FBXO16 | -1.451643276 | 0.002370792 | 0.20523476 | Down |
| XLOC_004119 | 1.164378174 | 0.002374047 | 0.20523476 | Up |
| C12orf43 | -1.145811601 | 0.002384523 | 0.20523476 | Down |
| XLOC_l2_009140 | 1.24216885 | 0.002385736 | 0.20523476 | Up |
| XLOC_l2_011227 | 2.205147322 | 0.002399522 | 0.20523476 | Up |
| ANGPT4 | 1.338120678 | 0.002400495 | 0.20523476 | Up |
| DCHS1 | -2.230769245 | 0.002405743 | 0.20523476 | Down |
| XLOC_006520 | 2.492002809 | 0.002408285 | 0.20523476 | Up |
| XLOC_l2_015288 | 1.349699547 | 0.002413624 | 0.20523476 | Up |
| XLOC_009767 | 2.448249291 | 0.002416889 | 0.20523476 | Up |
| CHRDL1 | -2.397817324 | 0.002421604 | 0.20523476 | Down |
| SDIM1 | 1.912607897 | 0.002434931 | 0.20523476 | Up |
| XLOC_003808 | 1.789398229 | 0.002435322 | 0.20523476 | Up |
| XLOC_001067 | 1.730268879 | 0.002440083 | 0.20523476 | Up |
| REG1A | 1.619845612 | 0.002452411 | 0.20523476 | Up |
| XLOC_000995 | 2.224324565 | 0.002471114 | 0.20523476 | Up |
| MYOZ2 | 1.019198048 | 0.002471122 | 0.20523476 | Up |
| WDR59 | -1.917093088 | 0.002480542 | 0.20523476 | Down |
| NMUR2 | 1.62029191 | 0.002489198 | 0.20523476 | Up |
| HTRA1 | -1.990562185 | 0.002527623 | 0.20523476 | Down |
| STX2 | -1.19715558 | 0.002530967 | 0.20523476 | Down |
| XLOC_010057 | 1.203514029 | 0.002536722 | 0.20523476 | Up |
| PAX5 | 1.465828232 | 0.002536841 | 0.20523476 | Up |
| EPN1 | -1.198741271 | 0.002558757 | 0.20523476 | Down |
| NDUFA12 | -1.047642969 | 0.002577254 | 0.20523476 | Down |
| DNAH10OS | 1.113380273 | 0.00259551 | 0.20523476 | Up |
| PRKAB2 | -1.314716461 | 0.002596674 | 0.20523476 | Down |
| XLOC_009534 | 1.445548913 | 0.002616247 | 0.20523476 | Up |
| XLOC_013049 | 1.423753956 | 0.002627941 | 0.20523476 | Up |
| NPVF | 1.04457605 | 0.002641308 | 0.20523476 | Up |
| THAP9 | -1.716219971 | 0.002642606 | 0.20523476 | Down |
| ZNF692 | -1.387636489 | 0.002650367 | 0.20523476 | Down |
| COL5A1 | -1.680898801 | 0.002651812 | 0.20523476 | Down |
| GNG12 | -1.512833073 | 0.002654514 | 0.20523476 | Down |
| SEC31B | -1.864307382 | 0.002676244 | 0.20523476 | Down |
| XLOC_003425 | 1.391756591 | 0.002677384 | 0.20523476 | Up |
| MAP4K4 | -1.642962683 | 0.0026882 | 0.20523476 | Down |
| FZD1 | -1.354335864 | 0.002690073 | 0.20523476 | Down |
| SP3 | -1.130775081 | 0.002697992 | 0.20523476 | Down |
| SERPINA12 | 1.099579728 | 0.002704215 | 0.20523476 | Up |
| WTH3DI | 1.618144352 | 0.002710787 | 0.20523476 | Up |
| NKX2-1-AS1 | 1.383801408 | 0.002739194 | 0.20523476 | Up |
| XLOC_002732 | 1.701023848 | 0.002741111 | 0.20523476 | Up |
| XLOC_001870 | 2.607795439 | 0.00274944 | 0.20523476 | Up |
| XLOC_005263 | 1.947982607 | 0.0027735 | 0.20523476 | Up |
| XLOC_l2_015143 | 1.270050412 | 0.002783343 | 0.20523476 | Up |
| ULK4 | -1.710131634 | 0.002786925 | 0.20523476 | Down |
| XLOC_009752 | 1.496448272 | 0.002791098 | 0.20523476 | Up |
| XLOC_007255 | 1.380158034 | 0.002794901 | 0.20523476 | Up |
| IL17RD | -2.070332844 | 0.002799441 | 0.20523476 | Down |
| COL5A2 | -2.168907466 | 0.002800542 | 0.20523476 | Down |
| XLOC_l2_015101 | 2.551108002 | 0.002806459 | 0.20523476 | Up |
| DACT3 | -1.590869645 | 0.002813534 | 0.20523476 | Down |
| XLOC_003897 | 1.319158716 | 0.002822669 | 0.20523476 | Up |
| XLOC_000618 | 1.561938591 | 0.002822915 | 0.20523476 | Up |
| PTPN21 | -1.779480106 | 0.002823322 | 0.20523476 | Down |
| XLOC_l2_004072 | 1.096180518 | 0.002862797 | 0.20523476 | Up |
| KIR2DL5A | 1.497187191 | 0.002873548 | 0.20523476 | Up |
| LIMD2 | -1.056561492 | 0.002882556 | 0.20523476 | Down |
| XLOC_l2_004706 | 1.402715674 | 0.002885563 | 0.20523476 | Up |
| MYH15 | 1.456075761 | 0.002887406 | 0.20523476 | Up |
| XLOC_000382 | 2.101371812 | 0.002887566 | 0.20523476 | Up |
| CAMK2G | -1.2755787 | 0.002888766 | 0.20523476 | Down |
| XLOC_011205 | 1.298952459 | 0.002893172 | 0.20523476 | Up |
| RASSF3 | -1.151639259 | 0.002894787 | 0.20523476 | Down |
| MKRN2 | -1.331986826 | 0.002906693 | 0.20523476 | Down |
| XLOC_004526 | 1.894792118 | 0.002942191 | 0.20523476 | Up |
| Q81CU5 | 2.265404985 | 0.002949243 | 0.20523476 | Up |
| JAZF1 | -1.592761289 | 0.002949661 | 0.20523476 | Down |
| TFAP2D | 1.114341149 | 0.002950716 | 0.20523476 | Up |
| FBLN5 | -2.616165339 | 0.002955335 | 0.20523476 | Down |
| ZPLD1 | 1.229330194 | 0.002973817 | 0.20523476 | Up |
| XLOC_011252 | 1.126275455 | 0.002975684 | 0.20523476 | Up |
| TWISTNB | -1.457902773 | 0.002977759 | 0.20523476 | Down |
| ZNF678 | -1.146374754 | 0.002988049 | 0.20523476 | Down |
| XLOC_012147 | 2.363478044 | 0.002997514 | 0.20523476 | Up |
| XLOC_007703 | 1.762551289 | 0.003004102 | 0.20523476 | Up |
| Q8SQ10 | -1.230774025 | 0.003015457 | 0.20523476 | Down |
| OGN | -1.926955574 | 0.003019219 | 0.20523476 | Down |
| XLOC_000367 | 2.171599835 | 0.003023477 | 0.20523476 | Up |
| XLOC_007643 | 1.909008927 | 0.003042115 | 0.20523476 | Up |
| DSTN | -1.270400878 | 0.003043754 | 0.20523476 | Down |
| SPRR2G | 1.373943886 | 0.003045845 | 0.20523476 | Up |
| XLOC_006658 | 1.307880042 | 0.003046081 | 0.20523476 | Up |
| SNORD115-1 | 1.859068196 | 0.003047881 | 0.20523476 | Up |
| XLOC_008769 | 1.261637894 | 0.003048263 | 0.20523476 | Up |
| KRTAP9-9 | 1.629342112 | 0.003063508 | 0.20523476 | Up |
| PPAPDC3 | 1.016525789 | 0.00306381 | 0.20523476 | Up |
| FAM197Y2P | 2.265002558 | 0.0030815 | 0.20523476 | Up |
| FAM208A | -1.478732544 | 0.00308524 | 0.20523476 | Down |
| XLOC_013612 | 1.136662154 | 0.003094754 | 0.20523476 | Up |
| TMPRSS6 | 1.774716988 | 0.003101415 | 0.20523476 | Up |
| CPNE2 | 1.809974139 | 0.00310239 | 0.20523476 | Up |
| ZC3H12B | -1.379699638 | 0.003102718 | 0.20523476 | Down |
| NAP1L1 | -1.388766333 | 0.003105089 | 0.20523476 | Down |
| XLOC_l2_010071 | 1.900831556 | 0.00310658 | 0.20523476 | Up |
| EPDR1 | -1.454983411 | 0.003106899 | 0.20523476 | Down |
| SGCE | -1.646384368 | 0.003108445 | 0.20523476 | Down |
| CRYM-AS1 | 1.607699148 | 0.003119006 | 0.20523476 | Up |
| HEY1 | -1.55232342 | 0.003129144 | 0.20523476 | Down |
| XLOC_007354 | 1.628998332 | 0.003133517 | 0.20523476 | Up |
| PTGFR | -1.527583851 | 0.003169765 | 0.20523476 | Down |
| TGFB3 | -2.145622856 | 0.00317364 | 0.20523476 | Down |
| XLOC_013770 | 2.128121226 | 0.003181084 | 0.20523476 | Up |
| DDX4 | 1.14726497 | 0.003193852 | 0.20523476 | Up |
| XLOC_l2_014955 | 1.012566856 | 0.003195016 | 0.20523476 | Up |
| MRGPRF | -1.904219241 | 0.003212553 | 0.20523476 | Down |
| NCS1 | -1.737239799 | 0.003220568 | 0.20523476 | Down |
| XLOC_l2_007707 | 1.058292789 | 0.003248324 | 0.20523476 | Up |
| HP1BP3 | -1.139782182 | 0.003284176 | 0.20523476 | Down |
| XLOC_000653 | 2.148719752 | 0.003284722 | 0.20523476 | Up |
| XLOC_009614 | 1.078222296 | 0.003290505 | 0.20523476 | Up |
| XLOC_008359 | 1.227022856 | 0.003312358 | 0.20523476 | Up |
| ARL2BP | -1.432092327 | 0.003319513 | 0.20523476 | Down |
| TMEM135 | -1.030156605 | 0.003364919 | 0.20523476 | Down |
| XLOC_006329 | 1.833309704 | 0.003365346 | 0.20523476 | Up |
| SNORD89 | -1.473336081 | 0.003368711 | 0.20523476 | Down |
| XLOC_l2_002207 | 2.311022552 | 0.003372381 | 0.20523476 | Up |
| UNC5CL | 1.019662771 | 0.003372514 | 0.20523476 | Up |
| XLOC_007234 | 1.716752523 | 0.00337871 | 0.20523476 | Up |
| FAM83A | 1.423600582 | 0.003391833 | 0.20523476 | Up |
| XLOC_010694 | 1.280956657 | 0.003399907 | 0.20523476 | Up |
| XLOC_007082 | 1.288954078 | 0.003404794 | 0.20523476 | Up |
| CCDC162P | 1.357008788 | 0.003415733 | 0.20523476 | Up |
| CREG1 | -1.287461767 | 0.003426821 | 0.20523476 | Down |
| STARD6 | 1.07623985 | 0.00342768 | 0.20523476 | Up |
| NDN | -1.484217495 | 0.003441754 | 0.20523476 | Down |
| OR10A2 | 1.053069541 | 0.003450248 | 0.20523476 | Up |
| OR51F1 | 1.837027093 | 0.003452682 | 0.20523476 | Up |
| XLOC_006361 | 1.191745351 | 0.003455708 | 0.20523476 | Up |
| XLOC_007037 | 1.338672998 | 0.003460716 | 0.20523476 | Up |
| XLOC_001734 | 1.408417514 | 0.003464245 | 0.20523476 | Up |
| RLBP1 | 1.649398627 | 0.003465789 | 0.20523476 | Up |
| RAB7A | -1.277811949 | 0.003472592 | 0.20523476 | Down |
| XLOC_007629 | 1.389081256 | 0.003476305 | 0.20523476 | Up |
| GATAD2B | -1.519466885 | 0.003482448 | 0.20523476 | Down |
| PDHB | -1.155144513 | 0.003485882 | 0.20523476 | Down |
| XLOC_011014 | 1.31923129 | 0.003486007 | 0.20523476 | Up |
| ARHGAP40 | 1.262797212 | 0.003503763 | 0.20523476 | Up |
| PALLD | -1.547927927 | 0.003513248 | 0.20523476 | Down |
| XLOC_011818 | 1.184573275 | 0.003513913 | 0.20523476 | Up |
| FAM55D | 1.486597641 | 0.003523696 | 0.20523476 | Up |
| TTTY13 | 2.336688979 | 0.003525833 | 0.20523476 | Up |
| C2orf53 | 1.239004633 | 0.003533756 | 0.20523476 | Up |
| TSPAN7 | -1.656091614 | 0.003536665 | 0.20523476 | Down |
| IRX4 | 1.446580633 | 0.003543856 | 0.20523476 | Up |
| XLOC_001853 | 1.597644648 | 0.003563205 | 0.20523476 | Up |
| XLOC_011106 | 1.757407472 | 0.003568809 | 0.20523476 | Up |
| RAB23 | -1.794406279 | 0.003569064 | 0.20523476 | Down |
| ZNF616 | -1.124230004 | 0.003575487 | 0.20523476 | Down |
| SMAD4 | -1.377214866 | 0.003576204 | 0.20523476 | Down |
| COL1A1 | -2.273519175 | 0.003585943 | 0.20523476 | Down |
| XLOC_012701 | 1.533157412 | 0.003588966 | 0.20523476 | Up |
| XLOC_001944 | 1.507166505 | 0.00359718 | 0.20523476 | Up |
| STAT5B | -1.177673386 | 0.003608021 | 0.20523476 | Down |
| XLOC_003367 | 1.201448012 | 0.003624173 | 0.20523476 | Up |
| XLOC_013812 | 1.438335287 | 0.003655394 | 0.20523476 | Up |
| P4HA1 | -1.546666639 | 0.003662196 | 0.20523476 | Down |
| XLOC_008222 | 1.323425667 | 0.003665909 | 0.20523476 | Up |
| RBL2 | -1.08963395 | 0.00367149 | 0.20523476 | Down |
| SERPINH1 | -1.354705532 | 0.00367171 | 0.20523476 | Down |
| MAST2 | -1.54756638 | 0.003678557 | 0.20523476 | Down |
| XLOC_001607 | 1.89498463 | 0.003687032 | 0.20523476 | Up |
| XLOC_008652 | 2.170420315 | 0.003691776 | 0.20523476 | Up |
| XLOC_005376 | 1.178472489 | 0.003693079 | 0.20523476 | Up |
| SIN3B | -1.26843812 | 0.003699133 | 0.20523476 | Down |
| XLOC_009943 | 1.311017613 | 0.003703096 | 0.20523476 | Up |
| TOMM7 | -1.271164435 | 0.003704776 | 0.20523476 | Down |
| SMARCAL1 | -1.139821555 | 0.003736127 | 0.20523476 | Down |
| FKSG83 | 1.51789868 | 0.003764673 | 0.20523476 | Up |
| XLOC_006903 | -1.330781864 | 0.003770575 | 0.20523476 | Down |
| SBK1 | -1.50557672 | 0.003790277 | 0.20523476 | Down |
| OXER1 | -1.148525513 | 0.00380328 | 0.20523476 | Down |
| XLOC_013285 | 1.833125892 | 0.003822423 | 0.20523476 | Up |
| C22orf40 | -1.64444092 | 0.003831703 | 0.20523476 | Down |
| DNASE1L3 | -1.701244834 | 0.003831801 | 0.20523476 | Down |
| XLOC_012811 | 1.833747827 | 0.003850808 | 0.20523476 | Up |
| PRKD3 | -1.140073684 | 0.003863817 | 0.20523476 | Down |
| XLOC_001654 | 1.149724677 | 0.003876637 | 0.20523476 | Up |
| ZPBP2 | 1.331995915 | 0.003906493 | 0.20523476 | Up |
| C8orf39 | -1.138654625 | 0.003908518 | 0.20523476 | Down |
| XLOC_013495 | 1.481557825 | 0.003911963 | 0.20523476 | Up |
| XLOC_008996 | 1.635739574 | 0.003915665 | 0.20523476 | Up |
| XLOC_l2_006575 | 1.335209075 | 0.003918109 | 0.20523476 | Up |
| XLOC_003863 | 1.276309008 | 0.003920491 | 0.20523476 | Up |
| XLOC_010411 | 1.078180121 | 0.003922674 | 0.20523476 | Up |
| MYL9 | -1.882927945 | 0.003925083 | 0.20523476 | Down |
| XLOC_010248 | 1.655214597 | 0.003931804 | 0.20523476 | Up |
| XLOC_013072 | 1.043597493 | 0.003940638 | 0.20523476 | Up |
| XLOC_005826 | 1.114482769 | 0.003940823 | 0.20523476 | Up |
| GRM8 | 1.164074808 | 0.003943157 | 0.20523476 | Up |
| XLOC_008450 | 1.678222888 | 0.003948519 | 0.20523476 | Up |
| XLOC_011970 | 1.600918812 | 0.003961388 | 0.20523476 | Up |
| ZC3H7B | -1.785537858 | 0.003969421 | 0.20523476 | Down |
| EFCAB7 | -1.466787142 | 0.003973762 | 0.20523476 | Down |
| CCDC80 | -2.870196485 | 0.003988143 | 0.20523476 | Down |
| ATP2B4 | -1.11242659 | 0.004001604 | 0.20523476 | Down |
| XLOC_004209 | 1.009491201 | 0.004007038 | 0.20523476 | Up |
| MIR143HG | -2.131911889 | 0.004010914 | 0.20523476 | Down |
| EMR4P | 1.663317666 | 0.004026247 | 0.20523476 | Up |
| XLOC_003093 | 2.38194566 | 0.004030062 | 0.20523476 | Up |
| VIP | 1.074174105 | 0.004032281 | 0.20523476 | Up |
| LIN7B | -1.355633734 | 0.004033679 | 0.20523476 | Down |
| RPL38 | -1.058355741 | 0.004036965 | 0.20523476 | Down |
| CTDSPL | -1.263340375 | 0.004038988 | 0.20523476 | Down |
| SIK3 | -1.225536601 | 0.004042182 | 0.20523476 | Down |
| FAM13C | -1.626801068 | 0.004050787 | 0.20523476 | Down |
| ZNF140 | -1.091009745 | 0.004059086 | 0.20523476 | Down |
| XLOC_005082 | 1.442581444 | 0.004074587 | 0.20523476 | Up |
| PSG8 | 1.229585178 | 0.004086138 | 0.20523476 | Up |
| C7orf52 | 1.519231043 | 0.004088959 | 0.20523476 | Up |
| XLOC_l2_004157 | 2.031862021 | 0.004098815 | 0.20523476 | Up |
| TEX101 | 1.070020727 | 0.004098839 | 0.20523476 | Up |
| KBTBD11 | -1.799982255 | 0.004103864 | 0.20523476 | Down |
| PTGFRN | -1.539084882 | 0.004123901 | 0.20523476 | Down |
| XLOC_l2_004944 | 1.887102153 | 0.004124867 | 0.20523476 | Up |
| EEA1 | -1.219680681 | 0.004125826 | 0.20523476 | Down |
| XLOC_001955 | 1.088006973 | 0.004130016 | 0.20523476 | Up |
| XLOC_006192 | 1.475336287 | 0.004139114 | 0.20523476 | Up |
| OR4X2 | 1.302431886 | 0.004170908 | 0.20523476 | Up |
| COX7A1 | -1.896854168 | 0.004185051 | 0.20523476 | Down |
| XLOC_007820 | 1.569301567 | 0.004192226 | 0.20523476 | Up |
| DNAJC5G | 1.773501721 | 0.004193001 | 0.20523476 | Up |
| UIMC1 | -1.088391332 | 0.004196989 | 0.20523476 | Down |
| XLOC_008479 | -1.198951628 | 0.004200511 | 0.20523476 | Down |
| LUM | -2.368529043 | 0.004202446 | 0.20523476 | Down |
| C7 | -2.249863646 | 0.004218938 | 0.20523476 | Down |
| XLOC_001787 | 1.310110899 | 0.004225589 | 0.20523476 | Up |
| XLOC_l2_009477 | 1.887199999 | 0.004227401 | 0.20523476 | Up |
| TSC1 | -1.201807458 | 0.004233768 | 0.20523476 | Down |
| CALHM3 | 1.405459755 | 0.004239324 | 0.20523476 | Up |
| XLOC_002433 | -1.234151503 | 0.004242171 | 0.20523476 | Down |
| ZNF20 | -1.479266732 | 0.004252705 | 0.20523476 | Down |
| GCM1 | 1.045852943 | 0.004265006 | 0.20523476 | Up |
| SH3GLB1 | -1.297010303 | 0.004266378 | 0.20523476 | Down |
| XLOC_006271 | 1.621222997 | 0.004277488 | 0.20523476 | Up |
| XLOC_010286 | 1.326611999 | 0.004304184 | 0.20523476 | Up |
| WFS1 | -1.104987859 | 0.004317843 | 0.20523476 | Down |
| FMO1 | 1.448333747 | 0.004330035 | 0.20523476 | Up |
| METTL16 | -1.226834834 | 0.004342955 | 0.20523476 | Down |
| FOXN3 | -1.145432818 | 0.004376774 | 0.20523476 | Down |
| TPM2 | -1.852015433 | 0.004389332 | 0.20523476 | Down |
| XLOC_013039 | 2.169262777 | 0.004393697 | 0.20523476 | Up |
| SNORA63 | -1.668851727 | 0.004395751 | 0.20523476 | Down |
| EHD2 | -1.393629559 | 0.004405038 | 0.20523476 | Down |
| MOV10L1 | 1.464766859 | 0.004409068 | 0.20523476 | Up |
| DENND2A | -1.320465548 | 0.004421207 | 0.20523476 | Down |
| GNPDA2 | -1.534565655 | 0.004424596 | 0.20523476 | Down |
| TTLL11 | -1.248507551 | 0.004426081 | 0.20523476 | Down |
| XLOC_l2_014171 | 1.195365968 | 0.004440353 | 0.20523476 | Up |
| XLOC_000495 | 1.458693135 | 0.004447832 | 0.20523476 | Up |
| XLOC_009089 | 1.363219837 | 0.004461066 | 0.20523476 | Up |
| FGF21 | 1.239292513 | 0.004464496 | 0.20523476 | Up |
| CRP | 1.928726745 | 0.00446531 | 0.20523476 | Up |
| XLOC_010360 | 1.49780479 | 0.004473079 | 0.20523476 | Up |
| ZCCHC24 | -1.748259235 | 0.004475013 | 0.20523476 | Down |
| CYP2C18 | 1.383612708 | 0.004497858 | 0.20523476 | Up |
| TTLL7 | -1.371820014 | 0.004512178 | 0.20523476 | Down |
| CRISPLD2 | -1.867170856 | 0.004514404 | 0.20523476 | Down |
| XLOC_009599 | 1.826831023 | 0.004528587 | 0.20523476 | Up |
| PSG6 | 1.753765689 | 0.004542515 | 0.20523476 | Up |
| XLOC_012379 | 1.50642799 | 0.004543099 | 0.20523476 | Up |
| TMEM61 | -1.247219451 | 0.004544696 | 0.20523476 | Down |
| NEMF | -1.015950307 | 0.004544705 | 0.20523476 | Down |
| SOS1-IT1 | -1.544255067 | 0.00454697 | 0.20523476 | Down |
| PALM | -1.490579661 | 0.004571372 | 0.20523476 | Down |
| CD109 | -1.457120734 | 0.004571828 | 0.20523476 | Down |
| KANK2 | -1.775302729 | 0.004574686 | 0.20523476 | Down |
| ATOH8 | -1.134256374 | 0.004582514 | 0.20523476 | Down |
| DPPA4 | 1.787460554 | 0.004604703 | 0.20523476 | Up |
| SRG7 | 1.165525665 | 0.004622856 | 0.20523476 | Up |
| ADAMTS1 | -1.562040419 | 0.004631847 | 0.20523476 | Down |
| SMARCB1 | -1.089743389 | 0.0046389 | 0.20523476 | Down |
| XLOC_010599 | 1.194094918 | 0.004640741 | 0.20523476 | Up |
| XLOC_007836 | 1.41961341 | 0.004656521 | 0.20523476 | Up |
| XLOC_006586 | 1.568363179 | 0.004662494 | 0.20523476 | Up |
| HOOK3 | -1.194727309 | 0.004663419 | 0.20523476 | Down |
| C3orf77 | 1.282175135 | 0.004666533 | 0.20523476 | Up |
| Q68PJ0 | 1.206553531 | 0.00466835 | 0.20523476 | Up |
| XLOC_009339 | 1.991679282 | 0.00467103 | 0.20523476 | Up |
| B3GNT1 | -1.51872934 | 0.004676077 | 0.20523476 | Down |
| FERMT2 | -1.316763916 | 0.004684097 | 0.20523476 | Down |
| RSBN1L | -1.007636182 | 0.004687713 | 0.20523476 | Down |
| OR4C45 | 2.072309913 | 0.004691486 | 0.20523476 | Up |
| NBPF22P | 1.572813777 | 0.004695493 | 0.20523476 | Up |
| BNIP3 | -1.283983046 | 0.004698408 | 0.20523476 | Down |
| XLOC_010165 | 1.206085034 | 0.004702215 | 0.20523476 | Up |
| XLOC_014251 | -1.065106472 | 0.004722278 | 0.20523476 | Down |
| SLC22A11 | 1.334640175 | 0.004737921 | 0.20523476 | Up |
| SNORD115-3 | 1.887995152 | 0.004742367 | 0.20523476 | Up |
| EPB41L4A-AS1 | -1.330009593 | 0.004761525 | 0.20523476 | Down |
| HSD3B1 | 1.205423751 | 0.004767807 | 0.20523476 | Up |
| POLK | -1.228887808 | 0.004769749 | 0.20523476 | Down |
| DEFB129 | 1.737950908 | 0.004772123 | 0.20523476 | Up |
| MFF | -1.184993957 | 0.004772802 | 0.20523476 | Down |
| POGK | -1.160502376 | 0.004784483 | 0.20523476 | Down |
| NR2F1 | -1.864490145 | 0.004786586 | 0.20523476 | Down |
| XLOC_003309 | 1.758346093 | 0.004789048 | 0.20523476 | Up |
| EDNRA | -1.644433312 | 0.00478978 | 0.20523476 | Down |
| NFE2L1 | -1.329855934 | 0.004806781 | 0.20523476 | Down |
| XLOC_011746 | 1.075114595 | 0.004808679 | 0.20523476 | Up |
| XLOC_007497 | 1.383923577 | 0.004810194 | 0.20523476 | Up |
| LCE2B | 1.17529276 | 0.004811957 | 0.20523476 | Up |
| XLOC_009964 | 1.273870163 | 0.004835933 | 0.20523476 | Up |
| XLOC_010430 | 1.303746951 | 0.004841556 | 0.20523476 | Up |
| XLOC_l2_015090 | 2.053386154 | 0.004846753 | 0.20523476 | Up |
| XLOC_l2_015265 | 1.098649902 | 0.004849715 | 0.20523476 | Up |
| KLK12 | 1.308303657 | 0.004889595 | 0.20523476 | Up |
| SMYD3 | -1.037795026 | 0.004890152 | 0.20523476 | Down |
| DENND5A | -1.097165984 | 0.004894806 | 0.20523476 | Down |
| FLJ12825 | 1.845819452 | 0.004903922 | 0.20523476 | Up |
| HIST1H2BJ | -1.398253391 | 0.004904485 | 0.20523476 | Down |
| XLOC_014014 | 1.258884774 | 0.004906441 | 0.20523476 | Up |
| GNB4 | -1.220566132 | 0.00490749 | 0.20523476 | Down |
| XLOC_011338 | 1.357864429 | 0.004912621 | 0.20523476 | Up |
| XLOC_002229 | 1.788654452 | 0.004937413 | 0.20523476 | Up |
| ABHD14A | -1.045940947 | 0.004938061 | 0.20523476 | Down |
| XLOC_012762 | 1.717021013 | 0.004960584 | 0.20523476 | Up |
| XLOC_007344 | 1.34316801 | 0.004966186 | 0.20523476 | Up |
| GAS5 | -1.334653667 | 0.004972514 | 0.20523476 | Down |
| XLOC_012724 | 1.519252366 | 0.004977056 | 0.20523476 | Up |
| XLOC_005881 | 1.279297046 | 0.004977165 | 0.20523476 | Up |
| XLOC_l2_009358 | 1.524423288 | 0.004979362 | 0.20523476 | Up |
| ALDH1B1 | -1.280886643 | 0.00498763 | 0.20523476 | Down |
| SSX5 | 1.021417975 | 0.004991225 | 0.20523476 | Up |
| ZFC3H1 | -1.331813632 | 0.004991731 | 0.20523476 | Down |
| SVEP1 | -1.864720148 | 0.004994509 | 0.20523476 | Down |
| ZNF521 | -1.285240566 | 0.005013056 | 0.20523476 | Down |
| XLOC_003103 | 1.140917995 | 0.005022482 | 0.20523476 | Up |
| C1orf49 | 1.112040341 | 0.005025519 | 0.20523476 | Up |
| XLOC_003044 | 1.551227808 | 0.005031189 | 0.20523476 | Up |
| KRTAP23-1 | 1.486346791 | 0.005045739 | 0.20523476 | Up |
| XLOC_000529 | 1.257826661 | 0.005046238 | 0.20523476 | Up |
| ARAP3 | -1.057883305 | 0.005046949 | 0.20523476 | Down |
| ATP8B4 | -1.652087234 | 0.005049524 | 0.20523476 | Down |
| XLOC_001866 | 2.200649581 | 0.005049849 | 0.20523476 | Up |
| SMARCD2 | -1.316060414 | 0.005054109 | 0.20523476 | Down |
| SRY | 1.508992272 | 0.005059239 | 0.20523476 | Up |
| C7orf41 | -1.267358697 | 0.005060869 | 0.20523476 | Down |
| TTTY18 | 1.515876758 | 0.005068597 | 0.20523476 | Up |
| RAB32 | -1.133192204 | 0.005070015 | 0.20523476 | Down |
| HERC3 | -1.029151825 | 0.005096197 | 0.20523476 | Down |
| FHL1 | -1.773708405 | 0.005096264 | 0.20523476 | Down |
| XLOC_000993 | 1.641629078 | 0.005111872 | 0.20523476 | Up |
| C12orf36 | 2.107927395 | 0.005114214 | 0.20523476 | Up |
| XLOC_011835 | 1.08813001 | 0.005119342 | 0.20523476 | Up |
| MPDZ | -1.191140921 | 0.005128016 | 0.20523476 | Down |
| XLOC_005572 | 1.423033003 | 0.0051474 | 0.20523476 | Up |
| KIAA0355 | -1.385956959 | 0.00515264 | 0.20523476 | Down |
| TCP11 | 1.081377237 | 0.005153055 | 0.20523476 | Up |
| XLOC_l2_002043 | 1.067838318 | 0.005161684 | 0.20523476 | Up |
| LEF1 | -1.920110331 | 0.005168529 | 0.20523476 | Down |
| METTL23 | -1.03197876 | 0.005189997 | 0.20523476 | Down |
| NEGR1 | 1.169449506 | 0.005210482 | 0.20523476 | Up |
| PPP1CB | -1.268361189 | 0.005217549 | 0.20523476 | Down |
| SOD3 | -1.934533145 | 0.005220264 | 0.20523476 | Down |
| CHURC1 | -1.871560118 | 0.005225118 | 0.20523476 | Down |
| DVL2 | -1.138677297 | 0.005228612 | 0.20523476 | Down |
| RCHY1 | -1.038643982 | 0.005268551 | 0.20523476 | Down |
| FAM138D | 1.47276842 | 0.0052724 | 0.20523476 | Up |
| DPYSL2 | -1.62320482 | 0.005290541 | 0.20523476 | Down |
| ACAD11 | -1.327406222 | 0.005310251 | 0.20523476 | Down |
| TM4SF20 | 1.311105707 | 0.005314377 | 0.20523476 | Up |
| ELP4 | -1.231692713 | 0.0053326 | 0.20523476 | Down |
| XLOC_001443 | 1.107026845 | 0.005337511 | 0.20523476 | Up |
| OR2L3 | 2.504358614 | 0.005341133 | 0.20523476 | Up |
| CTSS | -1.403578377 | 0.005342441 | 0.20523476 | Down |
| KRTAP9-8 | 1.416889213 | 0.005348974 | 0.20523476 | Up |
| ANKRD10-IT1 | -1.490151506 | 0.005356462 | 0.20523476 | Down |
| CRY1 | -1.277183001 | 0.005359933 | 0.20523476 | Down |
| RNF113A | -1.195919474 | 0.005362496 | 0.20523476 | Down |
| NDUFA1 | -1.112520993 | 0.005365939 | 0.20523476 | Down |
| ZNF804B | 1.785918998 | 0.005367696 | 0.20523476 | Up |
| LGALS14 | 1.641792286 | 0.005389885 | 0.20523476 | Up |
| MB | 1.367668374 | 0.005398896 | 0.20523476 | Up |
| KDELC2 | -1.534862922 | 0.005411914 | 0.20523476 | Down |
| ACCSL | 1.745582807 | 0.005460683 | 0.20523476 | Up |
| XLOC_013119 | 1.879535723 | 0.00546126 | 0.20523476 | Up |
| PRKG1 | -1.61072598 | 0.005466853 | 0.20523476 | Down |
| XLOC_005070 | 1.723795801 | 0.005467569 | 0.20523476 | Up |
| Q89B38 | 1.308279571 | 0.005477762 | 0.20523476 | Up |
| ISCA2 | -1.009940304 | 0.005485982 | 0.20523476 | Down |
| HIST1H2AC | -1.080658089 | 0.005486659 | 0.20523476 | Down |
| XLOC_009803 | 1.554456976 | 0.005509038 | 0.20523476 | Up |
| XLOC_012991 | 1.119387861 | 0.005531519 | 0.20523476 | Up |
| LHX9 | 2.066685464 | 0.005540359 | 0.20523476 | Up |
| C6orf35 | -1.540367908 | 0.00554068 | 0.20523476 | Down |
| XLOC_005141 | 1.193890364 | 0.005545469 | 0.20523476 | Up |
| MAP3K3 | -1.033306152 | 0.005553948 | 0.20523476 | Down |
| WTIP | -1.617967346 | 0.00556016 | 0.20523476 | Down |
| AEBP1 | -2.002646393 | 0.005575017 | 0.20523476 | Down |
| BMPR1A | -1.179256863 | 0.005591915 | 0.20523476 | Down |
| HOXC12 | 1.526713092 | 0.005603488 | 0.20523476 | Up |
| CSTT | 1.011046847 | 0.00560424 | 0.20523476 | Up |
| TCEA1 | -1.045327652 | 0.005621607 | 0.20523476 | Down |
| AMBN | 1.394672721 | 0.00562467 | 0.20523476 | Up |
| XLOC_009142 | 1.078546375 | 0.005628147 | 0.20523476 | Up |
| TRIML1 | 1.383507187 | 0.005629346 | 0.20523476 | Up |
| DEFB128 | 1.395109033 | 0.005633648 | 0.20523476 | Up |
| HILPDA | -1.383370175 | 0.005662632 | 0.20523476 | Down |
| PPP1R12A | -1.211575238 | 0.005666994 | 0.20523476 | Down |
| OR56A5 | 1.405825504 | 0.005674625 | 0.20523476 | Up |
| XLOC_005210 | 1.446847387 | 0.005679282 | 0.20523476 | Up |
| WDR38 | 1.255280206 | 0.005686646 | 0.20523476 | Up |
| MAP3K13 | -1.155290208 | 0.005694371 | 0.20523476 | Down |
| XLOC_000492 | 1.303086818 | 0.005706895 | 0.20523476 | Up |
| MBNL2 | -1.255743994 | 0.005707576 | 0.20523476 | Down |
| KLK14 | 1.552645756 | 0.005716456 | 0.20523476 | Up |
| XLOC_008697 | 1.230768409 | 0.0057237 | 0.20523476 | Up |
| XLOC_008968 | 2.189025304 | 0.005729571 | 0.20523476 | Up |
| XLOC_005337 | 1.188792623 | 0.005735181 | 0.20523476 | Up |
| AGPAT5 | -1.263138241 | 0.005737558 | 0.20523476 | Down |
| COMMD3 | -1.241403506 | 0.005745117 | 0.20523476 | Down |
| PLSCR4 | -1.441567811 | 0.005758004 | 0.20523476 | Down |
| XLOC_003771 | 1.332250116 | 0.005759221 | 0.20523476 | Up |
| SYT1 | 1.213934686 | 0.005777399 | 0.20523476 | Up |
| XLOC_006448 | 2.447428433 | 0.00577988 | 0.20523476 | Up |
| TNC | -1.769853662 | 0.005783091 | 0.20523476 | Down |
| XLOC_009833 | 1.716997699 | 0.005785726 | 0.20523476 | Up |
| DTWD1 | -1.581405599 | 0.005802298 | 0.20523476 | Down |
| RPL19P12 | -1.169910842 | 0.005842027 | 0.20523476 | Down |
| ZNRF2 | 1.182582531 | 0.005844957 | 0.20523476 | Up |
| XLOC_l2_000604 | 1.936789244 | 0.005860685 | 0.20523476 | Up |
| ITGA9 | -1.147193377 | 0.005869238 | 0.20523476 | Down |
| ZMYM6 | -1.197724585 | 0.005879477 | 0.20523476 | Down |
| XLOC_004072 | 1.390135223 | 0.005891695 | 0.20523476 | Up |
| XLOC_005557 | 1.577980986 | 0.00591782 | 0.20523476 | Up |
| ELAVL4 | 1.419777137 | 0.005927698 | 0.20523476 | Up |
| XLOC_004448 | 1.396379682 | 0.00593038 | 0.20523476 | Up |
| GK2 | 2.441302886 | 0.005966363 | 0.20523476 | Up |
| FZD2 | -1.337527636 | 0.005973553 | 0.20523476 | Down |
| PATE2 | 1.889796239 | 0.005985636 | 0.20523476 | Up |
| SKA3 | 2.642114974 | 0.005987084 | 0.20523476 | Up |
| ILK | -1.231994632 | 0.005989429 | 0.20523476 | Down |
| PTGR1 | 1.277362336 | 0.005991005 | 0.20523476 | Up |
| IRG1 | 1.203367484 | 0.005995733 | 0.20523476 | Up |
| ZDHHC19 | 1.2739367 | 0.005998942 | 0.20523476 | Up |
| XLOC_014347 | 1.060668301 | 0.006001199 | 0.20523476 | Up |
| XLOC_002698 | 1.773932715 | 0.006004166 | 0.20523476 | Up |
| CLIC4 | -1.641580916 | 0.006009181 | 0.20523476 | Down |
| XLOC_l2_002254 | 1.506432908 | 0.006016406 | 0.20523476 | Up |
| RHOBTB1 | -1.162514955 | 0.006016717 | 0.20523476 | Down |
| LUC7L3 | -1.236434302 | 0.006022354 | 0.20523476 | Down |
| XLOC_012256 | 1.110253131 | 0.006024957 | 0.20523476 | Up |
| XLOC_013744 | 1.002671549 | 0.006025475 | 0.20523476 | Up |
| XLOC_l2_010083 | 1.095773208 | 0.006026776 | 0.20523476 | Up |
| XLOC_000556 | 2.118110039 | 0.006034978 | 0.20523476 | Up |
| RBM17 | -1.172099853 | 0.006045311 | 0.20523476 | Down |
| XLOC_002953 | 1.13586469 | 0.006049552 | 0.20523476 | Up |
| GDI1 | -1.087645693 | 0.006049617 | 0.20523476 | Down |
| PXK | -1.168683599 | 0.006051026 | 0.20523476 | Down |
| XLOC_007753 | 1.756333971 | 0.006056719 | 0.20523476 | Up |
| XLOC_008341 | 2.049167218 | 0.006057522 | 0.20523476 | Up |
| XLOC_013811 | 1.918955121 | 0.00606422 | 0.20523476 | Up |
| NCRUPAR | 1.286264341 | 0.006070054 | 0.20523476 | Up |
| PRR24 | -1.450866924 | 0.006070127 | 0.20523476 | Down |
| FAM8A1 | -1.401303766 | 0.006086524 | 0.20523476 | Down |
| XLOC_007020 | 1.442010288 | 0.006093244 | 0.20523476 | Up |
| LMAN2L | -1.348632762 | 0.006102348 | 0.20523476 | Down |
| XLOC_002798 | 1.784754902 | 0.00610236 | 0.20523476 | Up |
| XLOC_002968 | 1.153666088 | 0.006102406 | 0.20523476 | Up |
| C8orf51 | 1.063497583 | 0.006107985 | 0.20523476 | Up |
| XLOC_007920 | 1.199432654 | 0.006110567 | 0.20523476 | Up |
| ATP5O | -1.090109875 | 0.006129028 | 0.20523476 | Down |
| OR4D10 | 2.035460621 | 0.006129635 | 0.20523476 | Up |
| VPS36 | -1.261525475 | 0.006150617 | 0.20523476 | Down |
| XLOC_007607 | 1.516193437 | 0.006157527 | 0.20523476 | Up |
| DEGS1 | -1.56875362 | 0.006166356 | 0.20523476 | Down |
| XLOC_000617 | 1.09112411 | 0.006173282 | 0.20523476 | Up |
| ABCD1 | 1.377417428 | 0.006177245 | 0.20523476 | Up |
| NID1 | -2.299992247 | 0.006185003 | 0.20523476 | Down |
| XLOC_009421 | 1.384708173 | 0.006193814 | 0.20523476 | Up |
| XLOC_000538 | 1.151473721 | 0.006194097 | 0.20523476 | Up |
| XLOC_004100 | 1.112889867 | 0.006195596 | 0.20523476 | Up |
| XLOC_010665 | 1.387439427 | 0.006198817 | 0.20523476 | Up |
| ZNF697 | 1.212196855 | 0.00620154 | 0.20523476 | Up |
| FAM200B | -1.006938187 | 0.006205378 | 0.20523476 | Down |
| TRAF5 | -1.432460249 | 0.006216438 | 0.20523476 | Down |
| XLOC_012922 | 1.914334313 | 0.006220497 | 0.20523476 | Up |
| TOR1AIP1 | -1.26506407 | 0.006224865 | 0.20523476 | Down |
| BAAT | -1.445578944 | 0.006229653 | 0.20523476 | Down |
| XLOC_013162 | 2.253566275 | 0.006235338 | 0.20523476 | Up |
| GALNT11 | -1.394253403 | 0.006243594 | 0.20523476 | Down |
| CDH8 | 1.479127849 | 0.006249204 | 0.20523476 | Up |
| XLOC_l2_015830 | 1.240698223 | 0.006251762 | 0.20523476 | Up |
| NUDT9 | -1.153568544 | 0.006253025 | 0.20523476 | Down |
| GTF2F2 | -1.084763427 | 0.006260505 | 0.20523476 | Down |
| F2RL2 | 1.300411549 | 0.006262434 | 0.20523476 | Up |
| XLOC_004408 | 1.574389023 | 0.00626737 | 0.20523476 | Up |
| C1orf70 | 1.192273482 | 0.006272063 | 0.20523476 | Up |
| RPL13 | -1.206073452 | 0.006275616 | 0.20523476 | Down |
| SETD7 | -1.054044576 | 0.006292986 | 0.20523476 | Down |
| XLOC_002177 | 1.23070777 | 0.006294049 | 0.20523476 | Up |
| CLN8 | -1.519977041 | 0.00630302 | 0.20523476 | Down |
| XLOC_004743 | 1.125737942 | 0.006323065 | 0.20523476 | Up |
| COL12A1 | -2.066237435 | 0.006340079 | 0.20523476 | Down |
| NICN1 | -1.111319548 | 0.006342172 | 0.20523476 | Down |
| ITPRIPL1 | 1.206532116 | 0.006342657 | 0.20523476 | Up |
| MRPS31 | -1.040520842 | 0.006351234 | 0.20523476 | Down |
| XLOC_012572 | 2.355173025 | 0.006375031 | 0.20523476 | Up |
| VEGFA | -1.161418284 | 0.006380365 | 0.20523476 | Down |
| XLOC_006383 | 1.172355271 | 0.006381911 | 0.20523476 | Up |
| XLOC_013884 | 1.642566488 | 0.006394756 | 0.20523476 | Up |
| XLOC_002263 | 1.496181742 | 0.006402764 | 0.20523476 | Up |
| SUN3 | 1.007732632 | 0.006407335 | 0.20523476 | Up |
| FNTA | -1.057444149 | 0.006430474 | 0.20523476 | Down |
| AKT3 | -1.202172837 | 0.006435813 | 0.20523476 | Down |
| ARMCX1 | -1.2505585 | 0.006440536 | 0.20523476 | Down |
| XLOC_007219 | 1.279653727 | 0.006443419 | 0.20523476 | Up |
| SGTB | -1.241076179 | 0.006459685 | 0.20523476 | Down |
| RWDD1 | -1.072119953 | 0.006460935 | 0.20523476 | Down |
| FZD7 | -1.767297332 | 0.006468033 | 0.20523476 | Down |
| XLOC_000917 | 1.436709587 | 0.006469195 | 0.20523476 | Up |
| FAM117A | -1.022038451 | 0.006474366 | 0.20523476 | Down |
| XLOC_l2_013582 | 1.107020293 | 0.00648709 | 0.20523476 | Up |
| EFEMP2 | -1.470787113 | 0.006491458 | 0.20523476 | Down |
| HSPB2 | -1.530724987 | 0.006509695 | 0.20523476 | Down |
| XLOC_012631 | 1.035776964 | 0.006554584 | 0.20523476 | Up |
| XLOC_013687 | 1.39240353 | 0.006563347 | 0.20523476 | Up |
| SGCB | -1.337870303 | 0.006563908 | 0.20523476 | Down |
| CFDP1 | -1.147166946 | 0.006574204 | 0.20523476 | Down |
| OR1S1 | 1.936662708 | 0.006579625 | 0.20523476 | Up |
| MEF2A | -1.375796146 | 0.006588169 | 0.20523476 | Down |
| XLOC_011081 | 1.192205974 | 0.006590011 | 0.20523476 | Up |
| DGKA | 1.408522341 | 0.006610448 | 0.20523476 | Up |
| XLOC_006991 | 1.194283726 | 0.006611894 | 0.20523476 | Up |
| ZMYM1 | -1.303433986 | 0.00662477 | 0.20523476 | Down |
| GNAI1 | -1.626325645 | 0.006624937 | 0.20523476 | Down |
| DAB2 | -1.325403826 | 0.006643282 | 0.20523476 | Down |
| XLOC_l2_015315 | 1.947022881 | 0.006663365 | 0.20523476 | Up |
| VRTN | 2.063132313 | 0.006666168 | 0.20523476 | Up |
| KIT | -1.283623651 | 0.00667821 | 0.20523476 | Down |
| BPIFA2 | 1.141443394 | 0.006680636 | 0.20523476 | Up |
| XLOC_002411 | 1.35145028 | 0.006681306 | 0.20523476 | Up |
| SNORD116-4 | -1.311532406 | 0.006687806 | 0.20523476 | Down |
| SLC22A6 | 1.232381626 | 0.006694072 | 0.20523476 | Up |
| CEP112 | -1.087239801 | 0.006694754 | 0.20523476 | Down |
| XLOC_001072 | 1.093424939 | 0.006700662 | 0.20523476 | Up |
| FAM190B | -1.263645114 | 0.006706771 | 0.20523476 | Down |
| PIGZ | -1.492601271 | 0.006721584 | 0.20523476 | Down |
| CALM3 | -1.175504552 | 0.006722935 | 0.20523476 | Down |
| XLOC_004105 | 1.145579241 | 0.006733841 | 0.20523476 | Up |
| SPP2 | 1.225951829 | 0.006743607 | 0.20523476 | Up |
| XLOC_003048 | 2.9460025 | 0.006748689 | 0.20523476 | Up |
| PDHA2 | 1.995643784 | 0.006754185 | 0.20523476 | Up |
| XLOC_003656 | 1.004230541 | 0.006760277 | 0.20523476 | Up |
| ICMT | -1.382188428 | 0.006776909 | 0.20523476 | Down |
| XLOC_000470 | 2.391645589 | 0.006782732 | 0.20523476 | Up |
| XLOC_001947 | 1.115818976 | 0.006796181 | 0.20523476 | Up |
| DKFZP434H168 | 1.334893719 | 0.006798666 | 0.20523476 | Up |
| XKR7 | 2.086916298 | 0.006812532 | 0.20523476 | Up |
| GNN | 1.464338298 | 0.006812645 | 0.20523476 | Up |
| PAPOLG | -1.14218624 | 0.006830164 | 0.20523476 | Down |
| FANCL | -1.057657228 | 0.006835491 | 0.20523476 | Down |
| XLOC_009493 | 1.333725131 | 0.006843498 | 0.20523476 | Up |
| XLOC_009605 | 1.162053184 | 0.006843622 | 0.20523476 | Up |
| ANXA5 | -1.262659317 | 0.006845485 | 0.20523476 | Down |
| CSN1S2AP | 1.053029327 | 0.00685855 | 0.20523476 | Up |
| XLOC_001546 | 1.27023207 | 0.00686405 | 0.20523476 | Up |
| XLOC_003560 | 1.174884988 | 0.006867229 | 0.20523476 | Up |
| LINC00294 | -1.288803612 | 0.00687093 | 0.20523476 | Down |
| PHLDB1 | -1.341117118 | 0.006879872 | 0.20523476 | Down |
| TATDN2 | -1.047492284 | 0.006886222 | 0.20523476 | Down |
| XLOC_004914 | 1.340089861 | 0.006890284 | 0.20523476 | Up |
| XLOC_006814 | 1.464608288 | 0.006892188 | 0.20523476 | Up |
| RNASE13 | 1.219789975 | 0.006895246 | 0.20523476 | Up |
| XLOC_010972 | 1.520807387 | 0.006921497 | 0.20523476 | Up |
| HIST1H2AE | -1.311749193 | 0.006942935 | 0.20523476 | Down |
| CCNT1 | -1.049405614 | 0.006957527 | 0.20523476 | Down |
| ZER1 | -1.149870929 | 0.006960704 | 0.20523476 | Down |
| TCTN1 | -1.178367446 | 0.006967863 | 0.20523476 | Down |
| MYO15A | 1.170285505 | 0.006973755 | 0.20523476 | Up |
| C9orf93 | -1.284827116 | 0.006977362 | 0.20523476 | Down |
| FAM198A | -1.226966761 | 0.006992168 | 0.20523476 | Down |
| XLOC_005461 | 1.595904148 | 0.006995532 | 0.20523476 | Up |
| BMI1 | -1.16810078 | 0.00699562 | 0.20523476 | Down |
| SELV | 1.721319124 | 0.006998296 | 0.20523476 | Up |
| PLSCR5 | 1.491351775 | 0.007024766 | 0.20523476 | Up |
| XLOC_014172 | 1.329299313 | 0.007067478 | 0.20523476 | Up |
| CDK14 | -1.129812705 | 0.007099537 | 0.20523476 | Down |
| OVCH1 | 1.555347103 | 0.007102923 | 0.20523476 | Up |
| MDGA1 | 1.594480638 | 0.007133971 | 0.20523476 | Up |
| LEMD3 | -1.102443 | 0.007134687 | 0.20523476 | Down |
| ZG16 | 1.011014115 | 0.007137216 | 0.20523476 | Up |
| XLOC_004219 | 1.361859745 | 0.007143787 | 0.20523476 | Up |
| HMCN1 | -1.201540777 | 0.007145576 | 0.20523476 | Down |
| DIXDC1 | -1.758491685 | 0.007149685 | 0.20523476 | Down |
| XLOC_011807 | 1.187831394 | 0.007150392 | 0.20523476 | Up |
| SLC34A3 | 1.100274173 | 0.007151423 | 0.20523476 | Up |
| MED4 | -1.006790618 | 0.007161494 | 0.20523476 | Down |
| ABCB7 | -1.158986767 | 0.007167609 | 0.20523476 | Down |
| CLDN12 | -1.072693367 | 0.007194018 | 0.20523476 | Down |
| ASGR1 | 1.400324561 | 0.007213037 | 0.20523476 | Up |
| XLOC_005910 | 1.157670647 | 0.007214428 | 0.20523476 | Up |
| COX17 | -1.104558058 | 0.007236866 | 0.20523476 | Down |
| CEP78 | -1.151287853 | 0.007248049 | 0.20523476 | Down |
| ARHGEF6 | -1.476635164 | 0.007263387 | 0.20523476 | Down |
| C5orf13 | -1.452995813 | 0.007281912 | 0.20523476 | Down |
| XLOC_004025 | 1.068910271 | 0.007292226 | 0.20523476 | Up |
| FAU | -1.262743299 | 0.00729586 | 0.20523476 | Down |
| MAP3K15 | -1.365881226 | 0.00730006 | 0.20523476 | Down |
| HES2 | 1.106519932 | 0.007306052 | 0.20523476 | Up |
| TMEM155 | 1.501285707 | 0.007308266 | 0.20523476 | Up |
| HIST1H2BD | -1.158857998 | 0.007310618 | 0.20523476 | Down |
| CNTN3 | -1.12591548 | 0.007313479 | 0.20523476 | Down |
| XLOC_003770 | 1.397445376 | 0.007327216 | 0.20523476 | Up |
| XLOC_000972 | 1.188279025 | 0.007329065 | 0.20523476 | Up |
| XLOC_003833 | 1.788333377 | 0.007340522 | 0.20523476 | Up |
| PPP1R3E | -1.113328122 | 0.007341959 | 0.20523476 | Down |
| XLOC_013909 | 1.144035894 | 0.00735111 | 0.20523476 | Up |
| RMRP | -1.343871878 | 0.007362866 | 0.20523476 | Down |
| KRT79 | 1.701521675 | 0.007377592 | 0.20523476 | Up |
| XLOC_013653 | 1.096185726 | 0.007379225 | 0.20523476 | Up |
| FBL | -1.221710388 | 0.007380059 | 0.20523476 | Down |
| XLOC_003314 | 1.622644772 | 0.00740418 | 0.20523476 | Up |
| XLOC_001877 | 1.225162612 | 0.007414297 | 0.20523476 | Up |
| XLOC_004587 | 1.005243311 | 0.007428855 | 0.20523476 | Up |
| REV3L | -1.376325651 | 0.007443789 | 0.20523476 | Down |
| SKA2 | -1.33318968 | 0.007445316 | 0.20523476 | Down |
| XLOC_012829 | 2.032420968 | 0.007462538 | 0.20523476 | Up |
| RABAC1 | -1.285207281 | 0.007463622 | 0.20523476 | Down |
| NCOR2 | -1.062422626 | 0.007463892 | 0.20523476 | Down |
| LAMB2 | -1.302430849 | 0.007467289 | 0.20523476 | Down |
| LARS2 | -1.270339264 | 0.007473695 | 0.20523476 | Down |
| TSHZ3 | -2.223221436 | 0.007485899 | 0.20523476 | Down |
| MALAT1 | -1.097538035 | 0.007503556 | 0.20523476 | Down |
| XLOC_005460 | 1.102362417 | 0.007528073 | 0.20523476 | Up |
| FAM184B | -1.140508447 | 0.007529125 | 0.20523476 | Down |
| LSM7 | -1.045351548 | 0.007535083 | 0.20523476 | Down |
| MAP1B | -1.59978328 | 0.007536492 | 0.20523476 | Down |
| XLOC_001455 | 1.294708501 | 0.007540041 | 0.20523476 | Up |
| BIVM | -1.125177796 | 0.007549307 | 0.20523476 | Down |
| GSG1L | 1.521040778 | 0.007568326 | 0.20523476 | Up |
| AHSG | 1.450102899 | 0.007569874 | 0.20523476 | Up |
| XLOC_005453 | 1.426935776 | 0.007569971 | 0.20523476 | Up |
| LIX1L | -1.617537382 | 0.007580245 | 0.20523476 | Down |
| SHISA3 | -2.167650837 | 0.007587051 | 0.20523476 | Down |
| WDR67 | -1.298193936 | 0.007591524 | 0.20523476 | Down |
| RAB3GAP1 | -1.015831694 | 0.007595607 | 0.20523476 | Down |
| ZC3H8 | -1.106364414 | 0.007605751 | 0.20523476 | Down |
| RPS10 | -1.346489684 | 0.007607525 | 0.20523476 | Down |
| SOST | 1.616508999 | 0.007609457 | 0.20523476 | Up |
| GLI1 | -1.989394523 | 0.007625827 | 0.20523476 | Down |
| MYL6 | -1.003729801 | 0.007638609 | 0.20523476 | Down |
| XLOC_004959 | 2.376282386 | 0.007640917 | 0.20523476 | Up |
| RABEP2 | -1.028400398 | 0.007643588 | 0.20523476 | Down |
| AIF1 | 2.095404123 | 0.007659966 | 0.20523476 | Up |
| XLOC_003837 | 1.341072744 | 0.007666835 | 0.20523476 | Up |
| XLOC_010348 | 1.49914931 | 0.007677852 | 0.20523476 | Up |
| XLOC_006193 | 1.396449649 | 0.007679436 | 0.20523476 | Up |
| XLOC_010918 | 1.026141891 | 0.007697919 | 0.20523476 | Up |
| XLOC_004431 | 1.2567845 | 0.007700241 | 0.20523476 | Up |
| HGSNAT | -1.085020384 | 0.007703247 | 0.20523476 | Down |
| XLOC_007089 | 1.130527822 | 0.007715649 | 0.20523476 | Up |
| XLOC_003484 | 1.677254078 | 0.007719086 | 0.20523476 | Up |
| XLOC_013681 | 1.121189882 | 0.00772351 | 0.20523476 | Up |
| UBA52 | -1.02663485 | 0.007725125 | 0.20523476 | Down |
| C18orf1 | -1.00634623 | 0.007734657 | 0.20523476 | Down |
| XYLT2 | -1.216876965 | 0.007737709 | 0.20523476 | Down |
| SMTN | -1.725800058 | 0.007743779 | 0.20523476 | Down |
| HIST2H4B | -1.109929688 | 0.00776001 | 0.20523476 | Down |
| CFD | -2.013645368 | 0.00776972 | 0.20523476 | Down |
| XLOC_008587 | 1.38864554 | 0.007769928 | 0.20523476 | Up |
| CNKSR3 | -1.277054496 | 0.007788411 | 0.20523476 | Down |
| XLOC_007681 | 1.99466535 | 0.007792026 | 0.20523476 | Up |
| OAZ2 | -1.203976044 | 0.007793555 | 0.20523476 | Down |
| ZNF516 | -1.421208556 | 0.007799697 | 0.20523476 | Down |
| XLOC_014174 | 1.505893517 | 0.007803772 | 0.20523476 | Up |
| XLOC_002172 | 1.404119234 | 0.007830552 | 0.20523476 | Up |
| XLOC_010151 | 1.13495708 | 0.007832809 | 0.20523476 | Up |
| XLOC_006680 | 1.354305705 | 0.007836732 | 0.20523476 | Up |
| SNORD115-27 | 1.473933386 | 0.007841418 | 0.20523476 | Up |
| XLOC_009625 | 1.072251711 | 0.007842753 | 0.20523476 | Up |
| HIST1H2BC | -1.145983936 | 0.007843807 | 0.20523476 | Down |
| SEPT10 | -1.187865158 | 0.007847038 | 0.20523476 | Down |
| LINC00112 | 1.314552527 | 0.007850848 | 0.20523476 | Up |
| XLOC_l2_000186 | 1.268933947 | 0.007858608 | 0.20523476 | Up |
| XLOC_007431 | 1.388037698 | 0.007866696 | 0.20523476 | Up |
| XLOC_006818 | 2.159338623 | 0.007875213 | 0.20523476 | Up |
| SETD4 | -1.401142181 | 0.007875837 | 0.20523476 | Down |
| SH3D19 | -1.06262219 | 0.00788963 | 0.20523476 | Down |
| XLOC_004123 | 1.315648889 | 0.007891081 | 0.20523476 | Up |
| EPC1 | -1.170113559 | 0.007894781 | 0.20523476 | Down |
| AQP10 | 1.430205963 | 0.007910422 | 0.20523476 | Up |
| RPL34 | -1.02217259 | 0.007925791 | 0.20523476 | Down |
| C6orf48 | -1.396939468 | 0.007930754 | 0.20523476 | Down |
| XLOC_012662 | 1.679171155 | 0.007936081 | 0.20523476 | Up |
| NMBR | 1.555200825 | 0.007941885 | 0.20523476 | Up |
| SYN3 | 1.229280373 | 0.007957743 | 0.20523476 | Up |
| XLOC_l2_015876 | 1.855112349 | 0.007961739 | 0.20523476 | Up |
| SPOP | -1.023737814 | 0.007963024 | 0.20523476 | Down |
| XLOC_013832 | 1.226379387 | 0.00796956 | 0.20523476 | Up |
| DBN1 | -1.417607693 | 0.00797494 | 0.20523476 | Down |
| CXCR7 | -1.16009555 | 0.007979279 | 0.20523476 | Down |
| COL14A1 | -2.187136573 | 0.008005442 | 0.20523476 | Down |
| PIGV | -1.197277131 | 0.008014138 | 0.20523476 | Down |
| OR2M3 | 1.780360139 | 0.008018615 | 0.20523476 | Up |
| OR5I1 | 1.992674152 | 0.008018707 | 0.20523476 | Up |
| MEF2C | -1.347617037 | 0.008019144 | 0.20523476 | Down |
| XLOC_005133 | 1.936129427 | 0.008039998 | 0.20523476 | Up |
| XLOC_009340 | 1.204879722 | 0.008048581 | 0.20523476 | Up |
| HEPH | -1.368188371 | 0.008069749 | 0.20523476 | Down |
| MRPL22 | -1.109996154 | 0.008072287 | 0.20523476 | Down |
| XLOC_l2_007184 | 1.23937614 | 0.00807298 | 0.20523476 | Up |
| FN1 | -1.863182425 | 0.008073027 | 0.20523476 | Down |
| FAM198B | -1.902853227 | 0.00807898 | 0.20523476 | Down |
| PLEKHA6 | -1.028363537 | 0.008086927 | 0.20523476 | Down |
| HSFY2 | 2.273993099 | 0.008088264 | 0.20523476 | Up |
| XLOC_003220 | 1.135993532 | 0.008098435 | 0.20523476 | Up |
| XLOC_007689 | 1.776063667 | 0.008127424 | 0.20523476 | Up |
| XLOC_004611 | 1.619534886 | 0.008156819 | 0.20523476 | Up |
| ASNSD1 | -1.237835675 | 0.008158854 | 0.20523476 | Down |
| XLOC_012677 | 1.170813429 | 0.008158969 | 0.20523476 | Up |
| XLOC_000392 | 1.005084274 | 0.008174073 | 0.20523476 | Up |
| TMEM167B | -1.12988612 | 0.008203943 | 0.20523476 | Down |
| CNBP | -1.27661287 | 0.008210509 | 0.20523476 | Down |
| CD177 | 1.19796776 | 0.008211551 | 0.20523476 | Up |
| KRT6A | 1.610069195 | 0.008216743 | 0.20523476 | Up |
| XLOC_l2_006080 | 1.178806291 | 0.008221559 | 0.20523476 | Up |
| XLOC_007855 | 1.070063864 | 0.008228753 | 0.20523476 | Up |
| XLOC_l2_013848 | 1.073179671 | 0.008244435 | 0.20523476 | Up |
| TACO1 | -1.14084197 | 0.008259819 | 0.20523476 | Down |
| MITD1 | -1.106551154 | 0.008266565 | 0.20523476 | Down |
| ACTA2 | -2.768275581 | 0.0082671 | 0.20523476 | Down |
| AFAP1 | -1.21947116 | 0.008268615 | 0.20523476 | Down |
| TIMM9 | -1.238866697 | 0.008280232 | 0.20523476 | Down |
| XLOC_011661 | 1.545668377 | 0.008293574 | 0.20523476 | Up |
| XLOC_003344 | 1.442438889 | 0.008296663 | 0.20523476 | Up |
| C5orf28 | -1.004621243 | 0.008296899 | 0.20523476 | Down |
| OR10A5 | 1.508947135 | 0.00829726 | 0.20523476 | Up |
| KLRD1 | -2.193996114 | 0.008298272 | 0.20523476 | Down |
| RAB9BP1 | -1.13719657 | 0.008299168 | 0.20523476 | Down |
| ZSCAN5B | 1.256273988 | 0.008309853 | 0.20523476 | Up |
| WDR82 | -1.134781616 | 0.008311385 | 0.20523476 | Down |
| XLOC_011971 | 1.109871921 | 0.008325812 | 0.20523476 | Up |
| XLOC_000781 | 1.208711309 | 0.008337467 | 0.20523476 | Up |
| CCDC91 | 1.219607187 | 0.008374937 | 0.20523476 | Up |
| PDK3 | -1.26201565 | 0.008375354 | 0.20523476 | Down |
| TAS2R20 | -1.429017056 | 0.008384871 | 0.20523476 | Down |
| RPS19 | -1.126212633 | 0.008385341 | 0.20523476 | Down |
| XLOC_005057 | 1.070958525 | 0.008393027 | 0.20523476 | Up |
| LINGO4 | 1.880755016 | 0.00839652 | 0.20523476 | Up |
| TMEM42 | -1.18582135 | 0.00839902 | 0.20523476 | Down |
| RPS15A | -1.371072937 | 0.008401109 | 0.20523476 | Down |
| XLOC_006270 | 1.03072061 | 0.008412354 | 0.20523476 | Up |
| OR51D1 | 1.091491421 | 0.008413642 | 0.20523476 | Up |
| PPP3CC | -1.492779219 | 0.008424347 | 0.20523476 | Down |
| NAP1L3 | -1.44368702 | 0.008432352 | 0.20523476 | Down |
| XLOC_001363 | 1.21973722 | 0.008443116 | 0.20523476 | Up |
| XLOC_l2_004843 | 1.406522882 | 0.008454672 | 0.20523476 | Up |
| SPRY1 | -1.36067579 | 0.008457303 | 0.20523476 | Down |
| DNAJC15 | -1.048754568 | 0.008479049 | 0.20523476 | Down |
| CD302 | -1.38622609 | 0.00850247 | 0.20523476 | Down |
| OR10P1 | 2.536626799 | 0.00851688 | 0.20523476 | Up |
| RPRML | 1.571912214 | 0.008517946 | 0.20523476 | Up |
| NR2F2 | -1.640515525 | 0.008525157 | 0.20523476 | Down |
| XLOC_012561 | 1.343499786 | 0.008533644 | 0.20523476 | Up |
| MEIS2 | -1.937995877 | 0.008553109 | 0.20523476 | Down |
| DRD3 | 1.090006 | 0.008571726 | 0.20523476 | Up |
| CX3CR1 | -1.458710632 | 0.008574805 | 0.20523476 | Down |
| FAM120B | -1.308063849 | 0.008583532 | 0.20523476 | Down |
| DNAJC1 | -1.045046842 | 0.008586854 | 0.20523476 | Down |
| SLC5A11 | 1.23962463 | 0.008602199 | 0.20523476 | Up |
| XLOC_005132 | 1.210981527 | 0.008609509 | 0.20523476 | Up |
| XLOC_005276 | 1.718379062 | 0.008621902 | 0.20523476 | Up |
| FECH | -1.222953593 | 0.008630569 | 0.20523476 | Down |
| XLOC_010893 | 1.508301519 | 0.008636331 | 0.20523476 | Up |
| C13orf27 | -1.168365852 | 0.00866636 | 0.20523476 | Down |
| DENND4B | -1.198609652 | 0.008677385 | 0.20523476 | Down |
| MAGEA1 | 1.969408503 | 0.0086919 | 0.20523476 | Up |
| BLVRB | -1.201921202 | 0.008704277 | 0.20523476 | Down |
| MAOB | -2.087607502 | 0.008711397 | 0.20523476 | Down |
| SAE1 | -1.077307104 | 0.008718347 | 0.20523476 | Down |
| XLOC_004022 | 1.441519067 | 0.008732541 | 0.20523476 | Up |
| C3orf74 | 1.924797364 | 0.008764248 | 0.20523476 | Up |
| XLOC_003548 | 1.253808418 | 0.008767405 | 0.20523476 | Up |
| XLOC_013960 | 1.227794605 | 0.008775001 | 0.20523476 | Up |
| KCNJ10 | 1.454027487 | 0.008778279 | 0.20523476 | Up |
| XLOC_008702 | 1.185276202 | 0.00878202 | 0.20523476 | Up |
| XLOC_011157 | 1.159218616 | 0.00880303 | 0.20523476 | Up |
| MYEOV | 1.057831144 | 0.008808642 | 0.20523476 | Up |
| NCOA6 | -1.044860253 | 0.008825411 | 0.20523476 | Down |
| PRTFDC1 | -1.736860993 | 0.008838573 | 0.20523476 | Down |
| XLOC_001903 | 1.167812002 | 0.008839467 | 0.20523476 | Up |
| PLDN | -1.009827876 | 0.008852302 | 0.20523476 | Down |
| HOTAIRM1 | -1.187038711 | 0.008865674 | 0.20523476 | Down |
| HELZ | -1.468605253 | 0.00887403 | 0.20523476 | Down |
| PP7080 | -1.678215606 | 0.008874931 | 0.20523476 | Down |
| XLOC_005465 | 1.714597959 | 0.008877418 | 0.20523476 | Up |
| XLOC_008481 | 1.49493038 | 0.008882708 | 0.20523476 | Up |
| ENTPD3-AS1 | 1.605674763 | 0.008908626 | 0.20523476 | Up |
| XLOC_011317 | 1.610529048 | 0.008962714 | 0.20523476 | Up |
| UBQLN1 | -1.074479669 | 0.008964754 | 0.20523476 | Down |
| GSK3A | -1.169488835 | 0.008965908 | 0.20523476 | Down |
| CHST7 | -1.144899697 | 0.008971273 | 0.20523476 | Down |
| PXDN | -1.990129999 | 0.008971689 | 0.20523476 | Down |
| ECSCR | -1.55854763 | 0.008995845 | 0.20523476 | Down |
| ADH5 | -1.567294359 | 0.009007992 | 0.20523476 | Down |
| KLHL24 | -1.262738818 | 0.009043959 | 0.20523476 | Down |
| XLOC_009250 | 2.44263086 | 0.009057389 | 0.20523476 | Up |
| XLOC_001852 | 1.143390328 | 0.009058138 | 0.20523476 | Up |
| CDKN1B | -1.17657887 | 0.009064206 | 0.20523476 | Down |
| XLOC_014237 | 1.022043956 | 0.009074218 | 0.20523476 | Up |
| CRHR2 | 1.030613568 | 0.009085535 | 0.20523476 | Up |
| WIZ | -1.112920291 | 0.009086186 | 0.20523476 | Down |
| CSGALNACT1 | -1.597409365 | 0.009086488 | 0.20523476 | Down |
| EIF5AL1 | 1.128026918 | 0.009087013 | 0.20523476 | Up |
| HIST2H2AA4 | -1.558434964 | 0.009092109 | 0.20523476 | Down |
| C16orf90 | 1.124105843 | 0.009094543 | 0.20523476 | Up |
| HIST1H4K | -1.107748975 | 0.009102306 | 0.20523476 | Down |
| C2orf68 | -1.253508344 | 0.009109091 | 0.20523476 | Down |
| RGSL1 | 1.09758874 | 0.009117014 | 0.20523476 | Up |
| XLOC_006008 | 1.735440311 | 0.009118859 | 0.20523476 | Up |
| CSRP2BP | -1.25373327 | 0.009123506 | 0.20523476 | Down |
| ARMCX6 | -1.132863399 | 0.009124322 | 0.20523476 | Down |
| XLOC_010301 | 1.034124166 | 0.009132154 | 0.20523476 | Up |
| XLOC_008298 | 1.319743731 | 0.009143567 | 0.20523476 | Up |
| MRAS | -1.679507704 | 0.009148655 | 0.20523476 | Down |
| XLOC_005117 | 1.786246321 | 0.009150072 | 0.20523476 | Up |
| OSR2 | -2.014178804 | 0.009161543 | 0.20523476 | Down |
| XLOC_008993 | 1.391738575 | 0.009200826 | 0.20523476 | Up |
| NPBWR2 | 2.074819777 | 0.009201638 | 0.20523476 | Up |
| XLOC_012979 | 1.640517676 | 0.00920482 | 0.20523476 | Up |
| ITGB8 | -1.542930737 | 0.009213508 | 0.20523476 | Down |
| GLB1 | -1.141839183 | 0.009232772 | 0.20523476 | Down |
| XLOC_l2_007907 | 1.102040146 | 0.009233633 | 0.20523476 | Up |
| SEPT3 | 1.154504159 | 0.009258223 | 0.20523476 | Up |
| XLOC_l2_014034 | 1.529670976 | 0.009268974 | 0.20523476 | Up |
| KRTAP15-1 | 1.805099073 | 0.009275245 | 0.20523476 | Up |
| CNPY1 | 1.495997776 | 0.009276567 | 0.20523476 | Up |
| XLOC_l2_000384 | 1.218999412 | 0.009283977 | 0.20523476 | Up |
| XLOC_001655 | 1.88848325 | 0.009286432 | 0.20523476 | Up |
| CKB | -2.058559032 | 0.009295943 | 0.20523476 | Down |
| COL9A2 | 1.807559422 | 0.009296373 | 0.20523476 | Up |
| XLOC_013031 | 1.147437651 | 0.009310831 | 0.20523476 | Up |
| PARVA | -1.114883669 | 0.009312117 | 0.20523476 | Down |
| ATG2B | -1.2774838 | 0.009321443 | 0.20523476 | Down |
| XLOC_013370 | 1.161269827 | 0.009322941 | 0.20523476 | Up |
| OR4N2 | 1.169554411 | 0.009327977 | 0.20523476 | Up |
| ZFAND2A | -1.014363994 | 0.009341883 | 0.20523476 | Down |
| XLOC_l2_014664 | 1.361699407 | 0.009355078 | 0.20523476 | Up |
| COMMD2 | -1.033387067 | 0.009368968 | 0.20523476 | Down |
| TMEM45A | -1.87901091 | 0.009369001 | 0.20523476 | Down |
| SNHG5 | -1.2874726 | 0.009380366 | 0.20523476 | Down |
| XLOC_002805 | 1.415940572 | 0.00938772 | 0.20523476 | Up |
| PFDN5 | -1.13331706 | 0.009408728 | 0.20523476 | Down |
| CYP4B1 | 1.769008369 | 0.009443683 | 0.20523476 | Up |
| TRIM49 | 1.460617304 | 0.009449738 | 0.20523476 | Up |
| XLOC_000822 | 1.159740027 | 0.009464597 | 0.20523476 | Up |
| HSPB7 | -1.404138701 | 0.009481063 | 0.20523476 | Down |
| MARCH6 | -1.170943587 | 0.009488316 | 0.20523476 | Down |
| XLOC_002342 | 1.176472447 | 0.009516538 | 0.20523476 | Up |
| XLOC_012120 | 2.319179917 | 0.009516748 | 0.20523476 | Up |
| COX7B | -1.024242193 | 0.009521769 | 0.20523476 | Down |
| MCPH1 | -1.016384135 | 0.00952665 | 0.20523476 | Down |
| TNS1 | -1.347114818 | 0.009530178 | 0.20523476 | Down |
| XLOC_013711 | 1.848079279 | 0.009532499 | 0.20523476 | Up |
| AGT | -2.134688061 | 0.009536899 | 0.20523476 | Down |
| XLOC_001632 | 1.639587456 | 0.009542113 | 0.20523476 | Up |
| ZNF721 | -1.173437379 | 0.009544595 | 0.20523476 | Down |
| KCNJ8 | -2.17630317 | 0.00954835 | 0.20523476 | Down |
| SPANXA1 | 1.916976924 | 0.009548872 | 0.20523476 | Up |
| XLOC_l2_012919 | 2.994763662 | 0.009553922 | 0.20523476 | Up |
| XLOC_l2_013302 | 1.270584138 | 0.00955942 | 0.20523476 | Up |
| GPR182 | 1.315895868 | 0.009564538 | 0.20523476 | Up |
| TMED10 | -1.139852325 | 0.009569448 | 0.20523476 | Down |
| MSRB3 | -2.004016451 | 0.009570704 | 0.20523476 | Down |
| SULT1C2P1 | 1.002013519 | 0.009580483 | 0.20523476 | Up |
| XLOC_002872 | -1.492332642 | 0.009584349 | 0.20523476 | Down |
| Q8WY88 | -1.624188421 | 0.009600552 | 0.20523476 | Down |
| XLOC_012819 | 1.541131615 | 0.009611224 | 0.20523476 | Up |
| RNF208 | -1.313861087 | 0.009625991 | 0.20523476 | Down |
| CRTAP | -1.486077731 | 0.009631359 | 0.20523476 | Down |
| XLOC_l2_001954 | 1.479634869 | 0.009634895 | 0.20523476 | Up |
| AOX1 | -1.329714246 | 0.009655385 | 0.20523476 | Down |
| ACR | 1.58629506 | 0.009671255 | 0.20523476 | Up |
| PRR23B | 1.435056738 | 0.009676511 | 0.20523476 | Up |
| BRD2 | -1.045402159 | 0.009677392 | 0.20523476 | Down |
| OR1S2 | 1.698508042 | 0.009679939 | 0.20523476 | Up |
| C6orf64 | -1.090887311 | 0.009697053 | 0.20523476 | Down |
| XLOC_004231 | 1.855174593 | 0.009708879 | 0.20523476 | Up |
| LEP | 1.692738214 | 0.009713403 | 0.20523476 | Up |
| ACVR2B | -1.16964911 | 0.00971408 | 0.20523476 | Down |
| XLOC_000624 | 1.644347866 | 0.009727886 | 0.20523476 | Up |
| FSCN3 | 1.03763634 | 0.009733164 | 0.20523476 | Up |
| NPHP3 | -1.070732129 | 0.009737173 | 0.20523476 | Down |
| KRTAP21-1 | 1.068588079 | 0.00976147 | 0.20523476 | Up |
| ATP6V1C1 | -1.130405806 | 0.009763766 | 0.20523476 | Down |
| XLOC_006105 | 2.171823411 | 0.009773479 | 0.20523476 | Up |
| XLOC_009274 | 1.300892756 | 0.00978078 | 0.20523476 | Up |
| RNASE8 | 1.812540412 | 0.009782803 | 0.20523476 | Up |
| XLOC_l2_007237 | 1.016957895 | 0.009799889 | 0.20523476 | Up |
| ZNF845 | -1.188660032 | 0.009808324 | 0.20523476 | Down |
| BMP15 | 1.23107281 | 0.009810731 | 0.20523476 | Up |
| PEX11B | -1.153784728 | 0.009821404 | 0.20523476 | Down |
| PLAGL2 | 1.13323927 | 0.009870343 | 0.20523476 | Up |
| XLOC_009974 | 1.680666645 | 0.009881969 | 0.20523476 | Up |
| C2orf83 | 1.059748429 | 0.009899901 | 0.20523476 | Up |
| XLOC_001516 | 1.6862908 | 0.009907414 | 0.20523476 | Up |
| ADRBK2 | -1.146909528 | 0.009907497 | 0.20523476 | Down |
| SCLT1 | -1.112001835 | 0.009911835 | 0.20523476 | Down |
| XLOC_006188 | 1.941975041 | 0.009913498 | 0.20523476 | Up |
| PGCP | -1.029920642 | 0.009916611 | 0.20523476 | Down |
| XLOC_007268 | 1.435992897 | 0.009920083 | 0.20523476 | Up |
| KLB | 1.069557042 | 0.009924756 | 0.20523476 | Up |
| XLOC_009202 | 1.459970032 | 0.009931981 | 0.20523476 | Up |
| XLOC_007322 | 1.165860208 | 0.009947981 | 0.20523476 | Up |
| XLOC_013566 | 1.614564569 | 0.009968036 | 0.20523476 | Up |
| FAM26F | -1.179853817 | 0.009975209 | 0.20523476 | Down |
| UPF1 | -1.063661087 | 0.009994241 | 0.20523476 | Down |
| CRTC3 | -1.077287368 | 0.01000165 | 0.20523476 | Down |
| ARHGAP6 | -1.369241952 | 0.010042069 | 0.20523476 | Down |
| C21orf33 | -1.011177094 | 0.010054635 | 0.20523476 | Down |
| KCNV2 | 1.214245393 | 0.01005547 | 0.20523476 | Up |
| OR2AE1 | 1.012530622 | 0.010060261 | 0.20523476 | Up |
| XLOC_l2_001771 | 1.124851396 | 0.0100613 | 0.20523476 | Up |
| XLOC_010686 | 1.148494406 | 0.010061872 | 0.20523476 | Up |
| RBMS3 | -1.250995475 | 0.010064714 | 0.20523476 | Down |
| AES | -1.108891229 | 0.010070997 | 0.20523476 | Down |
| MAPRE3 | -1.042003986 | 0.010074255 | 0.20523476 | Down |
| CDHR5 | 1.716059302 | 0.010096606 | 0.20523476 | Up |
| XLOC_l2_004318 | -1.548098273 | 0.010102003 | 0.20523476 | Down |
| HIST1H2BI | -1.114305095 | 0.01011986 | 0.20523476 | Down |
| NUP205 | -1.19831805 | 0.010122653 | 0.20523476 | Down |
| FAM114A1 | -1.150777564 | 0.010128665 | 0.20523476 | Down |
| HEXB | -1.122942518 | 0.010130798 | 0.20523476 | Down |
| CNPY4 | -1.087198554 | 0.010150293 | 0.20523476 | Down |
| TMEM95 | 1.526666345 | 0.010151135 | 0.20523476 | Up |
| PROL1 | 2.765503313 | 0.010158234 | 0.20523476 | Up |
| TAF9B | -1.283317807 | 0.010161737 | 0.20523476 | Down |
| XLOC_006154 | 1.142692408 | 0.010185962 | 0.20523476 | Up |
| C10orf62 | 1.151563998 | 0.010189958 | 0.20523476 | Up |
| ACTR3BP2 | 1.45423628 | 0.01019106 | 0.20523476 | Up |
| PRNP | -1.186728519 | 0.010193834 | 0.20523476 | Down |
| KCNE1 | 1.247311847 | 0.010207093 | 0.20523476 | Up |
| KPNA5 | -1.295156955 | 0.010213256 | 0.20523476 | Down |
| XLOC_000955 | 1.107140268 | 0.010217506 | 0.20523476 | Up |
| TMEM190 | 1.067899101 | 0.010218094 | 0.20523476 | Up |
| XLOC_007035 | 1.710014912 | 0.010237162 | 0.20523476 | Up |
| ARHGAP28 | -1.081814669 | 0.010262265 | 0.20523476 | Down |
| FOXD4L2 | 1.077306627 | 0.010268781 | 0.20523476 | Up |
| XLOC_011937 | 1.463316262 | 0.010276663 | 0.20523476 | Up |
| ACD | -1.000089176 | 0.010280495 | 0.20523476 | Down |
| SIX5 | -1.083532593 | 0.010289645 | 0.20523476 | Down |
| MID1IP1 | -1.509360122 | 0.010294856 | 0.20523476 | Down |
| TMEM133 | -1.313197647 | 0.010313163 | 0.20523476 | Down |
| MYO18B | 1.071420176 | 0.010316076 | 0.20523476 | Up |
| XLOC_002714 | 1.281240764 | 0.010327151 | 0.20523476 | Up |
| APCDD1 | -1.893179777 | 0.010339573 | 0.20523476 | Down |
| ARHGAP1 | -1.151849268 | 0.010341405 | 0.20523476 | Down |
| OR6N2 | 1.84241814 | 0.010376171 | 0.20523476 | Up |
| ALG9 | -1.277189174 | 0.010388127 | 0.20523476 | Down |
| CAT | -1.150234467 | 0.010390938 | 0.20523476 | Down |
| CGGBP1 | -1.103722934 | 0.010419562 | 0.20523476 | Down |
| CACNG3 | 2.231914016 | 0.010427201 | 0.20523476 | Up |
| XLOC_006043 | 1.540305298 | 0.010430809 | 0.20523476 | Up |
| NGFRAP1 | -1.143392897 | 0.010431011 | 0.20523476 | Down |
| OR51T1 | 1.324427371 | 0.010441306 | 0.20523476 | Up |
| XLOC_004414 | 1.317702194 | 0.010443888 | 0.20523476 | Up |
| OR8S1 | 1.695318363 | 0.010443941 | 0.20523476 | Up |
| USHBP1 | 1.155429293 | 0.010456446 | 0.20523476 | Up |
| MAP2K7 | -1.049603066 | 0.010468535 | 0.20523476 | Down |
| KLHDC8A | 1.010519724 | 0.010479632 | 0.20523476 | Up |
| OSBPL7 | -1.083247885 | 0.010487139 | 0.20523476 | Down |
| XLOC_l2_005517 | -1.153919372 | 0.010488299 | 0.20523476 | Down |
| SLC26A8 | 1.075867444 | 0.010515276 | 0.20523476 | Up |
| HERPUD1 | -1.029520564 | 0.010551785 | 0.20523476 | Down |
| WFDC10B | 1.367915145 | 0.010583617 | 0.20523476 | Up |
| XLOC_006652 | 2.691526226 | 0.010599984 | 0.20523476 | Up |
| ZFYVE1 | -1.147245014 | 0.010617241 | 0.20523476 | Down |
| PCDHGC3 | -1.179227542 | 0.010617753 | 0.20523476 | Down |
| XLOC_004946 | 1.056615283 | 0.010620661 | 0.20523476 | Up |
| PLCL1 | -1.48308357 | 0.010622678 | 0.20523476 | Down |
| DNAJA2 | -1.138836467 | 0.010630758 | 0.20523476 | Down |
| MS4A2 | -1.293875333 | 0.010663017 | 0.20523476 | Down |
| XLOC_006333 | 1.209274525 | 0.010664395 | 0.20523476 | Up |
| SNORD68 | -1.165556962 | 0.010675656 | 0.20523476 | Down |
| OR1D5 | 2.128124479 | 0.010689168 | 0.20523476 | Up |
| XLOC_001699 | 1.33089742 | 0.010696014 | 0.20523476 | Up |
| MGC34034 | 1.143839272 | 0.01070344 | 0.20523476 | Up |
| THY1 | -2.094162083 | 0.010726056 | 0.20523476 | Down |
| MUSK | 1.10428002 | 0.010729253 | 0.20523476 | Up |
| XLOC_005613 | 1.279662492 | 0.010740338 | 0.20523476 | Up |
| XLOC_000352 | 1.054374525 | 0.010741148 | 0.20523476 | Up |
| XLOC_011740 | 1.122814215 | 0.010747268 | 0.20523476 | Up |
| DCP2 | -1.303037722 | 0.010751996 | 0.20523476 | Down |
| GNB2L1 | -1.051876502 | 0.010756702 | 0.20523476 | Down |
| C11orf40 | 1.677833704 | 0.010766233 | 0.20523476 | Up |
| MLL2 | -1.133259325 | 0.010775729 | 0.20523476 | Down |
| XLOC_006597 | 1.581641988 | 0.010788205 | 0.20523476 | Up |
| LIMS2 | -1.355046771 | 0.010791615 | 0.20523476 | Down |
| CRNN | 1.055947185 | 0.010793906 | 0.20523476 | Up |
| C17orf79 | -1.032312236 | 0.010797889 | 0.20523476 | Down |
| MSL3 | -1.02267303 | 0.010816975 | 0.20523476 | Down |
| XLOC_l2_015360 | 1.286874093 | 0.01081795 | 0.20523476 | Up |
| PCNT | -1.096676229 | 0.01082201 | 0.20523476 | Down |
| HERC1 | -1.129322412 | 0.010830453 | 0.20523476 | Down |
| HIST1H4H | -1.13499854 | 0.010831212 | 0.20523476 | Down |
| STAG1 | -1.322673537 | 0.010831266 | 0.20523476 | Down |
| UTP23 | -1.015075092 | 0.010833582 | 0.20523476 | Down |
| PRRX1 | -1.623932814 | 0.01084238 | 0.20523476 | Down |
| OCIAD1 | -1.097297481 | 0.010848305 | 0.20523476 | Down |
| APLP2 | -1.04139062 | 0.010852411 | 0.20523476 | Down |
| XLOC_011235 | 1.45315186 | 0.010864934 | 0.20523476 | Up |
| XLOC_006513 | -1.129508774 | 0.010866344 | 0.20523476 | Down |
| KRTAP4-8 | 1.733474738 | 0.010873698 | 0.20523476 | Up |
| C1S | -1.659848798 | 0.010898316 | 0.20523476 | Down |
| XLOC_006183 | 1.370264539 | 0.010904569 | 0.20523476 | Up |
| DDX58 | -1.281397654 | 0.010905881 | 0.20523476 | Down |
| CCDC3 | -1.701933325 | 0.010907762 | 0.20523476 | Down |
| GXYLT2 | -1.696451149 | 0.010909523 | 0.20523476 | Down |
| OR2J3 | 1.180427465 | 0.010922326 | 0.20523476 | Up |
| ZZZ3 | -1.076007394 | 0.010930951 | 0.20523476 | Down |
| CCNB1IP1 | -1.312165647 | 0.010938599 | 0.20523476 | Down |
| XLOC_013723 | 1.054830647 | 0.010953855 | 0.20523476 | Up |
| RAD21 | -1.234685147 | 0.010971884 | 0.20523476 | Down |
| UCHL1 | -2.343143797 | 0.010972423 | 0.20523476 | Down |
| CYP2U1 | -1.426455267 | 0.010973962 | 0.20523476 | Down |
| XLOC_012763 | 1.504548647 | 0.010989433 | 0.20523476 | Up |
| XLOC_003468 | -1.115018 | 0.010995498 | 0.20523476 | Down |
| RGS22 | -1.003762539 | 0.011003187 | 0.20523476 | Down |
| TRIM72 | 1.086510568 | 0.011005657 | 0.20523476 | Up |
| ADI1 | -1.104977977 | 0.011011158 | 0.20523476 | Down |
| OR6K2 | 1.527915883 | 0.011012221 | 0.20523476 | Up |
| JMJD7-PLA2G4B | -1.241631206 | 0.011013201 | 0.20523476 | Down |
| RAD9A | -1.019413733 | 0.011036229 | 0.20523476 | Down |
| HOXB1 | 1.032420351 | 0.011047827 | 0.20523476 | Up |
| TARS | -1.050777692 | 0.011071575 | 0.20523476 | Down |
| HTR6 | 1.108043298 | 0.01109037 | 0.20523476 | Up |
| CLSTN2 | -2.072988508 | 0.011102231 | 0.20523476 | Down |
| P2RX3 | 1.022318162 | 0.011108744 | 0.20523476 | Up |
| FAM89A | -1.504548665 | 0.011110193 | 0.20523476 | Down |
| XLOC_001302 | 1.112198725 | 0.011123919 | 0.20523476 | Up |
| TPTE2P3 | 1.117368048 | 0.011144564 | 0.20523476 | Up |
| LPA | 1.395917554 | 0.011190977 | 0.20523476 | Up |
| XLOC_l2_004283 | 1.567760934 | 0.011192022 | 0.20523476 | Up |
| XLOC_005297 | 1.139360476 | 0.011199391 | 0.20523476 | Up |
| C15orf57 | -1.074874342 | 0.011206234 | 0.20523476 | Down |
| KIAA0430 | -1.016537388 | 0.011213097 | 0.20523476 | Down |
| ANGPT2 | -1.712344196 | 0.011220758 | 0.20523476 | Down |
| XLOC_004991 | 1.377852853 | 0.011224234 | 0.20523476 | Up |
| SEPT11 | -1.229128826 | 0.011242141 | 0.20523476 | Down |
| XLOC_l2_012661 | 1.888858267 | 0.011251315 | 0.20523476 | Up |
| RPS13 | -1.171149973 | 0.011256553 | 0.20523476 | Down |
| GBF1 | -1.2379411 | 0.01126037 | 0.20523476 | Down |
| NDUFA4 | -1.002050847 | 0.01126532 | 0.20523476 | Down |
| PHOX2B | 1.470050242 | 0.011275033 | 0.20523476 | Up |
| XLOC_009631 | 1.404153921 | 0.01127992 | 0.20523476 | Up |
| XLOC_002969 | 1.826578041 | 0.011284842 | 0.20523476 | Up |
| XLOC_010256 | 1.674652966 | 0.011288402 | 0.20523476 | Up |
| XLOC_005088 | 1.414030468 | 0.011288529 | 0.20523476 | Up |
| XLOC_004525 | 1.633665975 | 0.011295954 | 0.20523476 | Up |
| CLEC12B | 1.411419746 | 0.011301109 | 0.20523476 | Up |
| XLOC_005946 | 1.210542184 | 0.011311683 | 0.20523476 | Up |
| XLOC_000430 | 1.026470228 | 0.011331064 | 0.20523476 | Up |
| XLOC_001726 | 1.332298015 | 0.011348391 | 0.20523476 | Up |
| CCL7 | 1.160975202 | 0.011355238 | 0.20523476 | Up |
| XLOC_013712 | 2.17190717 | 0.011378654 | 0.20523476 | Up |
| EIF3H | -1.127903881 | 0.011385823 | 0.20523476 | Down |
| HJURP | 1.470209738 | 0.011398846 | 0.20523476 | Up |
| CLEC3B | -1.986093702 | 0.011405144 | 0.20523476 | Down |
| XLOC_004418 | 1.181329743 | 0.011419688 | 0.20523476 | Up |
| RNASEH2C | -1.257459446 | 0.01142104 | 0.20523476 | Down |
| XLOC_006952 | 1.943385376 | 0.011427256 | 0.20523476 | Up |
| DAPK3 | -1.197402797 | 0.011441441 | 0.20523476 | Down |
| CA5A | 1.005330489 | 0.011453073 | 0.20523476 | Up |
| RBMY2FP | 1.259275122 | 0.011478283 | 0.20523476 | Up |
| C4orf26 | 1.486634139 | 0.011478366 | 0.20523476 | Up |
| PID1 | -1.343698858 | 0.011532639 | 0.20523476 | Down |
| TERF2IP | -1.145917204 | 0.011542353 | 0.20523476 | Down |
| C16orf62 | -1.224618188 | 0.011560276 | 0.20523476 | Down |
| MMP1 | 1.539611493 | 0.011564184 | 0.20523476 | Up |
| XLOC_004173 | 1.486240024 | 0.011586951 | 0.20523476 | Up |
| XLOC_009499 | 1.037937675 | 0.011592069 | 0.20523476 | Up |
| NT5C3L | -1.380120255 | 0.011595336 | 0.20523476 | Down |
| ITPKB | -1.046833377 | 0.011611919 | 0.20523476 | Down |
| ZSCAN23 | -1.136725783 | 0.011623886 | 0.20523476 | Down |
| ZNF180 | -1.031828703 | 0.011641327 | 0.20523476 | Down |
| RGN | -1.552320241 | 0.011651016 | 0.20523476 | Down |
| XLOC_009907 | 1.10282157 | 0.011659645 | 0.20523476 | Up |
| SNORD115-5 | 2.058888471 | 0.011660579 | 0.20523476 | Up |
| XLOC_011468 | 1.809731911 | 0.011672846 | 0.20523476 | Up |
| FER1L4 | 1.42177061 | 0.011687212 | 0.20523476 | Up |
| ZNF766 | -1.307866763 | 0.011695436 | 0.20523476 | Down |
| XLOC_003736 | 1.364198967 | 0.011697631 | 0.20523476 | Up |
| YIPF7 | 1.2825838 | 0.011713903 | 0.20523476 | Up |
| NEFL | 1.467593804 | 0.011714064 | 0.20523476 | Up |
| BRAT1 | -1.074528429 | 0.011716212 | 0.20523476 | Down |
| ZNF512 | -1.561576208 | 0.011741202 | 0.20523476 | Down |
| PITHD1 | -1.098753672 | 0.011747724 | 0.20523476 | Down |
| XLOC_l2_007059 | 1.71961133 | 0.011767295 | 0.20523476 | Up |
| CMTM3 | -1.147935228 | 0.011772445 | 0.20523476 | Down |
| XLOC_011198 | 1.550742013 | 0.011775909 | 0.20523476 | Up |
| DUSP27 | 1.078117983 | 0.011776721 | 0.20523476 | Up |
| XLOC_l2_010948 | 1.170363805 | 0.011777271 | 0.20523476 | Up |
| CLDND1 | -1.070179221 | 0.011780063 | 0.20523476 | Down |
| XLOC_011978 | 2.442477925 | 0.011797647 | 0.20523476 | Up |
| XLOC_012406 | 1.35707354 | 0.01181288 | 0.20523476 | Up |
| ICK | -1.205553752 | 0.011812894 | 0.20523476 | Down |
| XLOC_006073 | 1.566383514 | 0.011816904 | 0.20523476 | Up |
| HIST1H4F | -1.090458486 | 0.011825474 | 0.20523476 | Down |
| SKI | -1.150878488 | 0.011826212 | 0.20523476 | Down |
| JAM3 | -1.818903599 | 0.011863053 | 0.20523476 | Down |
| XLOC_008136 | -1.076317221 | 0.011863118 | 0.20523476 | Down |
| LIM2 | 1.582239613 | 0.011891474 | 0.20523476 | Up |
| USP9X | -1.229795265 | 0.011912204 | 0.20523476 | Down |
| BCL2L14 | 1.016675786 | 0.011917703 | 0.20523476 | Up |
| RBPMS2 | -1.502331405 | 0.01193294 | 0.20523476 | Down |
| XLOC_004058 | 1.154672159 | 0.011946484 | 0.20523476 | Up |
| KRT16P3 | 1.988318436 | 0.011981832 | 0.20523476 | Up |
| EHBP1 | -1.469800248 | 0.011986505 | 0.20523476 | Down |
| XLOC_002639 | 1.098146859 | 0.011990827 | 0.20523476 | Up |
| XLOC_004413 | 1.805276211 | 0.011992263 | 0.20523476 | Up |
| IQSEC1 | -1.010218182 | 0.011995253 | 0.20523476 | Down |
| XLOC_005462 | 2.067607788 | 0.012003193 | 0.20523476 | Up |
| LHX6 | -1.281496683 | 0.012006849 | 0.20523476 | Down |
| XLOC_011570 | 1.216291373 | 0.012008067 | 0.20523476 | Up |
| TMEM14E | -1.131659932 | 0.012027366 | 0.20523476 | Down |
| DPF3 | 1.669652373 | 0.012027752 | 0.20523476 | Up |
| SLC8A1 | -1.158719415 | 0.012031634 | 0.20523476 | Down |
| XLOC_003239 | 1.365643081 | 0.012032034 | 0.20523476 | Up |
| XLOC_l2_001851 | 1.642763481 | 0.012046536 | 0.20523476 | Up |
| XLOC_013163 | 1.080758647 | 0.012064809 | 0.20523476 | Up |
| XLOC_002729 | 1.446776572 | 0.012066387 | 0.20523476 | Up |
| BHLHB9 | -1.380863482 | 0.012079491 | 0.20523476 | Down |
| TBC1D19 | -1.350809639 | 0.012083078 | 0.20523476 | Down |
| SNORD116-6 | -1.144052636 | 0.012087467 | 0.20523476 | Down |
| BMPR2 | -1.395641143 | 0.012111538 | 0.20523476 | Down |
| FAM71E1 | -1.024008867 | 0.012114268 | 0.20523476 | Down |
| XLOC_012768 | 1.23669937 | 0.012133163 | 0.20523476 | Up |
| XLOC_005599 | 1.383327856 | 0.012140776 | 0.20523476 | Up |
| XLOC_007406 | 1.096729704 | 0.012160135 | 0.20523476 | Up |
| ATXN8OS | 1.252013499 | 0.012167281 | 0.20523476 | Up |
| XLOC_002040 | 1.396114245 | 0.01216783 | 0.20523476 | Up |
| ABHD14B | -1.568373162 | 0.012177946 | 0.20523476 | Down |
| SNORD91B | 1.444853482 | 0.012178605 | 0.20523476 | Up |
| XLOC_l2_007315 | 1.079619498 | 0.012189317 | 0.20523476 | Up |
| LCE6A | 1.543752961 | 0.01222708 | 0.20523476 | Up |
| XLOC_011261 | 1.235096147 | 0.01223694 | 0.20523476 | Up |
| RPS16 | -1.110890534 | 0.012250614 | 0.20523476 | Down |
| GYS2 | 1.100835111 | 0.012254557 | 0.20523476 | Up |
| SLITRK1 | 1.003421036 | 0.012258979 | 0.20523476 | Up |
| XLOC_004775 | 1.037413923 | 0.012267426 | 0.20523476 | Up |
| XLOC_012002 | 1.001123187 | 0.012286608 | 0.20523476 | Up |
| ZNF765 | -1.017036299 | 0.012301151 | 0.20523476 | Down |
| RPL10L | -1.127025356 | 0.012316603 | 0.20523476 | Down |
| XLOC_012949 | 1.44489893 | 0.012317515 | 0.20523476 | Up |
| XLOC_000928 | 1.178265404 | 0.012321642 | 0.20523476 | Up |
| PIP4K2A | -1.033328733 | 0.012334212 | 0.20523476 | Down |
| XLOC_012765 | 2.189418111 | 0.01233615 | 0.20523476 | Up |
| TRAPPC1 | -1.235449689 | 0.012336688 | 0.20523476 | Down |
| XLOC_004254 | 1.32658044 | 0.012337792 | 0.20523476 | Up |
| ZNF654 | -1.392795174 | 0.012352656 | 0.20523476 | Down |
| AP2M1 | -1.031774753 | 0.012353353 | 0.20523476 | Down |
| EIF3K | -1.340148126 | 0.012391456 | 0.20523476 | Down |
| KCTD15 | -1.207521491 | 0.012395517 | 0.20523476 | Down |
| XLOC_l2_010843 | 1.600109117 | 0.012414988 | 0.20523476 | Up |
| XLOC_009294 | 1.621294352 | 0.012421612 | 0.20523476 | Up |
| ESPL1 | 1.218100788 | 0.012432493 | 0.20523476 | Up |
| RPS25 | -1.037609705 | 0.012441647 | 0.20523476 | Down |
| CYLD | -1.105200475 | 0.01244378 | 0.20523476 | Down |
| TCEAL4 | -1.287916829 | 0.01247044 | 0.20523476 | Down |
| CTRC | 1.345142374 | 0.012487855 | 0.20523476 | Up |
| RBP1 | -1.755680682 | 0.012492697 | 0.20523476 | Down |
| NPTN | -1.043449674 | 0.012495133 | 0.20523476 | Down |
| HIST1H4I | -1.116857366 | 0.012537439 | 0.20523476 | Down |
| XLOC_006315 | 1.286313045 | 0.012539767 | 0.20523476 | Up |
| XLOC_001426 | 1.119799499 | 0.012544863 | 0.20523476 | Up |
| ZFYVE20 | -1.257201445 | 0.012545083 | 0.20523476 | Down |
| C7orf62 | 1.312073631 | 0.012583607 | 0.20523476 | Up |
| XLOC_006164 | 1.583714272 | 0.012586083 | 0.20523476 | Up |
| XLOC_l2_013513 | 1.419639031 | 0.012602438 | 0.20523476 | Up |
| XLOC_l2_005759 | 1.080449102 | 0.012602604 | 0.20523476 | Up |
| XLOC_004951 | 1.203477194 | 0.012628345 | 0.20523476 | Up |
| DCN | -1.893270877 | 0.012631152 | 0.20523476 | Down |
| TAF1C | -1.007429678 | 0.012643177 | 0.20523476 | Down |
| XLOC_005521 | 1.001502631 | 0.012658836 | 0.20523476 | Up |
| XLOC_000733 | 1.386120305 | 0.012660472 | 0.20523476 | Up |
| PDE5A | -1.176565048 | 0.01266953 | 0.20523476 | Down |
| XLOC_007928 | 1.13437818 | 0.012679689 | 0.20523476 | Up |
| DMPK | -1.201514387 | 0.012694491 | 0.20523476 | Down |
| XLOC_003757 | 1.08473666 | 0.012695128 | 0.20523476 | Up |
| SPON1 | -1.926282986 | 0.012709259 | 0.20523476 | Down |
| XLOC_l2_005403 | 2.227277998 | 0.012718286 | 0.20523476 | Up |
| XLOC_l2_014182 | 1.732114284 | 0.012725044 | 0.20523476 | Up |
| XLOC_009535 | 1.096904187 | 0.012726325 | 0.20523476 | Up |
| XLOC_l2_015209 | 1.493015729 | 0.012726483 | 0.20523476 | Up |
| DDX39A | -1.111720159 | 0.012730376 | 0.20523476 | Down |
| KLC1 | -1.068697472 | 0.012745042 | 0.20523476 | Down |
| VEGFB | -1.249970524 | 0.012751332 | 0.20523476 | Down |
| PDXDC1 | -1.036617414 | 0.012761625 | 0.20523476 | Down |
| GBAS | -1.008856369 | 0.012762596 | 0.20523476 | Down |
| SNORD115-28 | 1.576942752 | 0.012764232 | 0.20523476 | Up |
| OR52I2 | 1.141228047 | 0.012770989 | 0.20523476 | Up |
| TBC1D23 | -1.063427733 | 0.012774535 | 0.20523476 | Down |
| ABL1 | -1.266416995 | 0.012788573 | 0.20523476 | Down |
| LIFR | -1.60868276 | 0.012792866 | 0.20523476 | Down |
| TTC9B | 1.024740338 | 0.012805056 | 0.20523476 | Up |
| XLOC_003289 | 1.686279963 | 0.012811505 | 0.20523476 | Up |
| XLOC_004969 | 1.052955807 | 0.012819042 | 0.20523476 | Up |
| XLOC_004410 | 1.464661293 | 0.01282985 | 0.20523476 | Up |
| XLOC_004777 | 1.018851957 | 0.012837251 | 0.20523476 | Up |
| MYRIP | -1.341554494 | 0.012840796 | 0.20523476 | Down |
| KLHL5 | -1.269405102 | 0.012869613 | 0.20523476 | Down |
| SARS2 | -1.02259328 | 0.012880275 | 0.20523476 | Down |
| ATP8A1 | -1.077597641 | 0.012881183 | 0.20523476 | Down |
| MAN1C1 | -1.177904193 | 0.012883632 | 0.20523476 | Down |
| GOT2 | -1.021892207 | 0.012890894 | 0.20523476 | Down |
| XLOC_005182 | 2.689317678 | 0.012906066 | 0.20523476 | Up |
| MS4A15 | 1.110996277 | 0.0129081 | 0.20523476 | Up |
| XLOC_009724 | -1.136833758 | 0.01291194 | 0.20523476 | Down |
| FABP3 | -1.247452368 | 0.012919308 | 0.20523476 | Down |
| XLOC_007867 | 1.393539585 | 0.012925512 | 0.20523476 | Up |
| C1orf200 | 1.194716932 | 0.012925779 | 0.20523476 | Up |
| XLOC_010743 | 1.402970992 | 0.012935115 | 0.20523476 | Up |
| LDOC1 | -1.27426306 | 0.01295646 | 0.20523476 | Down |
| ANKRD20A3 | 1.584585119 | 0.012957842 | 0.20523476 | Up |
| CKMT2 | -1.341274137 | 0.012965928 | 0.20523476 | Down |
| XLOC_l2_010601 | 1.140595489 | 0.01296985 | 0.20523476 | Up |
| CD248 | -1.705326594 | 0.012972997 | 0.20523476 | Down |
| XLOC_002927 | 1.583810704 | 0.012983365 | 0.20523476 | Up |
| HMGB3 | 1.123442254 | 0.012988872 | 0.20523476 | Up |
| VEGFC | -1.558872934 | 0.012996544 | 0.20523476 | Down |
| XLOC_011089 | 1.115157147 | 0.012997075 | 0.20523476 | Up |
| SNX1 | -1.273553775 | 0.013000539 | 0.20523476 | Down |
| WWTR1 | -1.170173315 | 0.013026011 | 0.20523476 | Down |
| LINC00460 | 1.798491745 | 0.013039981 | 0.20523476 | Up |
| XLOC_014255 | 1.117005826 | 0.01304908 | 0.20523476 | Up |
| XLOC_005085 | 1.643732294 | 0.013061206 | 0.20523476 | Up |
| TMEM80 | -1.399254088 | 0.01306953 | 0.20523476 | Down |
| PEPD | -1.032851526 | 0.013087405 | 0.20523476 | Down |
| ANKDD1B | 1.045199012 | 0.013103775 | 0.20523476 | Up |
| FOXJ3 | -1.086701669 | 0.013109522 | 0.20523476 | Down |
| XLOC_010214 | 1.310308341 | 0.013118363 | 0.20523476 | Up |
| THEG | 1.052252553 | 0.013193873 | 0.20523476 | Up |
| XLOC_011722 | 1.141613135 | 0.013197876 | 0.20523476 | Up |
| CCDC97 | -1.023812612 | 0.013198909 | 0.20523476 | Down |
| RSPH6A | 1.462756802 | 0.013199886 | 0.20523476 | Up |
| IL37 | 1.278287354 | 0.013208975 | 0.20523476 | Up |
| NUCB2 | -1.070966707 | 0.013209819 | 0.20523476 | Down |
| TPST1 | -1.284620279 | 0.013238379 | 0.20523476 | Down |
| XLOC_010576 | 1.364764174 | 0.013239908 | 0.20523476 | Up |
| SAMD4A | -1.06550277 | 0.013254592 | 0.20523476 | Down |
| VGLL4 | -1.174469915 | 0.013261935 | 0.20523476 | Down |
| EMP3 | -1.177039414 | 0.013271398 | 0.20523476 | Down |
| XLOC_001745 | 1.461091675 | 0.013280619 | 0.20523476 | Up |
| XLOC_004439 | 1.291284989 | 0.013282812 | 0.20523476 | Up |
| XLOC_010356 | 1.140507884 | 0.013288155 | 0.20523476 | Up |
| XLOC_009798 | 1.84651328 | 0.01332052 | 0.20523476 | Up |
| XLOC_004774 | 1.367147414 | 0.013340027 | 0.20523476 | Up |
| XLOC_013573 | 1.059189458 | 0.013353723 | 0.20523476 | Up |
| TCEAL1 | -1.224814265 | 0.013394758 | 0.20523476 | Down |
| ABCG1 | -1.100471353 | 0.013403733 | 0.20523476 | Down |
| ACTL8 | 1.440464569 | 0.01340848 | 0.20523476 | Up |
| XLOC_l2_006037 | 1.526652108 | 0.013428101 | 0.20523476 | Up |
| XLOC_004376 | 1.014042608 | 0.013433108 | 0.20523476 | Up |
| XLOC_011636 | 1.469554515 | 0.013443471 | 0.20523476 | Up |
| XLOC_007358 | 1.685719175 | 0.013451479 | 0.20523476 | Up |
| FAM71F1 | 1.209314048 | 0.013454243 | 0.20523476 | Up |
| NCAPD2 | -1.040803613 | 0.013466163 | 0.20523476 | Down |
| ARHGEF17 | -1.456600111 | 0.013482488 | 0.20523476 | Down |
| XLOC_006929 | 1.21746813 | 0.013482951 | 0.20523476 | Up |
| XLOC_007437 | 1.101162756 | 0.013501779 | 0.20523476 | Up |
| KRTAP11-1 | 1.192624428 | 0.013509306 | 0.20523476 | Up |
| NGRN | -1.060038304 | 0.013532808 | 0.20523476 | Down |
| TAF7 | -1.014395088 | 0.013537528 | 0.20523476 | Down |
| MRPS9 | -1.005119365 | 0.013538563 | 0.20523476 | Down |
| SLC14A1 | -1.477428851 | 0.013539382 | 0.20523476 | Down |
| ABCC9 | -1.836213224 | 0.013560577 | 0.20523476 | Down |
| TGDS | -1.069025491 | 0.013568671 | 0.20523476 | Down |
| RCAN2 | -1.33196963 | 0.013575353 | 0.20523476 | Down |
| MFAP3L | -1.193768469 | 0.013576005 | 0.20523476 | Down |
| XLOC_001882 | 1.757354939 | 0.013578878 | 0.20523476 | Up |
| XLOC_l2_014086 | 1.019904138 | 0.013578904 | 0.20523476 | Up |
| C7orf55 | -1.260810085 | 0.013583264 | 0.20523476 | Down |
| XLOC_001258 | 1.181980604 | 0.013598212 | 0.20523476 | Up |
| DNAPTP3 | -2.055578463 | 0.013625798 | 0.20523476 | Down |
| SLBP | -1.096421348 | 0.013648488 | 0.20523476 | Down |
| RAB33B | -1.320140491 | 0.0136543 | 0.20523476 | Down |
| GDPD4 | 1.030373807 | 0.013682275 | 0.20523476 | Up |
| PSMD7 | -1.021434638 | 0.013682319 | 0.20523476 | Down |
| COA5 | -1.161245568 | 0.013692991 | 0.20523476 | Down |
| FKBP15 | -1.092282162 | 0.013700498 | 0.20523476 | Down |
| O3FAR1 | 1.257385451 | 0.013705559 | 0.20523476 | Up |
| HIST1H4E | -1.130810003 | 0.013709334 | 0.20523476 | Down |
| ZNF804A | 1.507138458 | 0.013715666 | 0.20523476 | Up |
| OBP2A | 1.244338008 | 0.013725846 | 0.20523476 | Up |
| LAMC3 | -1.466822665 | 0.013738125 | 0.20523476 | Down |
| OR5D16 | 1.541740451 | 0.013750702 | 0.20523476 | Up |
| XLOC_l2_011891 | 1.434749353 | 0.01376198 | 0.20523476 | Up |
| XLOC_010839 | 1.417265756 | 0.013763045 | 0.20523476 | Up |
| C17orf90 | -1.097238179 | 0.013768491 | 0.20523476 | Down |
| CAPN11 | 1.148386838 | 0.013769049 | 0.20523476 | Up |
| XLOC_l2_014011 | 1.461606121 | 0.0137694 | 0.20523476 | Up |
| XLOC_003422 | 1.28568169 | 0.013782088 | 0.20523476 | Up |
| MED21 | -1.053183388 | 0.013782937 | 0.20523476 | Down |
| SELM | -1.625276385 | 0.013810521 | 0.20523476 | Down |
| TMEM109 | -1.278070886 | 0.013814292 | 0.20523476 | Down |
| C16orf88 | -1.02182614 | 0.013842471 | 0.20523476 | Down |
| RPL15 | -1.043621772 | 0.013845052 | 0.20523476 | Down |
| RAMP1 | -1.74052955 | 0.013870963 | 0.20523476 | Down |
| SPANXN5 | 1.237743222 | 0.013870976 | 0.20523476 | Up |
| FGF13 | -1.894231329 | 0.01387383 | 0.20523476 | Down |
| C17orf99 | 1.907551128 | 0.013878029 | 0.20523476 | Up |
| CCDC14 | -1.199106115 | 0.01389131 | 0.20523476 | Down |
| C13orf41 | 1.29879804 | 0.013892854 | 0.20523476 | Up |
| RABGAP1L | -1.096151637 | 0.013908417 | 0.20523476 | Down |
| DPPA2 | 1.21586683 | 0.013937013 | 0.20523476 | Up |
| GLTSCR2 | -1.23069091 | 0.013938705 | 0.20523476 | Down |
| OR2W5 | 1.429920701 | 0.013942867 | 0.20523476 | Up |
| HEYL | -1.816956996 | 0.013952889 | 0.20523476 | Down |
| MAGEB1 | 1.295559867 | 0.013963875 | 0.20523476 | Up |
| XLOC_006559 | 1.292490303 | 0.013984004 | 0.20523476 | Up |
| ATP6V1B1 | -1.106810718 | 0.013998035 | 0.20523476 | Down |
| SEPP1 | -1.780522356 | 0.014019745 | 0.20523476 | Down |
| XLOC_011973 | 1.401940817 | 0.014021785 | 0.20523476 | Up |
| FAM54A | 1.082348058 | 0.014027906 | 0.20523476 | Up |
| XLOC_011979 | 1.897718396 | 0.014036541 | 0.20523476 | Up |
| PIPOX | 1.477195031 | 0.014041044 | 0.20523476 | Up |
| NFASC | -1.178034754 | 0.014051779 | 0.20523476 | Down |
| SMTNL2 | -1.716218313 | 0.01405565 | 0.20523476 | Down |
| PWP1 | -1.01431246 | 0.014079233 | 0.20523476 | Down |
| VPS13B | -1.192832832 | 0.014091314 | 0.20523476 | Down |
| ZNF227 | -1.172430457 | 0.014091968 | 0.20523476 | Down |
| TH1L | -1.054699534 | 0.014099963 | 0.20523476 | Down |
| IPO8 | -1.128540214 | 0.014105643 | 0.20523476 | Down |
| XLOC_011056 | 1.688005979 | 0.014134666 | 0.20523476 | Up |
| OR4D6 | 1.062119393 | 0.014139614 | 0.20523476 | Up |
| XLOC_l2_005781 | 1.147776337 | 0.014144491 | 0.20523476 | Up |
| XLOC_012857 | 1.265936629 | 0.0141464 | 0.20523476 | Up |
| XLOC_006620 | 1.170783358 | 0.014162586 | 0.20523476 | Up |
| TNNI3 | 1.747263409 | 0.014170642 | 0.20523476 | Up |
| XLOC_000767 | 2.494500857 | 0.014187257 | 0.20523476 | Up |
| GLRA1 | 1.165831621 | 0.014208079 | 0.20523476 | Up |
| ZFP36L1 | -1.039088831 | 0.014209311 | 0.20523476 | Down |
| NUB1 | -1.16076752 | 0.01420952 | 0.20523476 | Down |
| ACRV1 | 1.113467958 | 0.014213447 | 0.20523476 | Up |
| XLOC_002514 | 1.334576234 | 0.014222856 | 0.20523476 | Up |
| CNOT8 | -1.080886117 | 0.014225613 | 0.20523476 | Down |
| MUC3A | 1.720649108 | 0.014226 | 0.20523476 | Up |
| XLOC_002134 | 1.094935713 | 0.014263655 | 0.20523476 | Up |
| NUMBL | -1.260918642 | 0.014267326 | 0.20523476 | Down |
| IGIP | -1.506679337 | 0.014268222 | 0.20523476 | Down |
| XLOC_000927 | 1.21547576 | 0.014277007 | 0.20523476 | Up |
| XLOC_010084 | 1.79867544 | 0.014277422 | 0.20523476 | Up |
| MAP2K5 | -1.379382722 | 0.014299029 | 0.20523476 | Down |
| XLOC_005876 | 1.05805451 | 0.014315127 | 0.20523476 | Up |
| XLOC_l2_013729 | 1.038265899 | 0.014316263 | 0.20523476 | Up |
| OR2L2 | 1.013424291 | 0.014317441 | 0.20523476 | Up |
| XLOC_009215 | 1.165523046 | 0.014321182 | 0.20523476 | Up |
| OR2L8 | 2.404801806 | 0.014344365 | 0.205237898 | Up |
| XLOC_004514 | 1.124867945 | 0.014363277 | 0.205237898 | Up |
| CPS1 | -1.469668688 | 0.014374238 | 0.205237898 | Down |
| TGFB2 | -1.586837518 | 0.014374279 | 0.205237898 | Down |
| CLIP3 | -2.100914878 | 0.014380863 | 0.205237898 | Down |
| ERI3 | -1.083306606 | 0.014390485 | 0.205237898 | Down |
| XLOC_005820 | 1.678412133 | 0.014403094 | 0.205237898 | Up |
| PSG10P | 1.526903854 | 0.014406287 | 0.205237898 | Up |
| XLOC_010490 | 1.60913883 | 0.014413662 | 0.205237898 | Up |
| UBTF | -1.230212902 | 0.014447094 | 0.205237898 | Down |
| DERA | -1.054398689 | 0.01447378 | 0.205237898 | Down |
| XLOC_004350 | 1.305350158 | 0.014480275 | 0.205237898 | Up |
| TPRG1L | -1.075209243 | 0.01448416 | 0.205237898 | Down |
| XLOC_001816 | 1.118287636 | 0.014487589 | 0.205237898 | Up |
| OR9G9 | 1.779188107 | 0.014489804 | 0.205237898 | Up |
| XLOC_l2_011694 | 1.066050944 | 0.014494421 | 0.205237898 | Up |
| CRADD | -1.126934906 | 0.014495168 | 0.205237898 | Down |
| XLOC_007540 | 1.419707874 | 0.014512232 | 0.205237898 | Up |
| LGSN | 1.189373757 | 0.014518531 | 0.205237898 | Up |
| XLOC_l2_013311 | 1.45266162 | 0.014526498 | 0.205237898 | Up |
| XLOC_007051 | 1.426157006 | 0.014530101 | 0.205237898 | Up |
| PAWR | -1.029610009 | 0.014534453 | 0.205237898 | Down |
| GP1BA | 1.254027906 | 0.014542903 | 0.205237898 | Up |
| XLOC_l2_005187 | 1.344630454 | 0.014571529 | 0.205237898 | Up |
| TIGD2 | -1.164420884 | 0.014583391 | 0.205237898 | Down |
| HIST2H2AB | -1.120124714 | 0.014585823 | 0.205237898 | Down |
| HIST1H2BG | -1.236072738 | 0.014630047 | 0.205237898 | Down |
| XLOC_000223 | 1.067848546 | 0.014662291 | 0.205237898 | Up |
| C5orf50 | 1.134545065 | 0.014668751 | 0.205237898 | Up |
| NEK5 | 1.224673594 | 0.014685036 | 0.205237898 | Up |
| XLOC_002663 | 1.842465732 | 0.014693937 | 0.205237898 | Up |
| XLOC_009345 | 1.320314768 | 0.014716371 | 0.205237898 | Up |
| ALKBH7 | -1.085264908 | 0.0147321 | 0.205237898 | Down |
| GPR114 | 1.341584458 | 0.014752022 | 0.205237898 | Up |
| CILP | -1.488920846 | 0.014752788 | 0.205237898 | Down |
| TAS2R9 | 1.1258186 | 0.014755588 | 0.205237898 | Up |
| XLOC_009550 | 1.622473766 | 0.014761317 | 0.205237898 | Up |
| ZNF844 | -1.156318948 | 0.014775087 | 0.205237898 | Down |
| NENF | -1.031524513 | 0.014780296 | 0.205237898 | Down |
| RAI14 | -1.086630622 | 0.01478048 | 0.205237898 | Down |
| PPIL3 | -1.302932389 | 0.014830635 | 0.205237898 | Down |
| SOX10 | 1.189263293 | 0.014835837 | 0.205237898 | Up |
| XLOC_l2_004327 | 2.228999391 | 0.014854741 | 0.205237898 | Up |
| LRRC37BP1 | 1.626942398 | 0.014857028 | 0.205237898 | Up |
| XLOC_005595 | 1.804751767 | 0.014867662 | 0.205237898 | Up |
| TPH1 | 1.09547543 | 0.01487258 | 0.205237898 | Up |
| PAX9 | 1.417356879 | 0.014877869 | 0.205237898 | Up |
| XLOC_001657 | 1.17682698 | 0.014882591 | 0.205237898 | Up |
| XLOC_001160 | 1.058325051 | 0.014915335 | 0.205237898 | Up |
| PSG5 | 2.638840269 | 0.01491801 | 0.205237898 | Up |
| PARP2 | -1.317158957 | 0.014967676 | 0.205237898 | Down |
| MGLL | -1.19451369 | 0.015008342 | 0.205237898 | Down |
| FES | -1.022935912 | 0.015018109 | 0.205237898 | Down |
| CXorf48 | 1.783624132 | 0.015028984 | 0.205237898 | Up |
| XLOC_010467 | 1.651139671 | 0.015036811 | 0.205237898 | Up |
| XLOC_012318 | 1.204711717 | 0.015037329 | 0.205237898 | Up |
| XLOC_l2_006256 | 1.052776987 | 0.015053378 | 0.205237898 | Up |
| XLOC_011930 | 1.120073316 | 0.015080814 | 0.205237898 | Up |
| XLOC_005955 | 1.475727204 | 0.015100613 | 0.205237898 | Up |
| KCTD6 | -1.128582668 | 0.015103954 | 0.205237898 | Down |
| NSMCE2 | -1.129195152 | 0.015110044 | 0.205237898 | Down |
| XLOC_006226 | 1.69135635 | 0.01512531 | 0.205237898 | Up |
| XLOC_002524 | 1.059553646 | 0.015126805 | 0.205237898 | Up |
| XLOC_000350 | -1.135005517 | 0.015143139 | 0.205237898 | Down |
| XLOC_000095 | 1.268502596 | 0.015145057 | 0.205237898 | Up |
| ABCB1 | -1.176611722 | 0.015145057 | 0.205237898 | Down |
| XLOC_l2_006267 | 1.372404793 | 0.015162572 | 0.205237898 | Up |
| ATXN3 | -1.233692587 | 0.01516716 | 0.205237898 | Down |
| XLOC_000877 | 1.020575751 | 0.015191621 | 0.205237898 | Up |
| XLOC_006176 | 1.797982587 | 0.015202523 | 0.205237898 | Up |
| ZBED5 | -1.000871705 | 0.015209369 | 0.205237898 | Down |
| XLOC_010668 | 1.350084169 | 0.01522205 | 0.205237898 | Up |
| USP28 | -1.261441898 | 0.015228045 | 0.205237898 | Down |
| HIST1H2BM | -1.081444884 | 0.015265262 | 0.205237898 | Down |
| XLOC_004896 | 1.185630807 | 0.015277175 | 0.205237898 | Up |
| FLYWCH2 | -1.283212707 | 0.015277412 | 0.205237898 | Down |
| PYGO2 | -1.032347461 | 0.015280578 | 0.205237898 | Down |
| NHLH1 | 1.267670481 | 0.015315184 | 0.205237898 | Up |
| TMSB15B | -1.379377304 | 0.015322416 | 0.205237898 | Down |
| XLOC_011152 | 1.053723736 | 0.015335827 | 0.205237898 | Up |
| CSTF2T | -1.34955778 | 0.015341282 | 0.205237898 | Down |
| XLOC_008583 | -1.071019878 | 0.015362629 | 0.205237898 | Down |
| FZD4 | -1.192016636 | 0.015369047 | 0.205237898 | Down |
| WEE2 | 1.11914674 | 0.015379429 | 0.205237898 | Up |
| SNORD115-2 | 1.508522459 | 0.015384833 | 0.205237898 | Up |
| NAP1L5 | -1.577128155 | 0.015398673 | 0.205237898 | Down |
| EIF4E3 | -1.009193356 | 0.015434826 | 0.205237898 | Down |
| DDX19A | -1.074554614 | 0.015459722 | 0.205237898 | Down |
| XLOC_l2_014372 | 1.747195847 | 0.015496544 | 0.205237898 | Up |
| COPG2 | -1.164796828 | 0.015509362 | 0.205237898 | Down |
| MDM4 | -1.29148714 | 0.015515897 | 0.205237898 | Down |
| XLOC_000956 | 1.250757651 | 0.015519497 | 0.205237898 | Up |
| NSMAF | -1.244429439 | 0.015520642 | 0.205237898 | Down |
| PLCXD3 | -2.31039786 | 0.01552286 | 0.205237898 | Down |
| XLOC_011080 | 1.271221427 | 0.015524595 | 0.205237898 | Up |
| RPS7 | -1.093255792 | 0.015552011 | 0.205237898 | Down |
| KLHDC2 | -1.180997467 | 0.015576785 | 0.205237898 | Down |
| XLOC_002770 | -1.582177247 | 0.015585237 | 0.205237898 | Down |
| PLA2G4A | -1.062232282 | 0.015594167 | 0.205237898 | Down |
| XLOC_003940 | 1.359289667 | 0.015602639 | 0.205237898 | Up |
| SLC41A1 | -1.137430438 | 0.015605981 | 0.205237898 | Down |
| XLOC_010843 | 1.406263465 | 0.015607753 | 0.205237898 | Up |
| XLOC_007496 | 1.451636494 | 0.0156193 | 0.205237898 | Up |
| RPL7A | -1.154957671 | 0.015634862 | 0.205237898 | Down |
| XLOC_011316 | 2.655666424 | 0.015639262 | 0.205237898 | Up |
| XLOC_l2_000945 | 1.067072675 | 0.015641809 | 0.205237898 | Up |
| XLOC_003737 | 1.080587957 | 0.015666272 | 0.205237898 | Up |
| XLOC_004375 | 1.137044067 | 0.015668146 | 0.205237898 | Up |
| CDKN2C | -1.288064448 | 0.015675899 | 0.205237898 | Down |
| DDHD2 | -1.118295054 | 0.015681825 | 0.205237898 | Down |
| TARSL2 | -1.319677398 | 0.01568647 | 0.205237898 | Down |
| XLOC_000111 | 1.509787647 | 0.015696851 | 0.205237898 | Up |
| SRCIN1 | 1.179092149 | 0.015700474 | 0.205237898 | Up |
| XLOC_008765 | 1.319037187 | 0.015718255 | 0.205237898 | Up |
| XLOC_011786 | 1.705103824 | 0.0157216 | 0.205237898 | Up |
| XLOC_l2_011969 | 1.372414176 | 0.015728274 | 0.205237898 | Up |
| HIST1H2BK | -1.085293225 | 0.015732904 | 0.205237898 | Down |
| XLOC_l2_003414 | 1.158618012 | 0.015733984 | 0.205237898 | Up |
| XLOC_001329 | 1.03049157 | 0.015735221 | 0.205237898 | Up |
| MUC7 | 1.317288724 | 0.015769725 | 0.205237898 | Up |
| MS4A18 | 1.91165371 | 0.015778722 | 0.205237898 | Up |
| XLOC_011433 | 1.473065715 | 0.015780331 | 0.205237898 | Up |
| PRAMEF2 | 1.128657189 | 0.015783578 | 0.205237898 | Up |
| SGIP1 | -1.256265389 | 0.015796991 | 0.205237898 | Down |
| XLOC_003166 | 1.106240333 | 0.015798317 | 0.205237898 | Up |
| XLOC_008062 | 1.887019757 | 0.015831543 | 0.205237898 | Up |
| PPP3CB | -1.087102361 | 0.015831713 | 0.205237898 | Down |
| LINC00028 | 1.016341019 | 0.015841914 | 0.205237898 | Up |
| SEC61B | -1.176032113 | 0.015852712 | 0.205237898 | Down |
| XLOC_005471 | 1.102505268 | 0.01589309 | 0.205237898 | Up |
| XLOC_012072 | 2.170796471 | 0.015916973 | 0.205237898 | Up |
| ESYT2 | -1.231246327 | 0.015918482 | 0.205237898 | Down |
| LYZL1 | 1.130661467 | 0.015919477 | 0.205237898 | Up |
| XLOC_l2_008802 | 1.177231339 | 0.015932581 | 0.205237898 | Up |
| RPL27 | -1.099749168 | 0.015945497 | 0.205237898 | Down |
| XLOC_008427 | 1.897289226 | 0.015951866 | 0.205237898 | Up |
| XLOC_l2_013189 | 1.346488042 | 0.015961527 | 0.205237898 | Up |
| RPL39 | -1.053353211 | 0.015981008 | 0.205237898 | Down |
| XLOC_011725 | 1.749753496 | 0.016002444 | 0.205237898 | Up |
| IRF2BP2 | -1.141179 | 0.016012613 | 0.205237898 | Down |
| BIRC6 | -1.151016466 | 0.016037921 | 0.205237898 | Down |
| PNMA1 | -1.068294108 | 0.016040772 | 0.205237898 | Down |
| XLOC_l2_009468 | 1.146084156 | 0.016042692 | 0.205237898 | Up |
| RPS3A | -1.192106047 | 0.016049857 | 0.205237898 | Down |
| XLOC_010296 | 1.283430089 | 0.01605139 | 0.205237898 | Up |
| HSPA12A | -1.357314212 | 0.01605344 | 0.205237898 | Down |
| XLOC_005807 | 1.267850511 | 0.016073588 | 0.205237898 | Up |
| SUCLG1 | -1.201219703 | 0.016086948 | 0.205237898 | Down |
| PPP1R3B | -1.294533355 | 0.016086996 | 0.205237898 | Down |
| XLOC_004078 | 1.161982099 | 0.016101024 | 0.205237898 | Up |
| XLOC_003733 | 1.108604127 | 0.016101806 | 0.205237898 | Up |
| KIAA1328 | -1.183710391 | 0.016119274 | 0.205237898 | Down |
| XLOC_012884 | 1.064995555 | 0.016133457 | 0.205237898 | Up |
| XLOC_012633 | 1.135342378 | 0.016137498 | 0.205237898 | Up |
| XLOC_003934 | 1.832712415 | 0.016140274 | 0.205237898 | Up |
| OR14I1 | 1.17746704 | 0.016152808 | 0.205237898 | Up |
| BBS7 | -1.117430576 | 0.016169601 | 0.205237898 | Down |
| RNPC3 | -1.23577632 | 0.016170384 | 0.205237898 | Down |
| HDHD2 | -1.066900304 | 0.016178548 | 0.205237898 | Down |
| DEFA9P | 1.099849534 | 0.016184217 | 0.205237898 | Up |
| PFDN4 | -1.016775587 | 0.016198247 | 0.205237898 | Down |
| FLJ31713 | 1.308492001 | 0.01620163 | 0.205237898 | Up |
| XLOC_l2_015196 | 1.168785101 | 0.016206188 | 0.205237898 | Up |
| PLA2G1B | 1.594737243 | 0.01622225 | 0.205237898 | Up |
| TRIM49L2 | 2.049137726 | 0.016227926 | 0.205237898 | Up |
| BAI3 | -1.244583931 | 0.016252452 | 0.205237898 | Down |
| PRKAA1 | -1.049823095 | 0.016276855 | 0.205237898 | Down |
| TMOD1 | -1.305422279 | 0.016277445 | 0.205237898 | Down |
| COQ10A | -1.020128239 | 0.016294124 | 0.205237898 | Down |
| RNF135 | -1.507605093 | 0.016299391 | 0.205237898 | Down |
| OR5M1 | -1.589837144 | 0.01630918 | 0.205237898 | Down |
| RPL9 | -1.286485057 | 0.016333906 | 0.205237898 | Down |
| XLOC_005731 | 1.973483677 | 0.016349243 | 0.205237898 | Up |
| RNASEL | -1.024984747 | 0.016350394 | 0.205237898 | Down |
| XLOC_l2_006797 | 1.07807422 | 0.016364821 | 0.205237898 | Up |
| AOC3 | -1.675017501 | 0.016391929 | 0.205237898 | Down |
| RSPRY1 | -1.083315457 | 0.016404641 | 0.205237898 | Down |
| XLOC_005244 | -1.15354933 | 0.01641618 | 0.205237898 | Down |
| TTC19 | -1.082590858 | 0.016436404 | 0.205237898 | Down |
| GPX8 | -1.357545153 | 0.016447825 | 0.205237898 | Down |
| XLOC_005859 | 1.153740982 | 0.016456769 | 0.205237898 | Up |
| ZNF204P | -1.264604594 | 0.01651362 | 0.205237898 | Down |
| RPL32 | -1.148022881 | 0.016519809 | 0.205237898 | Down |
| APBA1 | -1.473666054 | 0.01656106 | 0.205237898 | Down |
| PISRT1 | 1.16192514 | 0.016584013 | 0.205237898 | Up |
| FRZB | -1.76525678 | 0.016587097 | 0.205237898 | Down |
| XLOC_l2_015762 | -1.128713102 | 0.016589912 | 0.205237898 | Down |
| ADAM22 | -1.247613331 | 0.016602098 | 0.205237898 | Down |
| OLFM1 | 1.080085746 | 0.016607459 | 0.205237898 | Up |
| LHFPL2 | -1.035471795 | 0.016615898 | 0.205237898 | Down |
| C20orf7 | -1.008185296 | 0.016629463 | 0.205237898 | Down |
| XLOC_003458 | 1.072260299 | 0.016646973 | 0.205237898 | Up |
| GPR98 | -1.272876904 | 0.016699733 | 0.205237898 | Down |
| ALDH3A2 | -1.057777022 | 0.016710552 | 0.205237898 | Down |
| KCNE4 | -1.309006532 | 0.016733793 | 0.205237898 | Down |
| XLOC_011313 | 1.028427436 | 0.01673382 | 0.205237898 | Up |
| XLOC_004528 | 1.355981086 | 0.016736218 | 0.205237898 | Up |
| RBFOX2 | -1.035887858 | 0.016756384 | 0.205237898 | Down |
| XLOC_002059 | 1.26204559 | 0.016775999 | 0.205237898 | Up |
| IGFBP7 | -1.525472828 | 0.016788178 | 0.205237898 | Down |
| RPS27A | -1.000497938 | 0.016802071 | 0.205237898 | Down |
| POLQ | 1.108715492 | 0.016814859 | 0.205237898 | Up |
| KAT2A | -1.058648244 | 0.016877636 | 0.205326198 | Down |
| CTSF | -1.041086834 | 0.016900251 | 0.205326198 | Down |
| KIR2DS2 | 2.88484225 | 0.01690123 | 0.205326198 | Up |
| VCAN | -1.660181886 | 0.016929193 | 0.205326198 | Down |
| TTTY19 | 1.567793053 | 0.01693437 | 0.205326198 | Up |
| XLOC_007349 | 1.955233495 | 0.016961835 | 0.205326198 | Up |
| TSSK6 | 1.374901637 | 0.016962576 | 0.205326198 | Up |
| SLC25A36 | -1.05509718 | 0.016977768 | 0.205326198 | Down |
| FAM74A3 | 1.504061669 | 0.016980855 | 0.205326198 | Up |
| PXT1 | 1.420394025 | 0.01700703 | 0.205326198 | Up |
| XLOC_006774 | 1.233501591 | 0.017024629 | 0.205326198 | Up |
| SLC29A4 | 1.152283163 | 0.017026012 | 0.205326198 | Up |
| ARHGEF10 | -1.141660171 | 0.017026106 | 0.205326198 | Down |
| XLOC_011006 | -1.015969211 | 0.01703184 | 0.205326198 | Down |
| CREBL2 | -1.099067612 | 0.017047943 | 0.205326198 | Down |
| XLOC_000230 | 1.193819156 | 0.017048858 | 0.205326198 | Up |
| ACCN1 | 1.00507861 | 0.017050199 | 0.205326198 | Up |
| PAPLN | 1.04798181 | 0.017050389 | 0.205326198 | Up |
| XLOC_005918 | 1.351397734 | 0.017051674 | 0.205326198 | Up |
| ERVV-2 | 1.639234747 | 0.017063152 | 0.205326198 | Up |
| SPAG5 | -1.457841347 | 0.017093827 | 0.205326198 | Down |
| SLC25A14 | -1.005507523 | 0.017095092 | 0.205326198 | Down |
| BAG1 | -1.0138531 | 0.017103649 | 0.205326198 | Down |
| PMP22 | -1.029808297 | 0.017109266 | 0.205326198 | Down |
| XLOC_012524 | 1.223281848 | 0.017113478 | 0.205326198 | Up |
| XLOC_000280 | 1.93046535 | 0.017151149 | 0.205403399 | Up |
| KCNA6 | 1.362211092 | 0.017165339 | 0.205403399 | Up |
| RBM23 | -1.531858026 | 0.017165345 | 0.205403399 | Down |
| XLOC_004945 | 1.471800167 | 0.017165838 | 0.205403399 | Up |
| CNN3 | -1.108532671 | 0.017169679 | 0.205403399 | Down |
| XLOC_000008 | 1.335096587 | 0.017187523 | 0.205531757 | Up |
| SHISA7 | 1.435579049 | 0.017202965 | 0.205601204 | Up |
| XLOC_007101 | 1.399042664 | 0.017214788 | 0.205601204 | Up |
| HES1 | -1.323863265 | 0.017248744 | 0.205731713 | Down |
| DGKD | -1.149627549 | 0.017258267 | 0.205731713 | Down |
| XLOC_012630 | 1.589503482 | 0.01727784 | 0.205731713 | Up |
| MCAM | -1.337237657 | 0.017279046 | 0.205731713 | Down |
| ZNF395 | -1.046297555 | 0.017286849 | 0.205731713 | Down |
| XLOC_l2_000233 | 2.073746666 | 0.017309167 | 0.205878606 | Up |
| SNORD115-7 | 1.314724689 | 0.017352438 | 0.206013808 | Up |
| PNMAL2 | 1.591218604 | 0.017386029 | 0.206013808 | Up |
| BIRC8 | 1.000404495 | 0.017401072 | 0.206013808 | Up |
| OSCAR | 1.254679792 | 0.017424652 | 0.206013808 | Up |
| SLC6A8 | -1.514774752 | 0.017430608 | 0.206013808 | Down |
| RASL12 | -2.357564575 | 0.017450057 | 0.206013808 | Down |
| XLOC_001197 | 1.25682886 | 0.017456341 | 0.206013808 | Up |
| CD99P1 | -1.036883637 | 0.017473908 | 0.206013808 | Down |
| BTG3 | -1.014764912 | 0.017474226 | 0.206013808 | Down |
| RFXAP | -1.218936671 | 0.017480042 | 0.206013808 | Down |
| MYL6B | -1.00917834 | 0.017482043 | 0.206013808 | Down |
| DHTKD1 | -1.009224653 | 0.017497505 | 0.206013808 | Down |
| XLOC_013664 | 1.653396168 | 0.017499711 | 0.206013808 | Up |
| ECM1 | -1.40682938 | 0.017504849 | 0.206013808 | Down |
| XLOC_002909 | 1.077435305 | 0.01752187 | 0.206013808 | Up |
| ANKRD34A | 1.063606138 | 0.017529252 | 0.206013808 | Up |
| POM121L4P | 2.103709362 | 0.017555695 | 0.206013808 | Up |
| XLOC_010699 | 1.216667264 | 0.017569352 | 0.206013808 | Up |
| XLOC_013535 | 1.060060234 | 0.017586935 | 0.206013808 | Up |
| FER | -1.135120122 | 0.017591173 | 0.206013808 | Down |
| PEAK1 | -1.220503472 | 0.017625663 | 0.206013808 | Down |
| XLOC_003073 | 2.005279541 | 0.0176279 | 0.206013808 | Up |
| GUSB | -1.128633733 | 0.017640873 | 0.206013808 | Down |
| CCL27 | 1.418726848 | 0.017662669 | 0.206013808 | Up |
| MUC3 | 2.251426848 | 0.01766907 | 0.206013808 | Up |
| XLOC_l2_007595 | 1.150914915 | 0.017693718 | 0.206013808 | Up |
| HIST2H2AC | -1.039872722 | 0.01772929 | 0.206013808 | Down |
| XLOC_001242 | 1.249492286 | 0.017737096 | 0.206013808 | Up |
| ASPN | -1.456883636 | 0.017754519 | 0.206013808 | Down |
| ANG | -1.342439315 | 0.017759416 | 0.206013808 | Down |
| BCORL1 | -1.298980447 | 0.017762639 | 0.206013808 | Down |
| EVL | -1.121308047 | 0.017787814 | 0.206140283 | Down |
| XLOC_011951 | 1.626821883 | 0.017825145 | 0.206303761 | Up |
| ZSWIM2 | 1.157922032 | 0.017827543 | 0.206303761 | Up |
| XLOC_005111 | -1.236463087 | 0.017837596 | 0.206303761 | Down |
| RPL14 | -1.141602653 | 0.017863554 | 0.206303761 | Down |
| ANKRA2 | -1.127712856 | 0.017865301 | 0.206303761 | Down |
| PSMA8 | 1.063862709 | 0.017883824 | 0.206303761 | Up |
| C19orf52 | -1.174900327 | 0.017891425 | 0.206303761 | Down |
| TTTY5 | 1.597225713 | 0.017915155 | 0.206303761 | Up |
| GPR172B | 1.00585728 | 0.017929514 | 0.206303761 | Up |
| PJA1 | -1.033588285 | 0.017949554 | 0.206303761 | Down |
| FYN | -1.049654753 | 0.017979833 | 0.206303761 | Down |
| XLOC_003296 | 1.617517545 | 0.017996833 | 0.206303761 | Up |
| XLOC_012614 | 2.104628725 | 0.017998668 | 0.206303761 | Up |
| OLFML3 | -1.474369012 | 0.018005214 | 0.206303761 | Down |
| CD164L2 | 2.35080371 | 0.018030391 | 0.206303761 | Up |
| RAB6B | -1.146833669 | 0.018033438 | 0.206303761 | Down |
| SLC25A27 | -1.171808805 | 0.018080634 | 0.206303761 | Down |
| BBIP1 | -1.036780969 | 0.018081922 | 0.206303761 | Down |
| ZMYM2 | -1.146581207 | 0.018082351 | 0.206303761 | Down |
| CAMK2N2 | 1.056082666 | 0.018085875 | 0.206303761 | Up |
| XLOC_013552 | 2.396463729 | 0.018093128 | 0.206303761 | Up |
| XLOC_004965 | 1.05082407 | 0.018100542 | 0.206303761 | Up |
| XLOC_010241 | 1.264708667 | 0.018104468 | 0.206303761 | Up |
| XLOC_008337 | 2.188495217 | 0.018110884 | 0.206303761 | Up |
| AP3S2 | -1.025046751 | 0.018112894 | 0.206303761 | Down |
| XLOC_003865 | 1.050367712 | 0.018123945 | 0.206303761 | Up |
| ZNHIT6 | -1.455611185 | 0.018129563 | 0.206303761 | Down |
| FOXP3 | 1.507233188 | 0.018134315 | 0.206303761 | Up |
| CYBRD1 | -1.136088042 | 0.018140196 | 0.206303761 | Down |
| LARP6 | -1.267503513 | 0.018144823 | 0.206303761 | Down |
| XLOC_005917 | 1.589097588 | 0.018150281 | 0.206303761 | Up |
| LPAR1 | -1.340319527 | 0.018153393 | 0.206303761 | Down |
| KCNA10 | 1.228533798 | 0.018169695 | 0.206303761 | Up |
| SPARCL1 | -2.210241319 | 0.018192063 | 0.206303761 | Down |
| EIF3E | -1.026989932 | 0.018221727 | 0.206303761 | Down |
| CXCL12 | -2.027311973 | 0.018233965 | 0.206303761 | Down |
| ZZEF1 | -1.135445117 | 0.018235746 | 0.206303761 | Down |
| XLOC_l2_011118 | 1.587249365 | 0.018240049 | 0.206303761 | Up |
| ZFP90 | -1.221026074 | 0.018246124 | 0.206303761 | Down |
| NR1I2 | 1.291981391 | 0.018250432 | 0.206303761 | Up |
| HHIP | -1.853950108 | 0.018262356 | 0.206303761 | Down |
| KIR3DL1 | 1.191614927 | 0.018268656 | 0.206303761 | Up |
| XLOC_005550 | 1.033857601 | 0.018274578 | 0.206303761 | Up |
| XLOC_013274 | -1.12191711 | 0.018278905 | 0.206303761 | Down |
| XLOC_l2_012082 | 2.390823708 | 0.018280233 | 0.206303761 | Up |
| AKAP12 | -1.592612817 | 0.018286602 | 0.206303761 | Down |
| OTOA | 1.075460972 | 0.018291391 | 0.206303761 | Up |
| KLHL38 | 1.187566693 | 0.018293311 | 0.206303761 | Up |
| MUC16 | 1.174495923 | 0.018294025 | 0.206303761 | Up |
| LAMA2 | -1.103246363 | 0.018316278 | 0.206338705 | Down |
| XLOC_005803 | 1.026173449 | 0.018319157 | 0.206338705 | Up |
| PEX14 | -1.563059107 | 0.018334043 | 0.206425896 | Down |
| C21orf54 | 1.088278196 | 0.018361125 | 0.206526322 | Up |
| MGEA5 | -1.441832392 | 0.018416083 | 0.206624874 | Down |
| NDUFB10 | -1.036503791 | 0.018467084 | 0.206955981 | Down |
| EIF2B1 | -1.033530609 | 0.018517927 | 0.207043898 | Down |
| CDRT8 | 1.427756108 | 0.018558644 | 0.207248012 | Up |
| XLOC_014111 | -1.46255591 | 0.018577751 | 0.207248012 | Down |
| LYPLAL1 | -1.173088426 | 0.018583979 | 0.207248012 | Down |
| DPCR1 | 1.155677042 | 0.018592809 | 0.207248012 | Up |
| XLOC_007377 | 1.359422166 | 0.018612019 | 0.207248012 | Up |
| SALL2 | -1.351126098 | 0.018634759 | 0.207248012 | Down |
| TM4SF5 | 1.080293102 | 0.018665597 | 0.207248012 | Up |
| ESCO1 | -1.250209656 | 0.018667985 | 0.207248012 | Down |
| CPNE8 | -1.046205375 | 0.018705848 | 0.207248012 | Down |
| FTO | -1.003661252 | 0.018722621 | 0.207248012 | Down |
| KLHL9 | -1.204772214 | 0.01873496 | 0.207248012 | Down |
| XLOC_002303 | 1.377167395 | 0.018735376 | 0.207248012 | Up |
| WDFY3 | -1.141297844 | 0.018747678 | 0.207248012 | Down |
| AKAP10 | -1.064769821 | 0.018753009 | 0.207248012 | Down |
| RNF138P1 | -1.259369241 | 0.018803591 | 0.207248012 | Down |
| SYNPO2 | -1.577792729 | 0.018827455 | 0.207248012 | Down |
| ISLR | -1.448196331 | 0.018839106 | 0.207248012 | Down |
| XLOC_005899 | 1.126413438 | 0.018839203 | 0.207248012 | Up |
| SMPD3 | -1.301839639 | 0.018840176 | 0.207248012 | Down |
| PTPN4 | -1.177507601 | 0.01884139 | 0.207248012 | Down |
| XLOC_004199 | 1.397406828 | 0.018843114 | 0.207248012 | Up |
| CACNG2 | 1.823613908 | 0.018848428 | 0.207248012 | Up |
| XLOC_003463 | 1.124071634 | 0.018865687 | 0.207248012 | Up |
| TRAPPC2 | -1.075438387 | 0.018872085 | 0.207248012 | Down |
| HEG1 | -1.082956321 | 0.018877189 | 0.207248012 | Down |
| F7 | 1.251853915 | 0.018885614 | 0.207248012 | Up |
| XLOC_010464 | 1.174980717 | 0.018891843 | 0.207248012 | Up |
| ZNF615 | -1.126450626 | 0.018912417 | 0.207248012 | Down |
| C2orf27B | 1.166286503 | 0.018916376 | 0.207248012 | Up |
| PRKCG | 1.020882148 | 0.018939402 | 0.207421635 | Up |
| TRAPPC6B | -1.066995289 | 0.01896096 | 0.207579042 | Down |
| XLOC_003364 | 1.255235109 | 0.018987726 | 0.207585942 | Up |
| XLOC_004373 | 1.050330266 | 0.019001218 | 0.207585942 | Up |
| XLOC_l2_013467 | -1.224945582 | 0.019015683 | 0.207585942 | Down |
| MUC17 | 2.042551695 | 0.019018482 | 0.207585942 | Up |
| AK4 | -1.346909402 | 0.019020319 | 0.207585942 | Down |
| XLOC_l2_014197 | -1.163227626 | 0.019021578 | 0.207585942 | Down |
| XLOC_003603 | 1.679100483 | 0.019047659 | 0.207585942 | Up |
| XLOC_013299 | 1.039583253 | 0.019073894 | 0.207585942 | Up |
| REG1B | 1.079338537 | 0.019088284 | 0.207585942 | Up |
| FAM75A3 | 1.923527184 | 0.0190886 | 0.207585942 | Up |
| SCRT2 | 1.016695625 | 0.019109801 | 0.207585942 | Up |
| CENPC1 | -1.046214564 | 0.01911757 | 0.207585942 | Down |
| PDLIM7 | -1.437655801 | 0.019131621 | 0.207585942 | Down |
| FOXA2 | 1.873636103 | 0.019133716 | 0.207585942 | Up |
| PITPNC1 | -1.166258236 | 0.019152708 | 0.207585942 | Down |
| C20orf123 | 1.497799744 | 0.019166542 | 0.207585942 | Up |
| UQCR11 | -1.070355037 | 0.019173486 | 0.207585942 | Down |
| ZNF22 | -1.221144885 | 0.019181478 | 0.207585942 | Down |
| XLOC_001513 | 1.04949717 | 0.019187341 | 0.207585942 | Up |
| XLOC_000208 | 1.048224467 | 0.019205002 | 0.207585942 | Up |
| DEC1 | 1.272941515 | 0.019206452 | 0.207585942 | Up |
| XLOC_006793 | 1.07040598 | 0.01922233 | 0.207585942 | Up |
| EXOSC8 | -1.061754021 | 0.019273755 | 0.207585942 | Down |
| CDC25A | 1.272325071 | 0.01930764 | 0.207585942 | Up |
| XLOC_013759 | 1.528173648 | 0.019311985 | 0.207585942 | Up |
| PATE1 | 1.322816796 | 0.019315247 | 0.207585942 | Up |
| XLOC_011165 | 1.924782585 | 0.01933561 | 0.207585942 | Up |
| OR5R1 | 1.149265566 | 0.01936108 | 0.207585942 | Up |
| CHST3 | -1.071388738 | 0.019366883 | 0.207585942 | Down |
| VKORC1 | 2.060072063 | 0.019370354 | 0.207585942 | Up |
| XLOC_002852 | 1.369823615 | 0.019386727 | 0.207585942 | Up |
| MYH10 | -1.11662767 | 0.019408424 | 0.207585942 | Down |
| FLJ40039 | 1.230722093 | 0.01941529 | 0.207585942 | Up |
| NCR2 | 1.020772734 | 0.019417551 | 0.207585942 | Up |
| XLOC_l2_008550 | 1.938654454 | 0.019418945 | 0.207585942 | Up |
| GPR123 | 1.01690225 | 0.019439506 | 0.207585942 | Up |
| SVIL | -1.068725885 | 0.019476645 | 0.207585942 | Down |
| XLOC_l2_002067 | 1.316144306 | 0.0194801 | 0.207585942 | Up |
| NUDT17 | -1.089243428 | 0.019513261 | 0.207585942 | Down |
| DZANK1-AS1 | 1.084602258 | 0.019521668 | 0.207585942 | Up |
| SPTA1 | 1.2509324 | 0.019534703 | 0.207585942 | Up |
| KLF11 | -1.122299125 | 0.019542761 | 0.207585942 | Down |
| PTGER3 | -1.323969598 | 0.019543048 | 0.207585942 | Down |
| XLOC_011216 | 1.272960851 | 0.019556358 | 0.207585942 | Up |
| FLJ41130 | 2.082857125 | 0.019566964 | 0.207585942 | Up |
| C1orf182 | 1.406448305 | 0.019580855 | 0.207585942 | Up |
| CCKBR | 1.044650523 | 0.019587759 | 0.207585942 | Up |
| CPZ | -1.862428996 | 0.019598208 | 0.207585942 | Down |
| CLSTN1 | -1.305289291 | 0.019611154 | 0.207585942 | Down |
| VIM | -1.174327442 | 0.019617185 | 0.207585942 | Down |
| OTX2OS1 | 1.31763417 | 0.019635393 | 0.207585942 | Up |
| OR2AG2 | 1.05932925 | 0.019646747 | 0.207585942 | Up |
| PRSS23 | -1.808193482 | 0.019653069 | 0.207585942 | Down |
| MRPL55 | -1.135547647 | 0.019676821 | 0.207585942 | Down |
| RBM20 | 1.374033719 | 0.019682454 | 0.207585942 | Up |
| SLC16A2 | -1.26296801 | 0.019702653 | 0.207585942 | Down |
| STXBP4 | -1.001644687 | 0.019708005 | 0.207585942 | Down |
| XLOC_006310 | 1.118797746 | 0.019750234 | 0.207585942 | Up |
| VSX2 | 1.439041015 | 0.019773035 | 0.207585942 | Up |
| LAMC1 | -1.150301774 | 0.019794458 | 0.207585942 | Down |
| TRIM23 | -1.180293024 | 0.019795067 | 0.207585942 | Down |
| XLOC_005455 | 1.386599472 | 0.019844144 | 0.207673494 | Up |
| KRTAP9-7 | 1.090395683 | 0.01986213 | 0.207673494 | Up |
| OR51G1 | 1.263422895 | 0.019868376 | 0.207673494 | Up |
| DEFB107A | 1.358681179 | 0.019871302 | 0.207673494 | Up |
| SRRM3 | 1.347711979 | 0.019878437 | 0.207673494 | Up |
| XLOC_l2_012621 | -1.014633414 | 0.019900533 | 0.207673494 | Down |
| INTS4L1 | 1.091420924 | 0.019904936 | 0.207673494 | Up |
| XLOC_l2_015336 | 1.125196662 | 0.019906717 | 0.207673494 | Up |
| XLOC_012395 | 1.111232341 | 0.019918699 | 0.207673494 | Up |
| MGC24103 | -1.055542695 | 0.019928532 | 0.207673494 | Down |
| XLOC_000885 | 1.31045753 | 0.019929618 | 0.207673494 | Up |
| XLOC_008567 | 1.499937698 | 0.019936121 | 0.207673494 | Up |
| GIMAP5 | -1.223296377 | 0.019941412 | 0.207673494 | Down |
| GPX7 | -1.278506142 | 0.019966539 | 0.207793801 | Down |
| GIMAP1 | -1.154540988 | 0.019996783 | 0.207793801 | Down |
| ORC1 | 1.045000967 | 0.020005028 | 0.207793801 | Up |
| XIST | -3.442595173 | 0.020010981 | 0.207793801 | Down |
| FLJ13197 | -1.082633312 | 0.020031305 | 0.207793801 | Down |
| INTS3 | -1.036758535 | 0.020041256 | 0.207793801 | Down |
| LEPREL4 | -1.06007504 | 0.020059433 | 0.207793801 | Down |
| NUFIP2 | -1.16923453 | 0.020075308 | 0.207793801 | Down |
| ZNF484 | -1.052811302 | 0.020076257 | 0.207793801 | Down |
| PDILT | 1.92681658 | 0.020092883 | 0.207793801 | Up |
| TSIX | -4.600696795 | 0.020095813 | 0.207793801 | Down |
| EPM2AIP1 | -1.281278223 | 0.020102125 | 0.207793801 | Down |
| XLOC_011590 | 1.149100806 | 0.020126702 | 0.207793801 | Up |
| CYP2C9 | 1.33797536 | 0.020127076 | 0.207793801 | Up |
| XLOC_004353 | 1.084678709 | 0.02016645 | 0.207793801 | Up |
| XLOC_013037 | 1.114042177 | 0.02018313 | 0.207793801 | Up |
| C2orf67 | -1.036692959 | 0.020214206 | 0.207793801 | Down |
| MRPL48 | -1.183000146 | 0.020237411 | 0.207793801 | Down |
| SNHG13 | -1.257624623 | 0.020271954 | 0.207793801 | Down |
| FTL | -1.054561024 | 0.020275045 | 0.207793801 | Down |
| ATP5G2 | -1.183210251 | 0.020283516 | 0.207793801 | Down |
| C15orf61 | -1.231085666 | 0.020296251 | 0.207793801 | Down |
| SNORD76 | -1.149637993 | 0.020309 | 0.207793801 | Down |
| LSAMP | -1.198997559 | 0.020327817 | 0.207793801 | Down |
| XLOC_005223 | 1.667612186 | 0.020340633 | 0.207793801 | Up |
| PSORS1C2 | 1.64519265 | 0.020383117 | 0.207793801 | Up |
| UQCRQ | -1.032020514 | 0.020389057 | 0.207793801 | Down |
| CLRN1-AS1 | 1.140613446 | 0.020400671 | 0.207793801 | Up |
| PCDHA5 | 1.648665629 | 0.020415269 | 0.207793801 | Up |
| XLOC_004263 | 1.752870528 | 0.02041608 | 0.207793801 | Up |
| XLOC_005119 | 1.112647079 | 0.020416909 | 0.207793801 | Up |
| CETN2 | -1.026971199 | 0.020424983 | 0.207793801 | Down |
| CEACAM16 | -1.194922012 | 0.020460465 | 0.207793801 | Down |
| XLOC_003994 | 1.323591229 | 0.020462947 | 0.207793801 | Up |
| NARF | -1.192535587 | 0.020466624 | 0.207793801 | Down |
| MSH6 | -1.034272418 | 0.020480564 | 0.207793801 | Down |
| OR1C1 | 1.200295602 | 0.020482332 | 0.207793801 | Up |
| SPATA22 | 1.045194413 | 0.020487713 | 0.207793801 | Up |
| VPS8 | -1.050314009 | 0.020487856 | 0.207793801 | Down |
| ZNF449 | -1.033483874 | 0.020515998 | 0.207817118 | Down |
| XLOC_004725 | 1.582398896 | 0.020560375 | 0.208091804 | Up |
| STK17B | -1.53382131 | 0.020575455 | 0.208091804 | Down |
| ZNF506 | -1.41598011 | 0.020579093 | 0.208091804 | Down |
| GUCY2E | 1.66360245 | 0.02058483 | 0.208091804 | Up |
| COMMD5 | -1.00517726 | 0.020592381 | 0.208091804 | Down |
| PRAMEF11 | 1.027882939 | 0.020617701 | 0.20817899 | Up |
| XLOC_003613 | 1.184934015 | 0.020643309 | 0.20817899 | Up |
| HSPA13 | -1.043957944 | 0.020671029 | 0.20817899 | Down |
| CTGF | -1.545480424 | 0.02068968 | 0.20817899 | Down |
| PKNOX1 | -1.064186641 | 0.020706471 | 0.20817899 | Down |
| GFRA1 | -1.029128125 | 0.020720122 | 0.20817899 | Down |
| RPL13A | -1.07722621 | 0.020723796 | 0.20817899 | Down |
| OR8U1 | 1.210895637 | 0.020741861 | 0.20817899 | Up |
| RNF34 | -1.261222065 | 0.020750935 | 0.20817899 | Down |
| PAX3 | 1.062100427 | 0.02076038 | 0.20817899 | Up |
| XLOC_006044 | 1.470072064 | 0.020766739 | 0.20817899 | Up |
| KIR2DL4 | 1.752532842 | 0.020796292 | 0.208340574 | Up |
| XLOC_002318 | 1.072958025 | 0.020810503 | 0.208340574 | Up |
| XLOC_l2_014876 | 1.257350082 | 0.020814698 | 0.208340574 | Up |
| ANTXR1 | -1.329781225 | 0.020818962 | 0.208340574 | Down |
| MRPL14 | -1.135556507 | 0.020846774 | 0.208415381 | Down |
| ZNF362 | -1.01773264 | 0.02085652 | 0.208415381 | Down |
| KRT72 | 1.27362546 | 0.020873355 | 0.208415381 | Up |
| XLOC_004428 | 1.745225232 | 0.020873682 | 0.208415381 | Up |
| XLOC_012607 | 1.133265553 | 0.020876886 | 0.208415381 | Up |
| ULK1 | -1.00537766 | 0.020911446 | 0.208688284 | Down |
| XLOC_003464 | 1.157373362 | 0.020940275 | 0.208853783 | Up |
| KIAA0947 | -1.40473591 | 0.020942488 | 0.208853783 | Down |
| XLOC_002959 | 1.324958252 | 0.020964731 | 0.208857506 | Up |
| TACC2 | -1.060895943 | 0.020975188 | 0.208857506 | Down |
| RGS2 | -1.333929357 | 0.020980908 | 0.208857506 | Down |
| KRBOX1-AS1 | 1.167808354 | 0.020985202 | 0.208857506 | Up |
| PDE8A | -1.181247009 | 0.020993465 | 0.208857506 | Down |
| PROS1 | -1.277458441 | 0.02102844 | 0.20895317 | Down |
| ERVH-3 | 1.189586824 | 0.02103196 | 0.20895317 | Up |
| STARD3NL | -1.084051604 | 0.021044349 | 0.20895317 | Down |
| XLOC_001618 | 1.058734804 | 0.021046016 | 0.20895317 | Up |
| XLOC_008815 | 1.160131431 | 0.02106094 | 0.20895317 | Up |
| FLJ46120 | 1.067510617 | 0.02107099 | 0.208981107 | Up |
| OR2B6 | 1.041155605 | 0.021118533 | 0.209041049 | Up |
| DSCR8 | 1.005451906 | 0.021125451 | 0.209041049 | Up |
| CLN5 | -1.434299813 | 0.021134409 | 0.209041049 | Down |
| C17orf58 | -1.139966997 | 0.021134541 | 0.209041049 | Down |
| XLOC_005898 | 1.174830907 | 0.021154516 | 0.209041049 | Up |
| XLOC_l2_016079 | 2.223109911 | 0.021159559 | 0.209041049 | Up |
| C7orf69 | 1.248133753 | 0.02115974 | 0.209041049 | Up |
| MAGEA12 | 1.259532473 | 0.021160878 | 0.209041049 | Up |
| XLOC_014022 | 1.0783703 | 0.021162019 | 0.209041049 | Up |
| XLOC_l2_010933 | 1.218060936 | 0.021165707 | 0.209041049 | Up |
| EIF4B | -1.132424955 | 0.021197039 | 0.209041049 | Down |
| PRKCE | -1.023889138 | 0.021206635 | 0.209041049 | Down |
| SLC25A38 | -1.093040394 | 0.021218305 | 0.209041049 | Down |
| OMP | -1.054724067 | 0.021218502 | 0.209041049 | Down |
| XLOC_007567 | 1.245460583 | 0.021224349 | 0.209041049 | Up |
| TUBB4A | 2.095665842 | 0.021257054 | 0.209041049 | Up |
| XLOC_006753 | -1.039240564 | 0.021305722 | 0.209049655 | Down |
| RPS9 | -1.187312365 | 0.021310991 | 0.209049655 | Down |
| LGALS1 | -1.420677155 | 0.021321183 | 0.209049655 | Down |
| ITIH6 | 2.301994005 | 0.021331768 | 0.209049655 | Up |
| CHMP1B | -1.206214662 | 0.021351899 | 0.209049655 | Down |
| TNFSF4 | -1.068832014 | 0.02139405 | 0.209049655 | Down |
| EXTL2 | -1.49883943 | 0.021397379 | 0.209049655 | Down |
| C1QTNF3 | -1.370166619 | 0.021414821 | 0.209049655 | Down |
| XLOC_014175 | 1.475250719 | 0.021425471 | 0.209049655 | Up |
| MUC6 | 1.572757634 | 0.021450742 | 0.209049655 | Up |
| MUS81 | -1.136886591 | 0.021478301 | 0.209049655 | Down |
| SRSF3 | -1.01771976 | 0.021488874 | 0.209049655 | Down |
| PILRB | -1.150648258 | 0.021501518 | 0.209049655 | Down |
| XLOC_012173 | 1.75987711 | 0.021508378 | 0.209049655 | Up |
| CATSPERB | 1.009065299 | 0.02151689 | 0.209049655 | Up |
| PHKA2 | -1.038436095 | 0.021522418 | 0.209049655 | Down |
| TRAM2 | -1.609206911 | 0.021547146 | 0.209049655 | Down |
| DYNC1H1 | -1.117080195 | 0.021583628 | 0.209049655 | Down |
| XLOC_012506 | 2.927761763 | 0.021622649 | 0.209049655 | Up |
| XLOC_009524 | 1.11064905 | 0.021637376 | 0.209049655 | Up |
| MACF1 | -1.110524605 | 0.021642758 | 0.209049655 | Down |
| CLEC5A | 1.235671444 | 0.021679723 | 0.209049655 | Up |
| ERC1 | -1.130186429 | 0.021680839 | 0.209049655 | Down |
| C12orf45 | -1.067520989 | 0.021738655 | 0.209049655 | Down |
| GUK1 | -1.116507144 | 0.021775062 | 0.209049655 | Down |
| BTF3 | -1.035467439 | 0.021776952 | 0.209049655 | Down |
| XLOC_010908 | 1.455910608 | 0.021778073 | 0.209049655 | Up |
| XLOC_005359 | 1.630933423 | 0.021780637 | 0.209049655 | Up |
| XLOC_004377 | 1.140250215 | 0.021790136 | 0.209049655 | Up |
| XLOC_009913 | 1.261970789 | 0.021835038 | 0.20923253 | Up |
| TEX28 | 1.02077812 | 0.021844048 | 0.209249466 | Up |
| AZIN1 | -1.008340529 | 0.021893348 | 0.209389648 | Down |
| FGF14 | -1.214076246 | 0.021902186 | 0.209389648 | Down |
| GOLGA6L5 | 1.514920965 | 0.02192391 | 0.209389648 | Up |
| XLOC_008944 | 1.362437349 | 0.021932037 | 0.209398044 | Up |
| XLOC_012605 | 1.636716386 | 0.021956598 | 0.209526746 | Up |
| XLOC_003521 | 1.315941711 | 0.021969359 | 0.209526746 | Up |
| RPLP2 | -1.009695906 | 0.022014443 | 0.209526746 | Down |
| IL28B | 1.634883964 | 0.022038441 | 0.209526746 | Up |
| XLOC_006972 | 1.411883861 | 0.022066553 | 0.209526746 | Up |
| TMC2 | 1.781873362 | 0.022070059 | 0.209526746 | Up |
| PARM1 | -1.5994138 | 0.022076059 | 0.209526746 | Down |
| MRVI1 | -1.519687316 | 0.022118309 | 0.209629079 | Down |
| ZBTB25 | -1.055956141 | 0.02213168 | 0.209629079 | Down |
| CD81 | 1.274126057 | 0.022189404 | 0.209629079 | Up |
| DDX11L9 | 1.525004615 | 0.022210168 | 0.209629079 | Up |
| SLC22A13 | 1.585239081 | 0.022219355 | 0.209629079 | Up |
| XLOC_000243 | 1.563745116 | 0.022243636 | 0.209629079 | Up |
| XAGE1A | 1.474488086 | 0.02224457 | 0.209629079 | Up |
| TRPC1 | -1.293392629 | 0.022249617 | 0.209629079 | Down |
| C1orf105 | 1.004519264 | 0.022256127 | 0.209629079 | Up |
| EMILIN2 | -1.039983449 | 0.022269021 | 0.209629079 | Down |
| PAPD4 | -1.290209164 | 0.022272504 | 0.209629079 | Down |
| RFX7 | -1.013159747 | 0.02227331 | 0.209629079 | Down |
| XLOC_008558 | 1.108652214 | 0.022291649 | 0.209629079 | Up |
| RASGRP2 | 1.025842991 | 0.022297261 | 0.209629079 | Up |
| XLOC_005788 | 1.148969458 | 0.022345776 | 0.209687201 | Up |
| CRY2 | -1.044131953 | 0.022360645 | 0.209687201 | Down |
| CDC16 | -1.174482876 | 0.022361707 | 0.209687201 | Down |
| C9orf173 | 1.161094496 | 0.022365843 | 0.209687201 | Up |
| XLOC_006356 | 1.523116351 | 0.022381341 | 0.209737045 | Up |
| PDCD1 | 1.007193736 | 0.022398992 | 0.209822741 | Up |
| KGFLP1 | -1.088745936 | 0.022413702 | 0.209822741 | Down |
| HSPA4L | -1.15898144 | 0.022426798 | 0.209822741 | Down |
| CHCHD3 | -1.203234326 | 0.022476276 | 0.210081554 | Down |
| TTTY20 | 1.450018497 | 0.022515696 | 0.210118962 | Up |
| Q5C5N0 | 1.122494276 | 0.022553056 | 0.210119423 | Up |
| KLF12 | -1.062697711 | 0.022589634 | 0.210130776 | Down |
| LONP2 | -1.063819723 | 0.022608788 | 0.210130776 | Down |
| RPL30 | -1.207141751 | 0.022619502 | 0.210130776 | Down |
| IL33 | -1.368756855 | 0.02263944 | 0.210135076 | Down |
| DCTD | -1.229562946 | 0.02265081 | 0.210135076 | Down |
| XLOC_l2_005814 | 1.437553535 | 0.022673856 | 0.210135076 | Up |
| PPARGC1A | -1.305399343 | 0.022687287 | 0.210135076 | Down |
| FAM104A | -1.060772805 | 0.022688636 | 0.210135076 | Down |
| C11orf54 | -1.211207111 | 0.022700203 | 0.210135076 | Down |
| XLOC_002577 | -1.235445205 | 0.022746775 | 0.210210517 | Down |
| MOXD1 | -2.364441648 | 0.022749236 | 0.210210517 | Down |
| SF3B14 | -1.194880566 | 0.022778903 | 0.210210517 | Down |
| NLRP8 | 1.192720276 | 0.022787205 | 0.210210517 | Up |
| PLD6 | -1.203934207 | 0.022790561 | 0.210210517 | Down |
| UNC45B | -1.01699635 | 0.02282965 | 0.210214166 | Down |
| C1orf50 | -1.027658944 | 0.022866482 | 0.210214166 | Down |
| DACT1 | -1.650890122 | 0.022868822 | 0.210214166 | Down |
| MRPL9 | -1.028557827 | 0.022876294 | 0.210215969 | Down |
| PYROXD1 | -1.004217754 | 0.022903665 | 0.210323902 | Down |
| PTDSS2 | -1.071417541 | 0.022921954 | 0.210323902 | Down |
| PHIP | -1.013263263 | 0.022955113 | 0.210404877 | Down |
| PP12613 | 1.316832051 | 0.022989313 | 0.210542736 | Up |
| NDUFA11 | -1.086455142 | 0.023012423 | 0.210542736 | Down |
| XLOC_004600 | 1.336837573 | 0.023016672 | 0.210542736 | Up |
| SELENBP1 | -1.170349838 | 0.023018089 | 0.210542736 | Down |
| LHX8 | -1.072074878 | 0.023078868 | 0.210542736 | Down |
| NIPA1 | -1.04572866 | 0.023116295 | 0.210542736 | Down |
| XLOC_008604 | 1.000208222 | 0.023136015 | 0.210542736 | Up |
| C7orf49 | -1.034853942 | 0.023136667 | 0.210542736 | Down |
| NCKAP1L | 1.413434897 | 0.023140013 | 0.210542736 | Up |
| IMMP2L | -1.246705411 | 0.02316169 | 0.210542736 | Down |
| GIF | 1.186120677 | 0.023174602 | 0.210542736 | Up |
| ZNF638-IT1 | -1.043076585 | 0.0231871 | 0.210542736 | Down |
| XLOC_013100 | 1.041605305 | 0.023198123 | 0.210542736 | Up |
| XLOC_007057 | 1.066669895 | 0.02320009 | 0.210542736 | Up |
| RPL26 | -1.06663826 | 0.023212865 | 0.210542736 | Down |
| ADAM19 | -1.005413611 | 0.023246974 | 0.210542736 | Down |
| XLOC_001792 | 1.061344361 | 0.023247394 | 0.210542736 | Up |
| TCEAL5 | -1.074353439 | 0.023280776 | 0.210542736 | Down |
| LTBP1 | -1.34168049 | 0.023291974 | 0.210542736 | Down |
| KIAA0240 | -1.051895793 | 0.023335289 | 0.210542736 | Down |
| XLOC_l2_012835 | 2.070050106 | 0.023359854 | 0.210542736 | Up |
| EEF1G | -1.073969307 | 0.02336771 | 0.210542736 | Down |
| UNK | -1.121134852 | 0.023384246 | 0.210542736 | Down |
| C1orf111 | 1.081611302 | 0.023393464 | 0.210542736 | Up |
| XLOC_009475 | 1.291567246 | 0.023420018 | 0.210542736 | Up |
| TM7SF4 | 1.30443763 | 0.023431242 | 0.210542736 | Up |
| MMP3 | 1.633356825 | 0.023434748 | 0.210542736 | Up |
| ADCY7 | -1.155794075 | 0.023442297 | 0.210542736 | Down |
| C1orf189 | 1.97674038 | 0.023461265 | 0.210542736 | Up |
| ROCK2 | -1.237880676 | 0.023464635 | 0.210542736 | Down |
| CREB3L3 | 1.250364705 | 0.023493407 | 0.210542736 | Up |
| RPS15 | -1.18680173 | 0.023505651 | 0.210542736 | Down |
| EGF | 1.062542515 | 0.023511332 | 0.210542736 | Up |
| NUCKS1 | -1.053210177 | 0.023522954 | 0.210542736 | Down |
| OR5F1 | 1.734823915 | 0.02353986 | 0.210542736 | Up |
| XLOC_002882 | 1.227942047 | 0.02354579 | 0.210542736 | Up |
| XLOC_008300 | 1.633946418 | 0.023560016 | 0.210542736 | Up |
| RSRC2 | -1.005722077 | 0.023565635 | 0.210542736 | Down |
| XLOC_012249 | 1.950136867 | 0.023582524 | 0.210542736 | Up |
| XLOC_010685 | 1.105606296 | 0.023596428 | 0.210542736 | Up |
| XLOC_002383 | 1.171584247 | 0.023599067 | 0.210542736 | Up |
| XLOC_l2_013858 | 1.001265266 | 0.023604978 | 0.210542736 | Up |
| XLOC_014356 | 1.968343078 | 0.023614868 | 0.210542736 | Up |
| EXOSC10 | -1.061469378 | 0.023634762 | 0.210542736 | Down |
| DSE | -1.212157765 | 0.023649118 | 0.210542736 | Down |
| XYLT1 | -1.130544916 | 0.023653493 | 0.210542736 | Down |
| XLOC_l2_012162 | 1.489227453 | 0.023670029 | 0.210542736 | Up |
| XLOC_001650 | 1.04456965 | 0.023690908 | 0.210542736 | Up |
| FAM48A | -1.174734717 | 0.023705347 | 0.210542736 | Down |
| XLOC_012421 | 1.563428146 | 0.023713832 | 0.210542736 | Up |
| RPL18A | -1.165372358 | 0.023730079 | 0.210542736 | Down |
| TMEM175 | -1.155407579 | 0.023737034 | 0.210542736 | Down |
| FLJ44674 | 1.560524893 | 0.023739466 | 0.210542736 | Up |
| INHBC | 1.762886005 | 0.023739519 | 0.210542736 | Up |
| UBE2V1 | -1.031997139 | 0.023751682 | 0.210542736 | Down |
| TMEM196 | -1.060515815 | 0.023759605 | 0.210542736 | Down |
| ROBO4 | -1.036835484 | 0.023765262 | 0.210542736 | Down |
| SLC40A1 | -1.054493343 | 0.023813077 | 0.210542736 | Down |
| XLOC_002198 | 1.315432957 | 0.023826199 | 0.210542736 | Up |
| XLOC_004315 | 1.041476168 | 0.023828862 | 0.210542736 | Up |
| XLOC_011469 | 1.246836771 | 0.023836264 | 0.210542736 | Up |
| XLOC_011615 | 1.225518843 | 0.023894772 | 0.210727672 | Up |
| SNORD115-23 | 1.1985347 | 0.023908024 | 0.210728862 | Up |
| XLOC_009305 | 1.23953515 | 0.023910154 | 0.210728862 | Up |
| XLOC_006617 | 1.178370017 | 0.023917237 | 0.210728862 | Up |
| CALCOCO2 | -1.114160628 | 0.023944495 | 0.210844381 | Down |
| MSN | -1.103872692 | 0.023971619 | 0.210954626 | Down |
| FAM27L | 1.0278503 | 0.023994968 | 0.210967325 | Up |
| XLOC_003160 | 1.460472168 | 0.024016909 | 0.211095986 | Up |
| XLOC_l2_015661 | 2.778833548 | 0.024042234 | 0.211171111 | Up |
| XLOC_013108 | 1.134940428 | 0.024055848 | 0.211171111 | Up |
| C9orf21 | -1.532485137 | 0.02408669 | 0.211259434 | Down |
| MUC12 | 2.607149711 | 0.024101739 | 0.211263152 | Up |
| SLC39A13 | -1.030478162 | 0.024127098 | 0.211302617 | Down |
| XLOC_003738 | 1.261959517 | 0.024130743 | 0.211302617 | Up |
| ID4 | -1.372511758 | 0.024148353 | 0.211302617 | Down |
| C14orf133 | -1.00995714 | 0.024296219 | 0.212048417 | Down |
| FAM135B | 1.138169468 | 0.02431947 | 0.212048417 | Up |
| XLOC_007197 | 1.134672484 | 0.024347832 | 0.212133059 | Up |
| HOXD11 | -1.046112571 | 0.024379472 | 0.212161112 | Down |
| XLOC_004115 | 1.13969015 | 0.024421516 | 0.212161112 | Up |
| NEAT1 | -1.122848836 | 0.024428347 | 0.212161112 | Down |
| SRSF4 | -1.034398808 | 0.024434661 | 0.212161112 | Down |
| XLOC_003147 | 1.149536566 | 0.024445439 | 0.212161112 | Up |
| SRSF7 | -1.49964956 | 0.024452024 | 0.212161112 | Down |
| GPR137C | 1.041478582 | 0.024461745 | 0.212161112 | Up |
| C15orf29 | -1.036646689 | 0.024469826 | 0.212161112 | Down |
| XLOC_001263 | 1.406641851 | 0.024482968 | 0.212161112 | Up |
| ZNF580 | -1.119584259 | 0.024515922 | 0.212230497 | Down |
| XLOC_013469 | 1.079831269 | 0.024568342 | 0.212230497 | Up |
| HIST1H2AM | -1.015798937 | 0.024590578 | 0.212230497 | Down |
| FAM19A5 | -1.182677724 | 0.024631222 | 0.212230497 | Down |
| MYOM3 | 1.160783317 | 0.024644987 | 0.212230497 | Up |
| XLOC_006560 | 1.022260893 | 0.024657863 | 0.212230497 | Up |
| NR2E3 | 1.302389771 | 0.024669635 | 0.212248491 | Up |
| PBX2 | -1.054287199 | 0.024701313 | 0.212373668 | Down |
| XLOC_006637 | 1.140409063 | 0.024714076 | 0.212373668 | Up |
| RUNX1T1 | -1.703681673 | 0.024739045 | 0.212373668 | Down |
| XLOC_013051 | 1.063696904 | 0.024751425 | 0.212382956 | Up |
| XLOC_l2_011954 | 1.221885056 | 0.024765827 | 0.212443444 | Up |
| XLOC_008669 | 1.394801146 | 0.024835025 | 0.212577346 | Up |
| ITGA1 | -1.186980548 | 0.024851539 | 0.212610549 | Down |
| ZNF791 | -1.169117844 | 0.024886818 | 0.212725971 | Down |
| DKFZp434J0226 | 1.030558346 | 0.024894356 | 0.212725971 | Up |
| OR1L4 | 1.003602494 | 0.024918341 | 0.212725971 | Up |
| XLOC_001392 | 1.061595135 | 0.024943682 | 0.212725971 | Up |
| ZNF286B | -1.092303121 | 0.024960855 | 0.212725971 | Down |
| C5orf25 | -1.02087355 | 0.024973611 | 0.212725971 | Down |
| SRL | -1.156308648 | 0.024979389 | 0.212725971 | Down |
| OR8D1 | 1.599440659 | 0.024982662 | 0.212725971 | Up |
| EIF3I | -1.0707181 | 0.025010318 | 0.212725971 | Down |
| PET117 | -1.020852649 | 0.025014823 | 0.212725971 | Down |
| XLOC_005730 | 1.283490227 | 0.025035579 | 0.212725971 | Up |
| NCRNA00249 | 1.371313809 | 0.025036339 | 0.212725971 | Up |
| XLOC_007116 | 1.126994322 | 0.025044479 | 0.212725971 | Up |
| ZBTB41 | -1.29361783 | 0.025113023 | 0.21277022 | Down |
| DZIP1L | -1.21411479 | 0.025120599 | 0.21277022 | Down |
| XLOC_003405 | 1.882618244 | 0.025131022 | 0.212796119 | Up |
| XLOC_l2_006704 | 1.945357856 | 0.025150948 | 0.212902443 | Up |
| LANCL1 | -1.441988544 | 0.025246631 | 0.213274972 | Down |
| XLOC_007323 | 2.19239084 | 0.025266886 | 0.213306839 | Up |
| SERTAD2 | -1.272026978 | 0.025289672 | 0.213306839 | Down |
| LGALS17A | 1.313711225 | 0.025327766 | 0.213306839 | Up |
| XLOC_002695 | 1.53439974 | 0.025442518 | 0.213306839 | Up |
| RNASE4 | -1.345845823 | 0.025449192 | 0.213306839 | Down |
| RASIP1 | -1.19616665 | 0.025456686 | 0.213306839 | Down |
| GPD1L | -1.040018488 | 0.025469636 | 0.213306839 | Down |
| XLOC_009728 | 1.224388343 | 0.02547473 | 0.213306839 | Up |
| PCDHB4 | -1.321802026 | 0.025506915 | 0.213306839 | Down |
| XLOC_011918 | 1.938451213 | 0.025516 | 0.213306839 | Up |
| RABEP1 | -1.007542021 | 0.025529558 | 0.213306839 | Down |
| CELF3 | 1.072740415 | 0.025552965 | 0.213306839 | Up |
| PRSS1 | 1.371676127 | 0.025564461 | 0.213306839 | Up |
| ATMIN | 1.518824429 | 0.025570413 | 0.213306839 | Up |
| KLK15 | 1.261126994 | 0.025572048 | 0.213306839 | Up |
| TRIM31 | 1.422796501 | 0.025600996 | 0.213306839 | Up |
| XLOC_011107 | 1.242368891 | 0.025607117 | 0.213306839 | Up |
| AMOTL2 | -1.192109906 | 0.025624591 | 0.213306839 | Down |
| SST | 1.159106365 | 0.02562838 | 0.213306839 | Up |
| RCSD1 | -1.144820295 | 0.025647381 | 0.213306839 | Down |
| TDRG1 | 1.041908004 | 0.025649676 | 0.213306839 | Up |
| XLOC_000977 | 1.239533507 | 0.025658004 | 0.213306839 | Up |
| C10orf88 | -1.036994082 | 0.025685116 | 0.213306839 | Down |
| CDSN | 1.730680196 | 0.025690628 | 0.213306839 | Up |
| XLOC_l2_008599 | 1.443114351 | 0.025692583 | 0.213306839 | Up |
| SPATS2 | -1.060445646 | 0.025735408 | 0.213306839 | Down |
| TLX3 | -1.124898942 | 0.025760117 | 0.213306839 | Down |
| EIF3L | -1.180918875 | 0.025782991 | 0.213306839 | Down |
| C4orf40 | 1.378105521 | 0.025836259 | 0.213306839 | Up |
| PLEKHA4 | -1.472151484 | 0.025839122 | 0.213306839 | Down |
| GOLGA6L7P | 1.138408645 | 0.025852557 | 0.213306839 | Up |
| FANCM | -1.067542119 | 0.025853173 | 0.213306839 | Down |
| XLOC_l2_010687 | 1.064654273 | 0.025868986 | 0.213306839 | Up |
| PPAPDC1A | -1.153116686 | 0.025875798 | 0.213306839 | Down |
| NKTR | -1.248688294 | 0.025897965 | 0.213306839 | Down |
| TMEM30B | -1.045038822 | 0.025905055 | 0.213306839 | Down |
| HNF1A | 1.368372666 | 0.025929653 | 0.213306839 | Up |
| XLOC_009516 | 1.189451552 | 0.025992282 | 0.213502919 | Up |
| GIMAP7 | -1.241104261 | 0.025994969 | 0.213502919 | Down |
| LY6K | -1.685646882 | 0.02600537 | 0.213502919 | Down |
| CREBZF | 1.087255378 | 0.026011193 | 0.213502919 | Up |
| XLOC_l2_011399 | 1.646367045 | 0.026014306 | 0.213502919 | Up |
| XLOC_000681 | 1.22505711 | 0.026027389 | 0.213502919 | Up |
| MLK7-AS1 | 1.485343463 | 0.026125346 | 0.213533899 | Up |
| XLOC_009122 | 1.012851216 | 0.026129352 | 0.213533899 | Up |
| SPECC1 | -1.036444111 | 0.026154353 | 0.213533899 | Down |
| CEACAM19 | 1.193680863 | 0.026168245 | 0.213533899 | Up |
| TAGLN | -1.646565013 | 0.02619201 | 0.213533899 | Down |
| XLOC_010997 | 1.176093084 | 0.02619728 | 0.213533899 | Up |
| CDC42BPA | -1.387768814 | 0.026200016 | 0.213533899 | Down |
| XLOC_002233 | 1.293530234 | 0.02625895 | 0.213533899 | Up |
| NUPR1 | -1.048328014 | 0.026279648 | 0.213533899 | Down |
| XLOC_005777 | 1.309671816 | 0.026309215 | 0.213533899 | Up |
| SNORD115-32 | 1.132097644 | 0.02631609 | 0.213533899 | Up |
| RPL4 | -1.077710757 | 0.026316305 | 0.213533899 | Down |
| XLOC_001719 | 1.540540754 | 0.026316499 | 0.213533899 | Up |
| STEAP2 | -1.076899478 | 0.02632058 | 0.213533899 | Down |
| CENPM | 1.215094262 | 0.026328755 | 0.213533899 | Up |
| KIAA1486 | 1.073449644 | 0.026336175 | 0.213533899 | Up |
| LEAP2 | -1.687949345 | 0.02635 | 0.213533899 | Down |
| XLOC_l2_003666 | 1.314951503 | 0.026354508 | 0.213533899 | Up |
| PELO | -1.030275289 | 0.02635637 | 0.213533899 | Down |
| LMCD1 | -1.602079321 | 0.026446927 | 0.21372816 | Down |
| XLOC_000832 | 1.053372166 | 0.026465414 | 0.213764017 | Up |
| XLOC_004124 | 1.522943194 | 0.026489297 | 0.213888802 | Up |
| TNKS | -1.010183202 | 0.026535981 | 0.213888802 | Down |
| PRSS58 | 1.228033413 | 0.026555626 | 0.213888802 | Up |
| ADNP | -1.095001946 | 0.02659171 | 0.213888802 | Down |
| XLOC_008560 | 1.088913892 | 0.026600352 | 0.213888802 | Up |
| FOXP2 | -1.272732954 | 0.026652482 | 0.213888802 | Down |
| PRSS3 | 1.136106616 | 0.026666993 | 0.213888802 | Up |
| LRRC32 | -1.551658455 | 0.026674229 | 0.213888802 | Down |
| AMBRA1 | -1.187409834 | 0.026674678 | 0.213888802 | Down |
| SP8 | 1.32447953 | 0.026677232 | 0.213888802 | Up |
| ZNF594 | -1.09943693 | 0.026685027 | 0.213888802 | Down |
| TBCE | -1.011808917 | 0.026688904 | 0.213888802 | Down |
| CEP128 | -1.022435386 | 0.026746001 | 0.214126746 | Down |
| EIF3IP1 | 1.133482553 | 0.026766571 | 0.214126746 | Up |
| XLOC_l2_014217 | 1.291264197 | 0.026768017 | 0.214126746 | Up |
| XLOC_009498 | 1.275387761 | 0.026820315 | 0.214449128 | Up |
| PDGFRB | -1.679556945 | 0.026825626 | 0.214449128 | Down |
| SNORD38A | -1.007319549 | 0.02686574 | 0.214492127 | Down |
| CCDC111 | -1.120353536 | 0.026868125 | 0.214492127 | Down |
| XLOC_003965 | 1.164724267 | 0.026900791 | 0.214618874 | Up |
| EPN2 | 1.324289298 | 0.02693707 | 0.214618874 | Up |
| SLC7A8 | -1.062426081 | 0.026944578 | 0.214618874 | Down |
| TXNDC8 | 1.311819726 | 0.026963609 | 0.214618874 | Up |
| XLOC_002772 | -1.513884156 | 0.02696539 | 0.214618874 | Down |
| KRTAP3-2 | 1.129681462 | 0.026994222 | 0.214666627 | Up |
| XLOC_014196 | 1.252197209 | 0.027005313 | 0.214666627 | Up |
| ATP5F1 | -1.027966469 | 0.027023728 | 0.214666627 | Down |
| FAM122A | -1.175607234 | 0.027035204 | 0.214698759 | Down |
| SDPR | -1.063488001 | 0.027074384 | 0.214856117 | Down |
| ERGIC1 | -1.02402279 | 0.027093242 | 0.214856117 | Down |
| BTG2 | -1.191226587 | 0.027102135 | 0.214856117 | Down |
| FHOD3 | -1.309776559 | 0.027158498 | 0.214901736 | Down |
| C20orf194 | -1.101391786 | 0.02718801 | 0.214901736 | Down |
| XLOC_003988 | 1.088306755 | 0.027211921 | 0.214901736 | Up |
| GLTPD1 | 1.124805578 | 0.027255416 | 0.214901736 | Up |
| XLOC_000974 | 1.165222106 | 0.027268272 | 0.214901736 | Up |
| NUP210 | -1.007339849 | 0.027268346 | 0.214901736 | Down |
| HLF | -1.847322183 | 0.027289495 | 0.214901736 | Down |
| XLOC_001676 | 1.459635975 | 0.027306233 | 0.214901736 | Up |
| PPWD1 | -1.079632433 | 0.027338013 | 0.214901736 | Down |
| XLOC_002987 | 2.227506572 | 0.027345866 | 0.214901736 | Up |
| ACVR1 | -1.442992924 | 0.02738072 | 0.214901736 | Down |
| XLOC_008667 | 1.791698768 | 0.02738161 | 0.214901736 | Up |
| MESDC2 | -1.075652779 | 0.027382817 | 0.214901736 | Down |
| XLOC_003650 | 1.090304813 | 0.027388954 | 0.214901736 | Up |
| CD200 | -1.088364734 | 0.027427669 | 0.214901736 | Down |
| LILRB1 | -1.03328798 | 0.027431574 | 0.214901736 | Down |
| C1orf54 | -1.028320678 | 0.027447376 | 0.214901736 | Down |
| GNB1 | -1.330100793 | 0.027450775 | 0.214901736 | Down |
| XLOC_002845 | 1.293477393 | 0.027455423 | 0.214901736 | Up |
| NEBL | -1.201767244 | 0.027463215 | 0.214901736 | Down |
| XLOC_000772 | 1.06153503 | 0.027477312 | 0.214901736 | Up |
| XLOC_012383 | 1.107052081 | 0.027500548 | 0.214934173 | Up |
| COL4A5 | -1.278864069 | 0.02752619 | 0.214934173 | Down |
| S1PR3 | -2.033413941 | 0.027562503 | 0.214934173 | Down |
| PTRHD1 | -1.287481313 | 0.027576384 | 0.214934173 | Down |
| FKBP10 | -1.399391243 | 0.027588623 | 0.214934173 | Down |
| SLC24A3 | -1.927998066 | 0.027605053 | 0.214934173 | Down |
| XLOC_004272 | 1.152819788 | 0.027606323 | 0.214934173 | Up |
| PPP1R12B | -1.400899703 | 0.027613239 | 0.214934173 | Down |
| EID3 | -1.122565943 | 0.027648921 | 0.214996342 | Down |
| ZNF189 | -1.161735301 | 0.02769041 | 0.214996943 | Down |
| NR3C2 | -1.090011231 | 0.027723382 | 0.215137316 | Down |
| FLJ31958 | 1.187655418 | 0.027756077 | 0.215235016 | Up |
| XLOC_000667 | 1.194399938 | 0.027768916 | 0.215235016 | Up |
| APOE | -1.33016178 | 0.027819418 | 0.215235016 | Down |
| USP7 | -1.04184955 | 0.027831423 | 0.215235016 | Down |
| XLOC_009736 | 1.119632288 | 0.02789116 | 0.2154109 | Up |
| SNORD64 | -1.027236377 | 0.027926242 | 0.215421379 | Down |
| EPB41L2 | -1.047375122 | 0.027927411 | 0.215421379 | Down |
| INO80E | -1.100270164 | 0.027934364 | 0.215421379 | Down |
| XLOC_005480 | 1.04356033 | 0.02793894 | 0.215421379 | Up |
| XLOC_006704 | 1.238295684 | 0.027995085 | 0.215568643 | Up |
| XLOC_006618 | 1.292183083 | 0.028016494 | 0.2155699 | Up |
| SEMA6C | 1.099683389 | 0.028081374 | 0.2155699 | Up |
| SNORA32 | -1.145442506 | 0.028084804 | 0.2155699 | Down |
| TIMP3 | -1.718168161 | 0.028121721 | 0.2155699 | Down |
| XLOC_l2_014820 | 1.305073066 | 0.028129571 | 0.2155699 | Up |
| SOCS4 | -1.0119389 | 0.028140047 | 0.2155699 | Down |
| GLB1L2 | 1.160026804 | 0.028199534 | 0.2155699 | Up |
| SYVN1 | -1.119982702 | 0.028203728 | 0.2155699 | Down |
| GGT8P | 2.103256125 | 0.028207284 | 0.2155699 | Up |
| XLOC_009643 | 1.108112473 | 0.028257592 | 0.215690908 | Up |
| PLCB2 | -1.215742009 | 0.028281709 | 0.215760987 | Down |
| RGMA | -1.067141011 | 0.028297323 | 0.215823112 | Down |
| NR1H3 | -1.061969767 | 0.02831971 | 0.215879882 | Down |
| ZNF423 | -1.260455552 | 0.028407554 | 0.216218476 | Down |
| XLOC_007842 | 1.332834663 | 0.028432725 | 0.216218476 | Up |
| DCLK3 | 1.555911214 | 0.02850991 | 0.216296918 | Up |
| XLOC_009325 | 1.078169697 | 0.028516008 | 0.216296918 | Up |
| XLOC_004230 | 1.102631217 | 0.028548463 | 0.216471366 | Up |
| XLOC_013822 | 1.458328479 | 0.028554649 | 0.216471366 | Up |
| XLOC_006276 | 1.058504029 | 0.028586386 | 0.216521771 | Up |
| XLOC_l2_010936 | 1.130517091 | 0.02859064 | 0.216521771 | Up |
| MBOAT2 | -1.081356833 | 0.028611251 | 0.216590024 | Down |
| XLOC_006532 | 1.471784025 | 0.02861587 | 0.216590024 | Up |
| ABCC6P1 | 1.042931651 | 0.028623013 | 0.216590024 | Up |
| CYCS | -1.018264199 | 0.028683771 | 0.216787126 | Down |
| CDKN1C | -1.451618751 | 0.028722928 | 0.216787126 | Down |
| XLOC_l2_012983 | 1.489974352 | 0.028730511 | 0.216787126 | Up |
| ARL15 | -1.094947883 | 0.028738688 | 0.216787126 | Down |
| IFT46 | -1.14367138 | 0.028748235 | 0.216787126 | Down |
| SPEM1 | 1.115469416 | 0.02875094 | 0.216787126 | Up |
| CRIM1 | -1.456382209 | 0.028833353 | 0.216787126 | Down |
| XLOC_003211 | 1.008633127 | 0.02884117 | 0.216787126 | Up |
| XLOC_013544 | 1.032325218 | 0.028866321 | 0.216787126 | Up |
| TP53TG3 | 1.426118577 | 0.028921066 | 0.216787126 | Up |
| P39188 | 1.26839961 | 0.028921825 | 0.216787126 | Up |
| XLOC_005051 | 1.112704877 | 0.028950449 | 0.216787126 | Up |
| H1FX | -1.020623476 | 0.028954966 | 0.216787126 | Down |
| FAM179B | -1.049319316 | 0.028966842 | 0.216787126 | Down |
| XLOC_l2_010511 | 1.278151238 | 0.028968879 | 0.216787126 | Up |
| SEC63 | -1.006509582 | 0.028977323 | 0.216787126 | Down |
| XLOC_014029 | 1.448264395 | 0.029016669 | 0.216787126 | Up |
| PDE7B | -1.493608228 | 0.029036501 | 0.216787126 | Down |
| SNUPN | -1.004660889 | 0.029070006 | 0.216906391 | Down |
| XLOC_002887 | 1.163059592 | 0.029158076 | 0.217157634 | Up |
| KRT34 | 1.804361263 | 0.029196635 | 0.217210342 | Up |
| XLOC_010305 | 1.171264273 | 0.029207201 | 0.217210342 | Up |
| CD244 | 1.624580739 | 0.029333386 | 0.217231295 | Up |
| GPRASP1 | -1.385733119 | 0.029377654 | 0.217231295 | Down |
| NFIA | -1.381316858 | 0.029379649 | 0.217231295 | Down |
| ZNF618 | -1.074214846 | 0.02938356 | 0.217231295 | Down |
| XLOC_012322 | 1.126511149 | 0.029422542 | 0.217231295 | Up |
| XLOC_001645 | 1.319470268 | 0.029440163 | 0.21725562 | Up |
| LOH12CR1 | -1.034011436 | 0.029468484 | 0.217409082 | Down |
| SCN1A | 1.180169688 | 0.029527854 | 0.217510951 | Up |
| THSD4 | -1.103496563 | 0.029541639 | 0.217510951 | Down |
| SPAG6 | 1.568387454 | 0.029550299 | 0.217510951 | Up |
| FLNB | -1.088203886 | 0.029556885 | 0.217510951 | Down |
| NOSIP | -1.157069398 | 0.029575905 | 0.217510951 | Down |
| PRDM12 | 1.035953676 | 0.029588496 | 0.217510951 | Up |
| C2orf62 | 1.013074166 | 0.029634969 | 0.21755423 | Up |
| XLOC_008653 | 1.263670632 | 0.029710607 | 0.217595445 | Up |
| XLOC_008539 | 1.013632395 | 0.029727224 | 0.217595445 | Up |
| XLOC_009838 | 1.638985261 | 0.029743599 | 0.21760831 | Up |
| PCDH11X | 1.019237158 | 0.029757514 | 0.217631848 | Up |
| EIF3C | -1.006164203 | 0.029772629 | 0.217652083 | Down |
| XLOC_000056 | 1.07424891 | 0.029805506 | 0.217682121 | Up |
| CRX | 1.536733825 | 0.029819701 | 0.217682121 | Up |
| XLOC_010840 | 1.027442865 | 0.029827793 | 0.217682121 | Up |
| FBXL19 | 1.178522372 | 0.029829298 | 0.217682121 | Up |
| MIR7-3HG | 1.849396789 | 0.029881796 | 0.217753375 | Up |
| BIK | -1.223544591 | 0.029897394 | 0.217753375 | Down |
| MAPK8IP3 | -1.002197123 | 0.029900859 | 0.217753375 | Down |
| ZNF711 | -1.134388259 | 0.029932314 | 0.217753375 | Down |
| SLC6A20 | 1.672958873 | 0.029934028 | 0.217753375 | Up |
| OVCA2 | -1.040672548 | 0.029946722 | 0.217753375 | Down |
| KIR2DL2 | 2.086627296 | 0.029961119 | 0.217753375 | Up |
| LIPF | 1.039842817 | 0.030001859 | 0.217894345 | Up |
| SMAD2 | -1.029185566 | 0.030054187 | 0.218116651 | Down |
| EFEMP1 | -1.394549779 | 0.030113117 | 0.218116651 | Down |
| MAGOH | -1.252740721 | 0.030117995 | 0.218116651 | Down |
| RPL23P8 | -1.098960617 | 0.03013592 | 0.218116651 | Down |
| PLEKHH1 | -1.291074471 | 0.030174417 | 0.218180025 | Down |
| XLOC_012041 | 1.588679384 | 0.030211196 | 0.218193892 | Up |
| RG9MTD3 | -1.040604276 | 0.030216807 | 0.218193892 | Down |
| XLOC_002924 | 1.0976792 | 0.030248126 | 0.218307015 | Up |
| XLOC_005306 | 1.190039937 | 0.030319912 | 0.218435431 | Up |
| XLOC_008456 | 1.126533285 | 0.030329258 | 0.218435431 | Up |
| LMF2 | 1.088749943 | 0.030341069 | 0.218435431 | Up |
| XLOC_l2_007542 | 1.194073887 | 0.030352538 | 0.218435431 | Up |
| RAB8B | -1.265314125 | 0.030417244 | 0.218537986 | Down |
| PDZRN4 | -1.041996401 | 0.030437075 | 0.218537986 | Down |
| C19orf51 | 1.219919579 | 0.030438436 | 0.218537986 | Up |
| TUB | -1.190501213 | 0.030468512 | 0.218590954 | Down |
| XLOC_001680 | 1.255914663 | 0.030486059 | 0.218621601 | Up |
| TRIM32 | -1.071931092 | 0.030557041 | 0.219008553 | Down |
| TCF4 | -1.134008669 | 0.030577072 | 0.21904344 | Down |
| XLOC_l2_003391 | 1.59724643 | 0.030626628 | 0.219136741 | Up |
| XLOC_l2_004611 | 2.249817829 | 0.030677417 | 0.219136741 | Up |
| WFDC10A | 1.596343133 | 0.030708772 | 0.219136741 | Up |
| XLOC_004530 | 1.224194456 | 0.030723931 | 0.219136741 | Up |
| TOMM6 | -1.004521091 | 0.030746581 | 0.219136741 | Down |
| SLC38A5 | 1.163562015 | 0.030753744 | 0.219136741 | Up |
| DNAH9 | 1.106138914 | 0.030780955 | 0.219136741 | Up |
| PRAMEF4 | 1.345490558 | 0.030785085 | 0.219136741 | Up |
| XLOC_011929 | 1.418086089 | 0.030808369 | 0.219136741 | Up |
| XLOC_004691 | 2.148065277 | 0.030812474 | 0.219136741 | Up |
| NCAM1 | -1.369379272 | 0.030820397 | 0.219136741 | Down |
| NUDT6 | -1.123865551 | 0.030861078 | 0.219136741 | Down |
| HPGDS | -1.095177792 | 0.030871244 | 0.219136741 | Down |
| FLJ34747 | 1.135002915 | 0.030894004 | 0.219136741 | Up |
| RPL29 | -1.207082926 | 0.030913277 | 0.219136741 | Down |
| C21orf2 | -1.110150065 | 0.030965134 | 0.219201704 | Down |
| SLN | -1.295151248 | 0.03097304 | 0.219201704 | Down |
| XLOC_008372 | 1.650123267 | 0.031103913 | 0.219435865 | Up |
| RPL18 | -1.021452741 | 0.031128217 | 0.219435865 | Down |
| FCER1A | -1.222888518 | 0.031140916 | 0.219435865 | Down |
| XLOC_009765 | 1.083683997 | 0.03114915 | 0.219435865 | Up |
| DIS3L | -1.118814533 | 0.031163675 | 0.219435865 | Down |
| CUL4B | -1.009195915 | 0.031182735 | 0.219435865 | Down |
| PRAMEF16 | 1.78443224 | 0.031229155 | 0.219435865 | Up |
| XLOC_013804 | 1.049230508 | 0.031320293 | 0.219435865 | Up |
| WLS | -1.21431756 | 0.031340466 | 0.219435865 | Down |
| DNMBP-AS1 | 1.074341239 | 0.031362391 | 0.219435865 | Up |
| XLOC_013792 | 1.872131054 | 0.03139361 | 0.219435865 | Up |
| CWF19L2 | -1.017674564 | 0.031406651 | 0.219435865 | Down |
| NR2F6 | -1.005364362 | 0.031418964 | 0.219435865 | Down |
| XLOC_001873 | 1.482053109 | 0.031469522 | 0.219435865 | Up |
| OR6C3 | 1.065908755 | 0.031471353 | 0.219435865 | Up |
| ZNF146 | -1.087813362 | 0.031492387 | 0.219435865 | Down |
| TRAF1 | -1.109849157 | 0.031512489 | 0.219435865 | Down |
| SEPT7P2 | -1.146666239 | 0.031512959 | 0.219435865 | Down |
| XLOC_012683 | 1.283836563 | 0.031519366 | 0.219435865 | Up |
| SCGB3A2 | 1.283812567 | 0.031583911 | 0.219435865 | Up |
| XLOC_009797 | -1.047555469 | 0.031589244 | 0.219435865 | Down |
| FAM123C | 1.388376675 | 0.031610266 | 0.219435865 | Up |
| XLOC_008185 | -3.607621249 | 0.031651109 | 0.219435865 | Down |
| XLOC_011096 | 1.009332444 | 0.031655803 | 0.219435865 | Up |
| IL25 | 1.312789729 | 0.031657218 | 0.219435865 | Up |
| KRT33A | 1.094520453 | 0.031688549 | 0.219497825 | Up |
| XLOC_009781 | 1.019096238 | 0.031732686 | 0.219695434 | Up |
| TMEM200B | -1.4277046 | 0.031765177 | 0.21972288 | Down |
| XLOC_l2_006196 | 1.700719407 | 0.031771502 | 0.21972288 | Up |
| ACSS2 | -1.186450628 | 0.031775957 | 0.21972288 | Down |
| MBLAC2 | -1.051181325 | 0.031782547 | 0.21972288 | Down |
| PSG2 | 1.244657689 | 0.031802652 | 0.21972288 | Up |
| XLOC_l2_010697 | 1.253395921 | 0.031881771 | 0.219910211 | Up |
| HMP19 | 1.361759416 | 0.03189542 | 0.219910211 | Up |
| SEC31A | -1.023741542 | 0.031907128 | 0.219910211 | Down |
| KLK4 | 1.111507732 | 0.031920032 | 0.219910211 | Up |
| LPP-AS2 | 1.09800211 | 0.031935178 | 0.219910211 | Up |
| PLK1S1 | -1.271915178 | 0.031969407 | 0.219913043 | Down |
| GOLGA8A | -1.037102365 | 0.032010941 | 0.220039991 | Down |
| P2RY14 | -1.632034955 | 0.032023005 | 0.220049979 | Down |
| SOX18 | -1.066121248 | 0.032060852 | 0.220049979 | Down |
| TTTY17A | 1.225908057 | 0.032076128 | 0.220049979 | Up |
| XLOC_l2_014785 | -1.344139329 | 0.032134896 | 0.220210929 | Down |
| SLC29A1 | -1.119225348 | 0.032161818 | 0.220238703 | Down |
| ARL4C | -1.272712272 | 0.032194846 | 0.220256046 | Down |
| ATP5C1 | -1.088532535 | 0.032219973 | 0.220262069 | Down |
| XLOC_007866 | 1.485998343 | 0.03222358 | 0.220262069 | Up |
| XLOC_006681 | 1.608097314 | 0.03224999 | 0.220262069 | Up |
| CYP2A13 | 1.001725316 | 0.032259855 | 0.220262069 | Up |
| XLOC_l2_014245 | 1.514053421 | 0.032280643 | 0.220262069 | Up |
| SYNJ2BP | -1.013070892 | 0.032301405 | 0.220262069 | Down |
| XK | -1.198381318 | 0.032307001 | 0.220262069 | Down |
| XLOC_007368 | 1.1628209 | 0.032311936 | 0.220262069 | Up |
| XLOC_007917 | 1.301244348 | 0.032405171 | 0.220463839 | Up |
| NFE4 | 1.844349361 | 0.032448901 | 0.220536553 | Up |
| XLOC_000043 | 1.067075714 | 0.03248201 | 0.220536553 | Up |
| FAM26D | 1.155876286 | 0.032525917 | 0.220536553 | Up |
| XLOC_013553 | 1.482876047 | 0.032536929 | 0.220559461 | Up |
| ALDH1A2 | -1.281135468 | 0.032583764 | 0.220677854 | Down |
| OR2T8 | 1.93068018 | 0.032584079 | 0.220677854 | Up |
| KCNK16 | 1.232160393 | 0.032617836 | 0.220693652 | Up |
| ATP5A1 | -1.024451105 | 0.032832817 | 0.220734885 | Down |
| STOM | -1.138664057 | 0.032845935 | 0.220734885 | Down |
| KIAA0895L | -1.445334625 | 0.03284734 | 0.220734885 | Down |
| XLOC_009762 | 1.079320037 | 0.032885035 | 0.220734885 | Up |
| OR56B1 | 1.159913952 | 0.032920984 | 0.220734885 | Up |
| XLOC_l2_007569 | 1.018082448 | 0.032929852 | 0.220734885 | Up |
| CCNDBP1 | -1.069239395 | 0.032962671 | 0.220734885 | Down |
| XLOC_l2_005503 | 1.474419152 | 0.032987217 | 0.220734885 | Up |
| PITX2 | 1.535080986 | 0.033020145 | 0.220734885 | Up |
| TMEM56-RWDD3 | -1.208311904 | 0.033027289 | 0.220734885 | Down |
| ENOSF1 | -1.008094879 | 0.033028795 | 0.220734885 | Down |
| XLOC_001922 | -1.055195736 | 0.033083505 | 0.220871033 | Down |
| XLOC_004312 | 1.062694998 | 0.033087461 | 0.220871033 | Up |
| XLOC_l2_009136 | -1.041122877 | 0.033119269 | 0.220954983 | Down |
| XLOC_001309 | 1.300295484 | 0.033148183 | 0.220954983 | Up |
| XLOC_012792 | 1.186921856 | 0.033149614 | 0.220954983 | Up |
| MATN2 | -1.426612235 | 0.033188478 | 0.220954983 | Down |
| ZNF91 | -1.021027408 | 0.033202702 | 0.220954983 | Down |
| TCF12 | -1.303481419 | 0.033210198 | 0.220954983 | Down |
| PAPOLB | 1.129587846 | 0.033230492 | 0.220954983 | Up |
| EPHA8 | 1.362224334 | 0.033257013 | 0.220954983 | Up |
| FAM165B | 1.0394125 | 0.033284436 | 0.220954983 | Up |
| HABP4 | -1.08762326 | 0.033312166 | 0.220954983 | Down |
| XLOC_014331 | 1.073185097 | 0.033330183 | 0.220954983 | Up |
| XLOC_007301 | 1.004262472 | 0.03333024 | 0.220954983 | Up |
| XLOC_008665 | 1.366285103 | 0.033398589 | 0.220954983 | Up |
| LRMP | -1.292133727 | 0.03339889 | 0.220954983 | Down |
| XLOC_007390 | 1.532781883 | 0.03347849 | 0.220954983 | Up |
| DEFB116 | 1.292669662 | 0.033482431 | 0.220954983 | Up |
| CYP2A7 | 2.039299499 | 0.033494482 | 0.220984032 | Up |
| XLOC_013617 | 1.347663142 | 0.033521111 | 0.221001381 | Up |
| RPL10A | -1.047952847 | 0.033561387 | 0.221001381 | Down |
| OR9Q2 | 1.063445671 | 0.033611706 | 0.221001381 | Up |
| Q4TAJ0 | 1.337192516 | 0.033659155 | 0.221001381 | Up |
| ONECUT2 | 1.398178472 | 0.033710755 | 0.221001381 | Up |
| CSRP1 | -1.210116855 | 0.033737102 | 0.221001381 | Down |
| OR2J2 | 1.299304217 | 0.033739192 | 0.221001381 | Up |
| XLOC_003281 | 1.118552234 | 0.033770002 | 0.221001381 | Up |
| XLOC_003710 | 1.043393738 | 0.033773564 | 0.221001381 | Up |
| TET2 | -1.106925765 | 0.03389485 | 0.221195549 | Down |
| NLE1 | 1.017641663 | 0.033917008 | 0.221195549 | Up |
| XLOC_000882 | 1.033547269 | 0.033965827 | 0.221242949 | Up |
| DYNC1LI2 | -1.107716033 | 0.033968133 | 0.221242949 | Down |
| SCG3 | 1.263573415 | 0.033980301 | 0.221242949 | Up |
| ERGIC3 | -1.141738334 | 0.03399288 | 0.221242949 | Down |
| C14orf166B | 1.293560984 | 0.034017181 | 0.221242949 | Up |
| CSTA | -1.524592674 | 0.034018634 | 0.221242949 | Down |
| RNASE1 | -1.569939893 | 0.034045096 | 0.221242949 | Down |
| FN3KRP | -1.007113045 | 0.034100448 | 0.221242949 | Down |
| ZNF17 | -1.111586798 | 0.034118352 | 0.221242949 | Down |
| XLOC_013778 | 2.171939339 | 0.034123594 | 0.221242949 | Up |
| RPF1 | 1.199458097 | 0.03414944 | 0.221242949 | Up |
| XLOC_005114 | 1.399216617 | 0.034166181 | 0.221242949 | Up |
| SFMBT1 | -1.194122498 | 0.034175578 | 0.221242949 | Down |
| SLC47A1 | -1.315314408 | 0.034193049 | 0.221242949 | Down |
| XLOC_010674 | 1.035367713 | 0.034202764 | 0.221242949 | Up |
| XLOC_002007 | 1.243703207 | 0.034271418 | 0.221242949 | Up |
| XLOC_007118 | 2.014579744 | 0.034276537 | 0.221242949 | Up |
| XLOC_010445 | 1.172070145 | 0.034323287 | 0.221445755 | Up |
| REEP1 | -1.324823474 | 0.034363343 | 0.221646617 | Down |
| SYCP1 | 1.099648995 | 0.034388786 | 0.22167033 | Up |
| XLOC_005475 | 1.524921853 | 0.034439745 | 0.221720603 | Up |
| XLOC_006422 | 1.272971846 | 0.034445999 | 0.221720603 | Up |
| XLOC_000293 | 1.155696845 | 0.034455034 | 0.221720603 | Up |
| EMX1 | 1.044962995 | 0.034518664 | 0.221720603 | Up |
| ZNF621 | -1.143189657 | 0.034529563 | 0.221720603 | Down |
| ERVW-1 | 1.041113819 | 0.034586034 | 0.221720603 | Up |
| TSPY2 | 1.257072105 | 0.034592751 | 0.221720603 | Up |
| UBR7 | -1.282142188 | 0.034640877 | 0.221720603 | Down |
| GUSBP1 | 1.751910561 | 0.034642345 | 0.221720603 | Up |
| BTG4 | 1.611547448 | 0.034847638 | 0.221720603 | Up |
| CD80 | 1.099608485 | 0.034884903 | 0.221720603 | Up |
| PRKX | -1.45394412 | 0.034923878 | 0.221720603 | Down |
| XLOC_l2_015752 | 1.164848471 | 0.034926818 | 0.221720603 | Up |
| SCHIP1 | -1.242111579 | 0.034950621 | 0.221720603 | Down |
| ELOVL5 | -1.187984511 | 0.034951954 | 0.221720603 | Down |
| XLOC_000184 | 1.602514153 | 0.035007437 | 0.221720603 | Up |
| ZFYVE16 | -1.062551966 | 0.035012367 | 0.221720603 | Down |
| SNHG8 | -1.229755176 | 0.035023621 | 0.221720603 | Down |
| OR6P1 | 1.209162871 | 0.035032133 | 0.221720603 | Up |
| XLOC_009873 | 1.293596273 | 0.035044639 | 0.221720603 | Up |
| GSTTP2 | 1.063100011 | 0.035052969 | 0.221720603 | Up |
| OSGEPL1 | -1.018874983 | 0.035088944 | 0.221720603 | Down |
| CD99 | -1.189845536 | 0.035089834 | 0.221720603 | Down |
| XLOC_007640 | 2.247246102 | 0.035097802 | 0.221720603 | Up |
| CCND2 | -1.258601977 | 0.035143473 | 0.221720603 | Down |
| FLJ39739 | 1.247809963 | 0.035154206 | 0.221720603 | Up |
| XLOC_012858 | 1.011071606 | 0.035185328 | 0.221720603 | Up |
| SPATS2L | -1.139442017 | 0.03518581 | 0.221720603 | Down |
| FBXO21 | -1.087130621 | 0.035190638 | 0.221720603 | Down |
| TNRC6A | -1.250982651 | 0.035203115 | 0.221725138 | Down |
| TAS2R7 | 1.138335827 | 0.035290145 | 0.222128015 | Up |
| C4orf52 | -1.093051626 | 0.035348292 | 0.222293353 | Down |
| EIF4EBP2 | -1.088862542 | 0.035369005 | 0.222333751 | Down |
| WFDC12 | 1.016242735 | 0.03539362 | 0.222368913 | Up |
| XLOC_005284 | 1.071084527 | 0.03540324 | 0.222368913 | Up |
| ZNF878 | -1.144572613 | 0.035423823 | 0.222376712 | Down |
| XLOC_001124 | 1.423006354 | 0.035460651 | 0.222376712 | Up |
| RRAGB | -1.13682994 | 0.035463209 | 0.222376712 | Down |
| TRIM66 | -1.013723533 | 0.035473593 | 0.222376712 | Down |
| TACR2 | 1.190031887 | 0.035477775 | 0.222376712 | Up |
| XLOC_004640 | 1.125764237 | 0.035483598 | 0.222376712 | Up |
| SPIN4 | -1.212928991 | 0.035572481 | 0.222591062 | Down |
| XLOC_005136 | 1.153439205 | 0.035573078 | 0.222591062 | Up |
| SLC17A3 | 1.138174079 | 0.035581831 | 0.222591062 | Up |
| PCOLCE2 | -1.325782377 | 0.035620635 | 0.222591062 | Down |
| SLC25A32 | -1.030944603 | 0.035719352 | 0.222900419 | Down |
| NTN1 | -1.112327037 | 0.035734643 | 0.222900419 | Down |
| MRPS18A | -1.104914439 | 0.035750419 | 0.222900419 | Down |
| DEFB119 | 1.02482858 | 0.035766347 | 0.222900419 | Up |
| SRPX | -1.526172007 | 0.035783251 | 0.222900419 | Down |
| ABI2 | -1.009917354 | 0.035819132 | 0.222900419 | Down |
| TEDDM1 | 1.096744293 | 0.035844156 | 0.222900419 | Up |
| PLN | -1.438893569 | 0.035849764 | 0.222900419 | Down |
| CBR1 | 1.048320915 | 0.035940692 | 0.222989741 | Up |
| PPP1R2P3 | 1.149020962 | 0.036000041 | 0.222989741 | Up |
| LRIT2 | 1.500434803 | 0.036017746 | 0.222989741 | Up |
| C14orf43 | -1.165840899 | 0.036067252 | 0.222989741 | Down |
| REG4 | 1.072012022 | 0.036068882 | 0.222989741 | Up |
| SUSD3 | -1.15217266 | 0.036085203 | 0.222989741 | Down |
| FAM172A | -1.064238187 | 0.036101411 | 0.222989741 | Down |
| XLOC_009680 | 1.101184651 | 0.036114327 | 0.222989741 | Up |
| ANKRD30BP2 | 1.173056151 | 0.036125689 | 0.222989741 | Up |
| ZHX1 | -1.227034899 | 0.036147464 | 0.222989741 | Down |
| SPAG11B | 1.155707371 | 0.036192742 | 0.223062627 | Up |
| DUXA | 1.127040713 | 0.036280798 | 0.22326098 | Up |
| TRIM59 | -1.227261721 | 0.036288464 | 0.22326098 | Down |
| SOHLH2 | -1.193631196 | 0.036331136 | 0.223301091 | Down |
| XLOC_008451 | 1.002141751 | 0.036357834 | 0.223353704 | Up |
| XLOC_007646 | 1.155930176 | 0.036390239 | 0.223396321 | Up |
| LPPR4 | -1.269224182 | 0.036402003 | 0.223396321 | Down |
| TIE1 | -1.023853466 | 0.036423877 | 0.223396321 | Down |
| FAM118A | -1.167911904 | 0.036425969 | 0.223396321 | Down |
| MRC2 | -1.490569626 | 0.036433639 | 0.223396321 | Down |
| TSPYL4 | -1.026026619 | 0.036481148 | 0.223489575 | Down |
| PDGFRA | -1.65004932 | 0.036513464 | 0.223568585 | Down |
| XLOC_012176 | 1.075552665 | 0.036562259 | 0.223568585 | Up |
| KLHL30 | -1.004160593 | 0.036564583 | 0.223568585 | Down |
| XLOC_001663 | 1.184678943 | 0.036625567 | 0.223630825 | Up |
| OR5A1 | 1.038574703 | 0.036707998 | 0.223882364 | Up |
| ZNF564 | -1.157025308 | 0.036753965 | 0.223979668 | Down |
| KCNIP4 | -1.648460432 | 0.036754962 | 0.223979668 | Down |
| POT1 | -1.077314402 | 0.036780168 | 0.223991535 | Down |
| IFI27 | -1.581592866 | 0.036812489 | 0.224031874 | Down |
| TGFBR3 | -1.014404314 | 0.036828414 | 0.224031874 | Down |
| XLOC_011368 | -1.514699778 | 0.03686547 | 0.224097543 | Down |
| XLOC_004269 | 1.169218489 | 0.036869564 | 0.224097543 | Up |
| MFAP4 | -1.873357728 | 0.03695301 | 0.224282141 | Down |
| CDC14A | -1.059925811 | 0.037024418 | 0.224282141 | Down |
| PARD6G | -1.194916825 | 0.03702593 | 0.224282141 | Down |
| XLOC_005675 | 1.171190778 | 0.037101082 | 0.224282141 | Up |
| XLOC_002863 | 1.128482977 | 0.037140955 | 0.224282141 | Up |
| WDR63 | 1.069909324 | 0.037144223 | 0.224282141 | Up |
| C21orf15 | -1.291426617 | 0.03715706 | 0.224282141 | Down |
| XLOC_002380 | 1.091704234 | 0.037167583 | 0.224282141 | Up |
| PKN3 | -1.199191761 | 0.037214293 | 0.224282141 | Down |
| XLOC_002517 | 1.05366976 | 0.037214967 | 0.224282141 | Up |
| PDCD2L | -1.161838456 | 0.037241116 | 0.224282141 | Down |
| XLOC_013777 | 1.188811277 | 0.037284061 | 0.224282141 | Up |
| XLOC_003441 | 1.111150381 | 0.037285192 | 0.224282141 | Up |
| SCARA5 | -1.365148495 | 0.037331782 | 0.224282141 | Down |
| DLD | -1.110527816 | 0.037340831 | 0.224282141 | Down |
| FLJ36000 | 1.301629703 | 0.037381674 | 0.224282141 | Up |
| MAPK10 | -1.05002417 | 0.037395727 | 0.224282141 | Down |
| HOXA3 | -1.480030339 | 0.037405205 | 0.224282141 | Down |
| XLOC_013494 | 1.007352677 | 0.037431761 | 0.224282141 | Up |
| XLOC_006390 | -1.051887823 | 0.037450001 | 0.224282141 | Down |
| XLOC_001638 | 1.087586914 | 0.0375075 | 0.224282141 | Up |
| RASL10B | 1.277861225 | 0.037591557 | 0.224293318 | Up |
| C9orf3 | -1.453688323 | 0.037606706 | 0.224293318 | Down |
| ID1 | -1.120932923 | 0.037613829 | 0.224293318 | Down |
| NFAT5 | -1.06338412 | 0.037700831 | 0.224474367 | Down |
| XLOC_013504 | 1.318330337 | 0.037706348 | 0.224474367 | Up |
| GTSF1L | 1.225726873 | 0.037741714 | 0.224514141 | Up |
| XLOC_002401 | 1.163769945 | 0.037753429 | 0.224523326 | Up |
| RPS18 | -1.2198059 | 0.037794491 | 0.22466278 | Down |
| HPVC1 | 1.395114443 | 0.037808256 | 0.22466278 | Up |
| HIST1H1D | -1.055798422 | 0.037819936 | 0.22466278 | Down |
| XLOC_008447 | 1.210355281 | 0.03786206 | 0.22466278 | Up |
| C6orf57 | -1.167880222 | 0.037906121 | 0.22466278 | Down |
| XLOC_003017 | 1.51249728 | 0.037930388 | 0.22466278 | Up |
| RPL3 | -1.066643549 | 0.038087603 | 0.224896372 | Down |
| BPIFA1 | 1.017476008 | 0.038088607 | 0.224896372 | Up |
| MYADML | 1.086183419 | 0.038147464 | 0.22490182 | Up |
| XLOC_l2_001037 | 1.076620376 | 0.03817949 | 0.225019102 | Up |
| TRDN | 1.099636834 | 0.038213621 | 0.225036634 | Up |
| C6orf123 | 1.029648508 | 0.038262654 | 0.225129192 | Up |
| SYT12 | 1.222540861 | 0.038277871 | 0.22513965 | Up |
| XLOC_010216 | 1.313102668 | 0.038295729 | 0.22516132 | Up |
| XLOC_013284 | 1.085940294 | 0.038400114 | 0.225262475 | Up |
| NAB2 | -1.199193802 | 0.038436869 | 0.225358517 | Down |
| IGFL1 | 1.249503137 | 0.038439498 | 0.225358517 | Up |
| XLOC_013772 | 1.414163122 | 0.038459816 | 0.225358517 | Up |
| MFGE8 | -1.222047524 | 0.038503698 | 0.225381075 | Down |
| XLOC_010775 | 1.032479916 | 0.038552166 | 0.225467738 | Up |
| PABPC4 | -1.086969888 | 0.03858631 | 0.225530466 | Down |
| XLOC_005129 | 1.296026296 | 0.038611977 | 0.225591706 | Up |
| THNSL1 | -1.017305872 | 0.038612405 | 0.225591706 | Down |
| DCT | 1.185037311 | 0.038717219 | 0.226066932 | Up |
| TERF2 | -1.268534294 | 0.03878978 | 0.226261967 | Down |
| XLOC_006198 | 1.36720815 | 0.038925861 | 0.226623937 | Up |
| XLOC_009894 | 1.031605708 | 0.038971039 | 0.22663291 | Up |
| BRSK2 | 1.445618072 | 0.039051046 | 0.226789443 | Up |
| DDC | 1.41147988 | 0.039052282 | 0.226789443 | Up |
| USP17L2 | 2.11613315 | 0.039145492 | 0.226789443 | Up |
| BOD1L | -1.312896538 | 0.03914754 | 0.226789443 | Down |
| NPAT | -1.036539933 | 0.039180238 | 0.226789443 | Down |
| KRTAP4-7 | 1.050287622 | 0.039253826 | 0.226795553 | Up |
| PCGF3 | -1.063777234 | 0.039329902 | 0.226983661 | Down |
| KLRK1 | -1.012922776 | 0.0393866 | 0.227220099 | Down |
| XLOC_005567 | 1.103277694 | 0.039486057 | 0.227445448 | Up |
| RPS27 | 1.05657019 | 0.039540071 | 0.227445448 | Up |
| TRIO | -1.052051571 | 0.03954375 | 0.227445448 | Down |
| CHODL | -1.497761098 | 0.039569815 | 0.227459517 | Down |
| TMEM229A | 1.288752278 | 0.03963965 | 0.227671085 | Up |
| XLOC_013699 | 1.001767072 | 0.039685424 | 0.227671085 | Up |
| SBF2 | -1.093898129 | 0.03972091 | 0.227689501 | Down |
| TSPY3 | 1.130612176 | 0.039734795 | 0.227689501 | Up |
| PC | 1.198582799 | 0.039758297 | 0.227727391 | Up |
| CD40LG | -1.196121041 | 0.039787302 | 0.227758064 | Down |
| TRIM68 | -1.178706076 | 0.039835547 | 0.227763463 | Down |
| GPC4 | -1.358068965 | 0.039883166 | 0.227822902 | Down |
| CLEC14A | -1.22496044 | 0.039917139 | 0.227822902 | Down |
| TMEM38B | -1.132301842 | 0.039999004 | 0.227986696 | Down |
| Q7VPR2 | 1.046803634 | 0.040006468 | 0.227986696 | Up |
| PPM1L | -1.02108994 | 0.04007885 | 0.228116243 | Down |
| AFF3 | 1.541735445 | 0.040140084 | 0.228312933 | Up |
| IGFL4 | 1.197955553 | 0.040146859 | 0.228312933 | Up |
| DSC2 | -1.246776211 | 0.040197263 | 0.228312933 | Down |
| XLOC_001815 | 1.235087211 | 0.040285692 | 0.228312933 | Up |
| NLGN4X | -1.148295943 | 0.040299636 | 0.228312933 | Down |
| PNLIPRP3 | 1.255817395 | 0.040302531 | 0.228312933 | Up |
| ELMO1 | -1.011896187 | 0.040480597 | 0.228312933 | Down |
| ARHGAP17 | 1.006089123 | 0.040494811 | 0.228312933 | Up |
| PANX3 | 1.214737525 | 0.040560456 | 0.228312933 | Up |
| ANKRD9 | -1.001899799 | 0.040563748 | 0.228312933 | Down |
| AKR7A2 | -1.024919613 | 0.040587168 | 0.228312933 | Down |
| PADI6 | 1.223543272 | 0.040597556 | 0.228312933 | Up |
| FMO2 | -1.026891962 | 0.040695521 | 0.228312933 | Down |
| PMCHL1 | 1.16834207 | 0.040708118 | 0.228312933 | Up |
| C10orf108 | -1.012004353 | 0.040709221 | 0.228312933 | Down |
| KLHL1 | -1.041100256 | 0.040749202 | 0.228312933 | Down |
| PCDHB5 | -1.287964048 | 0.040760007 | 0.228312933 | Down |
| XLOC_l2_010926 | 1.284028472 | 0.040776995 | 0.228312933 | Up |
| IKZF4 | -1.237225275 | 0.040809298 | 0.228312933 | Down |
| XLOC_008043 | 1.049157261 | 0.04081081 | 0.228312933 | Up |
| SNORD114-26 | 1.01295469 | 0.040835215 | 0.228312933 | Up |
| CAMP | 1.084546363 | 0.040842919 | 0.228312933 | Up |
| UACA | -1.338124884 | 0.04087916 | 0.228312933 | Down |
| XLOC_004002 | 1.067327099 | 0.040888673 | 0.228312933 | Up |
| EPAS1 | -1.253866357 | 0.040894673 | 0.228312933 | Down |
| XLOC_009608 | 1.102049847 | 0.040895761 | 0.228312933 | Up |
| CLASP1 | -1.239443709 | 0.040923486 | 0.228368272 | Down |
| TTTY21 | 1.058296558 | 0.04101603 | 0.228417239 | Up |
| CTSZ | -1.259943151 | 0.041053433 | 0.228417239 | Down |
| XLOC_014134 | 1.075560757 | 0.041057875 | 0.228417239 | Up |
| LRRC47 | -1.220871368 | 0.041115127 | 0.228609916 | Down |
| KLK3 | 1.328804492 | 0.041221371 | 0.228854585 | Up |
| R3HDML | 1.097239864 | 0.041271534 | 0.22890687 | Up |
| XLOC_010284 | -1.35369845 | 0.041292353 | 0.228908333 | Down |
| XLOC_013298 | 1.05612908 | 0.041356146 | 0.228908333 | Up |
| ABCD2 | -1.013261801 | 0.041378699 | 0.228908333 | Down |
| ZFHX4 | 1.245894823 | 0.041391563 | 0.228908333 | Up |
| SERPINF1 | -1.618776545 | 0.041397619 | 0.228908333 | Down |
| XLOC_012623 | 1.040932283 | 0.04152919 | 0.229046201 | Up |
| XLOC_014326 | 1.076603887 | 0.041563901 | 0.229046201 | Up |
| ZFP30 | -1.072151093 | 0.041576722 | 0.229046201 | Down |
| XLOC_004320 | 1.208528067 | 0.041598869 | 0.229078686 | Up |
| XLOC_008402 | 1.460654223 | 0.041637501 | 0.229078686 | Up |
| ZNF529 | -1.021037888 | 0.041643874 | 0.229078686 | Down |
| PITPNB | -1.006313191 | 0.041656933 | 0.229078686 | Down |
| XLOC_011474 | 2.042883783 | 0.041712288 | 0.229078686 | Up |
| XLOC_006712 | 1.157319884 | 0.041807173 | 0.229145616 | Up |
| XLOC_000986 | 1.033604519 | 0.041807611 | 0.229145616 | Up |
| XLOC_003429 | 1.097467973 | 0.041853475 | 0.229145616 | Up |
| CXCR4 | -1.356387598 | 0.041885935 | 0.229173935 | Down |
| AASDHPPT | -1.03461145 | 0.041890816 | 0.229173935 | Down |
| FLI1 | -1.221057617 | 0.041905522 | 0.229208089 | Down |
| XLOC_011029 | 1.571726211 | 0.042002252 | 0.229469822 | Up |
| FAM170A | 1.037140875 | 0.042008611 | 0.229469822 | Up |
| SPATA6 | -1.167658241 | 0.042109932 | 0.229647053 | Down |
| CEL | 1.196025298 | 0.042143047 | 0.229647053 | Up |
| SFRP4 | -1.291371917 | 0.042169624 | 0.229650631 | Down |
| HIST1H1E | -1.215327377 | 0.042215563 | 0.229650631 | Down |
| DNAJB4 | -1.051198921 | 0.042226681 | 0.229650631 | Down |
| XLOC_012456 | 1.094579101 | 0.042274126 | 0.229802138 | Up |
| XLOC_013862 | 1.341916814 | 0.042342203 | 0.22990201 | Up |
| LINC00494 | 1.011375173 | 0.042440314 | 0.23008878 | Up |
| XLOC_l2_005179 | 1.44618078 | 0.042462137 | 0.230120729 | Up |
| PTGIS | -1.602789158 | 0.042602335 | 0.230239396 | Down |
| C9orf95 | -1.015407858 | 0.042609014 | 0.230239396 | Down |
| FLJ44313 | 1.109090668 | 0.042659278 | 0.230322101 | Up |
| OR8A1 | 1.442161226 | 0.042662971 | 0.230322101 | Up |
| XLOC_l2_014057 | 1.403858605 | 0.042688989 | 0.230356292 | Up |
| RASAL2 | -1.222271912 | 0.042729034 | 0.2304036 | Down |
| XLOC_l2_004342 | 1.271773492 | 0.042748924 | 0.2304036 | Up |
| BEX5 | -1.223272981 | 0.042808723 | 0.2304036 | Down |
| SIDT1 | -1.189961259 | 0.042825038 | 0.2304036 | Down |
| KRTAP4-11 | 1.191379751 | 0.042841305 | 0.2304036 | Up |
| OR56B4 | 1.487768123 | 0.042897568 | 0.230433071 | Up |
| NAPEPLD | -1.527175692 | 0.042932597 | 0.230433071 | Down |
| CDR1 | -1.545884441 | 0.042937816 | 0.230433071 | Down |
| NPW | 1.142836421 | 0.043082323 | 0.230433071 | Up |
| XLOC_006234 | 1.259428324 | 0.043099173 | 0.230433071 | Up |
| CTTNBP2NL | -1.069276549 | 0.043153106 | 0.230433071 | Down |
| CTCF | -1.011517093 | 0.043178297 | 0.230433071 | Down |
| SCARNA13 | -1.106391008 | 0.043197552 | 0.230433071 | Down |
| XLOC_014287 | 1.402505011 | 0.043214289 | 0.230433071 | Up |
| PAR4 | 1.175686519 | 0.043252811 | 0.230433071 | Up |
| XLOC_014372 | 1.029719171 | 0.043331723 | 0.230433071 | Up |
| PLEKHH2 | -1.130200363 | 0.043363527 | 0.230433071 | Down |
| XLOC_006223 | 1.050266532 | 0.043441172 | 0.230433071 | Up |
| CCDC152 | -1.230302545 | 0.043461975 | 0.230433071 | Down |
| SNAPC4 | -1.035370599 | 0.043463115 | 0.230433071 | Down |
| SDR9C7 | 1.257025912 | 0.043510433 | 0.230433071 | Up |
| SPIN1 | -1.03766419 | 0.043526015 | 0.230433071 | Down |
| GPR26 | 2.138647831 | 0.043555552 | 0.230433071 | Up |
| WFDC9 | 1.288555285 | 0.043566368 | 0.230433071 | Up |
| XLOC_009299 | 1.332985106 | 0.043570164 | 0.230433071 | Up |
| XLOC_l2_011102 | 1.730280724 | 0.043572596 | 0.230433071 | Up |
| GPRASP2 | -1.444925466 | 0.043649534 | 0.230468857 | Down |
| IL17D | -1.353741032 | 0.043669506 | 0.230468857 | Down |
| HYI | -1.058506221 | 0.043684116 | 0.230468857 | Down |
| TAAR8 | 1.046177241 | 0.043809096 | 0.23059455 | Up |
| NDRG1 | -1.197452204 | 0.04391792 | 0.230674098 | Down |
| GOLGA6L6 | 1.418462478 | 0.04392435 | 0.230674098 | Up |
| XLOC_l2_003133 | 1.145367973 | 0.043928378 | 0.230674098 | Up |
| XLOC_000490 | 1.165250301 | 0.043989328 | 0.230694444 | Up |
| WSB1 | -1.012823135 | 0.044109865 | 0.230885704 | Down |
| GPM6B | -1.589449404 | 0.044158076 | 0.230885704 | Down |
| XLOC_010064 | 1.082271208 | 0.04419304 | 0.230885704 | Up |
| ADAMTS8 | -1.547298825 | 0.044224639 | 0.230885704 | Down |
| CD247 | -1.003193547 | 0.044298965 | 0.230885704 | Down |
| SMPDL3A | -1.061922451 | 0.044379303 | 0.230978645 | Down |
| S1PR1 | -1.201702013 | 0.044443604 | 0.231025407 | Down |
| XLOC_l2_013813 | 1.14882305 | 0.044531399 | 0.231228731 | Up |
| XLOC_l2_007541 | 1.270409475 | 0.044576242 | 0.231295294 | Up |
| KIAA1456 | -1.174378517 | 0.044704002 | 0.231698381 | Down |
| VASN | -1.279218978 | 0.044711426 | 0.231698381 | Down |
| INTS8 | -1.01299348 | 0.044714053 | 0.231698381 | Down |
| KCNA4 | 1.439579954 | 0.044841844 | 0.232058453 | Up |
| EGOT | -1.42778505 | 0.044842556 | 0.232058453 | Down |
| XLOC_005039 | 1.018982929 | 0.044853553 | 0.232058453 | Up |
| XLOC_010373 | 1.176807723 | 0.044859868 | 0.232058453 | Up |
| POU2AF1 | -1.57163259 | 0.045011771 | 0.232262027 | Down |
| PCSK7 | -1.011633909 | 0.045062608 | 0.232277572 | Down |
| MOGAT1 | 1.12724611 | 0.045090009 | 0.232277572 | Up |
| CYP4A11 | 1.209266782 | 0.045114151 | 0.232277572 | Up |
| SCG5 | -1.242144248 | 0.045137587 | 0.232277572 | Down |
| FGFR1 | -1.105387656 | 0.045145264 | 0.232277572 | Down |
| XLOC_007150 | 1.071197906 | 0.045162103 | 0.232277572 | Up |
| SNORD28 | -1.100306777 | 0.045238103 | 0.232277572 | Down |
| XLOC_007100 | 1.62598345 | 0.045244256 | 0.232277572 | Up |
| RRAGD | -1.129215595 | 0.045312664 | 0.232277572 | Down |
| THBS2 | -1.375210123 | 0.045315671 | 0.232277572 | Down |
| XLOC_003331 | 1.059464451 | 0.045365198 | 0.232277572 | Up |
| XLOC_012358 | 1.404695127 | 0.045367895 | 0.232277572 | Up |
| DLG4 | -1.498944542 | 0.04538422 | 0.232284231 | Down |
| TCL6 | 1.389763843 | 0.045385915 | 0.232284231 | Up |
| XLOC_001666 | 1.340725602 | 0.045537938 | 0.232611076 | Up |
| OR51A2 | 1.899928074 | 0.045617834 | 0.232667699 | Up |
| TXNL4B | -1.095209313 | 0.045635412 | 0.232667699 | Down |
| DUX4L9 | 1.151803212 | 0.045660798 | 0.232667699 | Up |
| C1QTNF9B | 1.128908951 | 0.04569365 | 0.232787028 | Up |
| XLOC_010253 | 1.135044665 | 0.04577787 | 0.233009534 | Up |
| ZNF26 | -1.034804357 | 0.04581907 | 0.233097156 | Down |
| XLOC_010069 | 1.036300424 | 0.045851867 | 0.233136539 | Up |
| BTF3P11 | -1.023069171 | 0.045884964 | 0.233186017 | Down |
| HLA-DPB2 | -1.162345927 | 0.045911094 | 0.233277775 | Down |
| CREB1 | -1.074927572 | 0.045973272 | 0.233319501 | Down |
| XLOC_003131 | 1.459924255 | 0.046011011 | 0.233319501 | Up |
| OR5AP2 | 1.272033674 | 0.046076543 | 0.23334475 | Up |
| OR2L1P | 1.743218818 | 0.046169617 | 0.233511056 | Up |
| XLOC_002906 | 1.209717217 | 0.046240547 | 0.233636871 | Up |
| XLOC_012880 | 1.35529543 | 0.046257671 | 0.233649522 | Up |
| TRIM77P | 1.46096182 | 0.046395847 | 0.233665641 | Up |
| UNC93A | 1.165212512 | 0.046397476 | 0.233665641 | Up |
| AASS | -1.19847898 | 0.046580711 | 0.233757293 | Down |
| OR4K17 | 1.081724778 | 0.046638892 | 0.233757293 | Up |
| XLOC_007202 | 1.091116761 | 0.046650133 | 0.233757293 | Up |
| XLOC_005178 | 1.281878577 | 0.046705328 | 0.233757293 | Up |
| PAPPA2 | 1.493532112 | 0.046785024 | 0.233757293 | Up |
| DDB1 | 1.306490397 | 0.046846329 | 0.233757293 | Up |
| PTBP1 | -1.098055129 | 0.046877324 | 0.233757293 | Down |
| PRICKLE2 | -1.126615793 | 0.046916145 | 0.233757293 | Down |
| XLOC_l2_010863 | 1.11411935 | 0.046928055 | 0.233757293 | Up |
| LINC00160 | 1.046093427 | 0.047035305 | 0.233849076 | Up |
| XLOC_012823 | 1.186251388 | 0.047074024 | 0.233889145 | Up |
| RPPH1 | -1.240524079 | 0.047148959 | 0.234011437 | Down |
| XLOC_009983 | 1.026236155 | 0.047186951 | 0.234049963 | Up |
| XLOC_003852 | 1.489470439 | 0.047193803 | 0.234049963 | Up |
| XLOC_012166 | 1.262361859 | 0.047215053 | 0.234068781 | Up |
| XLOC_008351 | 1.34371211 | 0.047364682 | 0.234379071 | Up |
| FBXO10 | -1.066688604 | 0.047368741 | 0.234379071 | Down |
| KIAA1210 | -2.45625382 | 0.047830912 | 0.235481616 | Down |
| KRTAP25-1 | 1.043839655 | 0.047852707 | 0.235481616 | Up |
| DDX11L2 | 1.801235152 | 0.047876413 | 0.235505928 | Up |
| ANO6 | 1.061944686 | 0.0479351 | 0.235513226 | Up |
| MGC4473 | 1.170817145 | 0.048117409 | 0.235952327 | Up |
| HLA-DRB6 | 1.033683589 | 0.048144369 | 0.235952327 | Up |
| EPB41 | -1.519219807 | 0.04822813 | 0.236141985 | Down |
| XLOC_l2_001947 | 1.246415532 | 0.048263867 | 0.236184228 | Up |
| XLOC_001500 | 1.078749189 | 0.048303069 | 0.236184228 | Up |
| XLOC_000226 | 1.309642401 | 0.048350232 | 0.236308421 | Up |
| XLOC_013754 | 1.054942173 | 0.048426044 | 0.236381501 | Up |
| PTGDS | -1.685564689 | 0.048501222 | 0.236423138 | Down |
| CRLS1 | -1.059231229 | 0.04852219 | 0.236423138 | Down |
| GALNT8 | 1.624312691 | 0.048533738 | 0.236423138 | Up |
| LRRN4CL | -1.82064572 | 0.048758588 | 0.236594617 | Down |
| PRKACG | 1.412157694 | 0.048799159 | 0.236606216 | Up |
| ADAMTS9 | -1.162188638 | 0.04881832 | 0.236645989 | Down |
| FAM81A | 1.120805659 | 0.048850529 | 0.236656931 | Up |
| PDGFC | -1.207525429 | 0.048883507 | 0.236665956 | Down |
| LTBR | -1.10942195 | 0.048911936 | 0.236680609 | Down |
| XLOC_012694 | 1.320480817 | 0.04891508 | 0.236680609 | Up |
| FOLR2 | -1.164850719 | 0.048982348 | 0.236734911 | Down |
| MIS12 | -1.112679417 | 0.049001147 | 0.236734911 | Down |
| XLOC_005827 | 1.309386 | 0.049004398 | 0.236734911 | Up |
| XLOC_003915 | 1.131199329 | 0.049015662 | 0.236734911 | Up |
| XLOC_011608 | 1.043994763 | 0.049025128 | 0.236734911 | Up |
| FAM84B | -1.028861235 | 0.04906891 | 0.236827577 | Down |
| XLOC_014113 | 1.349034978 | 0.049162855 | 0.236973516 | Up |
| KIAA0195 | -1.090740373 | 0.049187766 | 0.237005296 | Down |
| HOXD10 | -1.057713361 | 0.049365379 | 0.237504607 | Down |
| RPL39L | -1.09790578 | 0.049427658 | 0.237621616 | Down |
| TBX18 | -1.222001972 | 0.049439048 | 0.237621616 | Down |
| OR7E5P | 1.339919591 | 0.049498279 | 0.237737396 | Up |
| CYSLTR2 | 1.079883957 | 0.049593012 | 0.237786139 | Up |
| XLOC_010252 | 1.187210824 | 0.049700956 | 0.23789104 | Up |
| OR2Y1 | 1.001066691 | 0.049909728 | 0.238101381 | Up |
