# Supplementary figures and images for: CCL7 and olfactory transduction pathway activation play an important role in the formation of CaOx and CaP kidney stones
Source: Front Genet. 2024 Jan 3;14:1267545. doi: 10.3389/fgene.2023.1267545 (PMC10791818; doi:10.3389/fgene.2023.1267545)

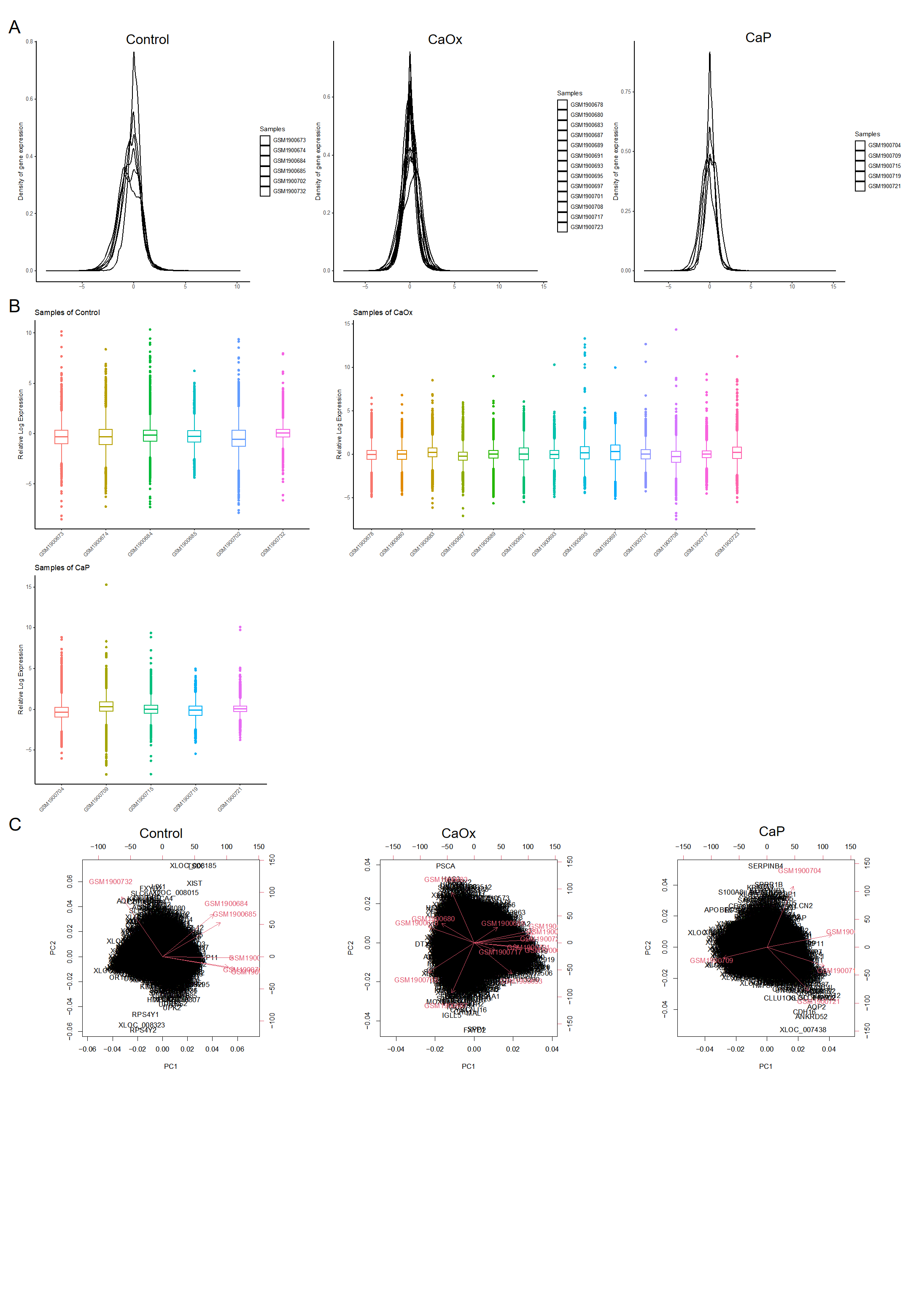

Supplement: Supplementary file 2 [file Image1.TIF]
