# Supplementary material for: CCL7 and olfactory transduction pathway activation play an important role in the formation of CaOx and CaP kidney stones
Source: Front Genet. 2024 Jan 3;14:1267545. doi: 10.3389/fgene.2023.1267545 (PMC10791818; doi:10.3389/fgene.2023.1267545)
Supplement: Supplementary file 3 [file Table2.docx]

Table 2 DEGs of CaP *vs* Control

| Gene.symbol | logFC | P.Value | adj.P.Val | Disease |
| --- | --- | --- | --- | --- |
| XLOC_011768 | 2.034381486 | 5.48635E-05 | 0.712632227 | Up |
| XLOC_002906 | 1.9174796 | 5.7245E-05 | 0.712632227 | Up |
| XLOC_001703 | 1.709364322 | 7.39987E-05 | 0.712632227 | Up |
| XLOC_013862 | 2.223785569 | 0.000231217 | 0.990553456 | Up |
| XLOC_000318 | 1.514952727 | 0.000275995 | 0.990553456 | Up |
| XLOC_000832 | 1.694982462 | 0.000276878 | 0.990553456 | Up |
| MMP10 | 2.319806996 | 0.000287916 | 0.990553456 | Up |
| GRM8 | 1.55192958 | 0.000314951 | 0.990553456 | Up |
| PTCHD2 | 1.56180506 | 0.000332085 | 0.990553456 | Up |
| XLOC_001823 | 1.600516563 | 0.000420655 | 0.990553456 | Up |
| XLOC_l2_008124 | 1.738012495 | 0.000453008 | 0.990553456 | Up |
| KCNQ1OT1 | -1.441362008 | 0.000531517 | 0.990553456 | Down |
| XLOC_002830 | 1.331240313 | 0.000537309 | 0.990553456 | Up |
| ACY3 | 1.434868519 | 0.000538797 | 0.990553456 | Up |
| XLOC_l2_002290 | 1.193076814 | 0.000566093 | 0.990553456 | Up |
| IKBIP | 1.748285506 | 0.000620655 | 0.990553456 | Up |
| OR52E2 | 1.397079118 | 0.000726137 | 0.990553456 | Up |
| WDR76 | 1.412480108 | 0.000896273 | 0.990553456 | Up |
| XLOC_003166 | 1.421582999 | 0.000924507 | 0.990553456 | Up |
| CD80 | 1.994895442 | 0.001001301 | 0.990553456 | Up |
| DUSP7 | 1.439565685 | 0.001044098 | 0.990553456 | Up |
| DEC1 | 1.925926649 | 0.0011079 | 0.990553456 | Up |
| PLIN2 | -2.194368283 | 0.001227123 | 0.990553456 | Down |
| XLOC_002941 | 1.406115258 | 0.001512761 | 0.990553456 | Up |
| TMEM63C | 1.403479416 | 0.001617651 | 0.990553456 | Up |
| XLOC_008624 | 1.250182457 | 0.001633581 | 0.990553456 | Up |
| MKI67 | 2.160384226 | 0.001636193 | 0.990553456 | Up |
| BARX2 | 1.669600022 | 0.001644947 | 0.990553456 | Up |
| PPAPDC3 | 1.090069486 | 0.001646189 | 0.990553456 | Up |
| XLOC_011252 | 1.391898129 | 0.001747593 | 0.990553456 | Up |
| BUB1 | 1.647281068 | 0.001755718 | 0.990553456 | Up |
| OR5B2 | 1.172886717 | 0.001782531 | 0.990553456 | Up |
| XLOC_002577 | -1.774234362 | 0.001804028 | 0.990553456 | Down |
| GAS2 | -1.236040471 | 0.001854509 | 0.990553456 | Down |
| UCP1 | 1.661736393 | 0.001988128 | 0.990553456 | Up |
| NCR2 | 1.436057274 | 0.002021025 | 0.990553456 | Up |
| AQP7P3 | -1.401687433 | 0.002163038 | 0.990553456 | Down |
| CDC42SE2 | 1.882660961 | 0.002228588 | 0.990553456 | Up |
| XLOC_007495 | -1.397682492 | 0.002340905 | 0.990553456 | Down |
| OR8K3 | 1.745770396 | 0.002341909 | 0.990553456 | Up |
| SPRR2C | 1.977222839 | 0.002448909 | 0.990553456 | Up |
| WDR65 | 1.323594368 | 0.002449721 | 0.990553456 | Up |
| XLOC_002366 | 1.59755563 | 0.002582228 | 0.990553456 | Up |
| KCNH8 | 1.438204822 | 0.002632789 | 0.990553456 | Up |
| MUCL1 | 1.829235674 | 0.002650638 | 0.990553456 | Up |
| FAM83A | 1.441045197 | 0.002714697 | 0.990553456 | Up |
| XLOC_002887 | 1.588511651 | 0.002796476 | 0.990553456 | Up |
| CDKN3 | 1.335107847 | 0.002799864 | 0.990553456 | Up |
| MGC24103 | -1.070963426 | 0.002818157 | 0.990553456 | Down |
| XLOC_002524 | 1.276752645 | 0.002844445 | 0.990553456 | Up |
| XLOC_001525 | 1.099899653 | 0.002892736 | 0.990553456 | Up |
| XLOC_007496 | 1.549516953 | 0.002918443 | 0.990553456 | Up |
| SPATA5L1 | 1.376821854 | 0.002967799 | 0.990553456 | Up |
| SPIRE1 | -1.418141671 | 0.00297851 | 0.990553456 | Down |
| GATAD2B | -1.649832828 | 0.003034811 | 0.990553456 | Down |
| XLOC_005254 | 1.154567807 | 0.003059152 | 0.990553456 | Up |
| ZNF536 | -1.700969786 | 0.003135671 | 0.990553456 | Down |
| TMEM207 | 1.569417461 | 0.003161516 | 0.990553456 | Up |
| MED28 | -1.060222429 | 0.003246564 | 0.990553456 | Down |
| PTPRR | -2.044730373 | 0.003405965 | 0.990553456 | Down |
| MIR137HG | 1.55185319 | 0.003521795 | 0.990553456 | Up |
| XLOC_001607 | 1.525728232 | 0.003716441 | 0.990553456 | Up |
| NGEF | 1.267366767 | 0.003717379 | 0.990553456 | Up |
| XLOC_011951 | 1.859023332 | 0.003758428 | 0.990553456 | Up |
| RASGRP3 | 1.268275951 | 0.003797044 | 0.990553456 | Up |
| XLOC_010840 | 1.525475409 | 0.003802265 | 0.990553456 | Up |
| PHF8 | -1.211394597 | 0.003813673 | 0.990553456 | Down |
| XLOC_012013 | 1.088055279 | 0.003890975 | 0.990553456 | Up |
| XLOC_l2_005759 | 1.425021858 | 0.003904314 | 0.990553456 | Up |
| CBR1 | 1.257859763 | 0.003941863 | 0.990553456 | Up |
| XLOC_l2_011120 | 1.221120285 | 0.004040346 | 0.990553456 | Up |
| TNFSF11 | 1.438424377 | 0.004151231 | 0.990553456 | Up |
| RSPO1 | 1.339452503 | 0.004183188 | 0.990553456 | Up |
| XLOC_l2_006737 | -1.256368773 | 0.00419203 | 0.990553456 | Down |
| XLOC_003926 | 1.315696502 | 0.004253616 | 0.990553456 | Up |
| XLOC_001877 | 1.431889384 | 0.004307347 | 0.990553456 | Up |
| HOXB1 | 1.055483993 | 0.004316466 | 0.990553456 | Up |
| KLHDC8A | 1.466463642 | 0.004377599 | 0.990553456 | Up |
| XLOC_006383 | 1.17865329 | 0.004379459 | 0.990553456 | Up |
| XLOC_012014 | 1.309899363 | 0.004379593 | 0.990553456 | Up |
| HES2 | 1.493662458 | 0.004484218 | 0.990553456 | Up |
| XLOC_007488 | 1.395840857 | 0.0044948 | 0.990553456 | Up |
| XLOC_002997 | -1.273822144 | 0.004560089 | 0.990553456 | Down |
| FRY-AS1 | -1.412107952 | 0.004682628 | 0.990553456 | Down |
| GK2 | 1.552719752 | 0.004758834 | 0.990553456 | Up |
| AMELY | 1.454110284 | 0.004864894 | 0.990553456 | Up |
| PLSCR5 | 1.561080872 | 0.004897413 | 0.990553456 | Up |
| C9orf140 | 1.297067076 | 0.004938585 | 0.990553456 | Up |
| XLOC_004827 | 1.181638643 | 0.004954458 | 0.990553456 | Up |
| OR9A2 | 1.349708876 | 0.005019475 | 0.990553456 | Up |
| OR11H12 | 2.738526934 | 0.00505908 | 0.990553456 | Up |
| PAX5 | 1.421171432 | 0.005091173 | 0.990553456 | Up |
| XLOC_003906 | 1.26735075 | 0.005294307 | 0.990553456 | Up |
| XLOC_014038 | 1.784411952 | 0.005364278 | 0.990553456 | Up |
| XLOC_011238 | 1.230701213 | 0.005371781 | 0.990553456 | Up |
| XLOC_003811 | 1.513631349 | 0.005406049 | 0.990553456 | Up |
| HSFY2 | 2.970301479 | 0.005483375 | 0.990553456 | Up |
| XLOC_001907 | 1.269709074 | 0.005491442 | 0.990553456 | Up |
| XLOC_l2_015143 | 1.542735199 | 0.005500156 | 0.990553456 | Up |
| XLOC_012626 | 1.69126282 | 0.005505349 | 0.990553456 | Up |
| CASQ2 | -1.150851614 | 0.005594852 | 0.990553456 | Down |
| TRIM67 | 1.319796874 | 0.005613245 | 0.990553456 | Up |
| XLOC_011712 | 1.196960517 | 0.005653634 | 0.990553456 | Up |
| ITPK1-AS1 | -1.100705993 | 0.005777648 | 0.990553456 | Down |
| XLOC_004260 | 1.132237167 | 0.005871083 | 0.990553456 | Up |
| CYP4F35P | -1.622470967 | 0.005974403 | 0.990553456 | Down |
| FLJ23865 | 1.462113229 | 0.00604344 | 0.990553456 | Up |
| ASTN1 | 1.326234145 | 0.006055866 | 0.990553456 | Up |
| XLOC_006664 | 1.068700247 | 0.006106564 | 0.990553456 | Up |
| NSUN6 | -1.454528011 | 0.006180589 | 0.990553456 | Down |
| ELAVL4 | 1.263560886 | 0.006228292 | 0.990553456 | Up |
| HRH4 | -2.114141523 | 0.006253293 | 0.990553456 | Down |
| SDHAP1 | -1.105768655 | 0.006409848 | 0.990553456 | Down |
| XLOC_006929 | 1.393969707 | 0.006515736 | 0.990553456 | Up |
| LMTK2 | -1.426088276 | 0.006523168 | 0.990553456 | Down |
| NEBL | -1.202149658 | 0.006550611 | 0.990553456 | Down |
| XLOC_005918 | 1.691398103 | 0.00657869 | 0.990553456 | Up |
| XLOC_010674 | 1.168243058 | 0.006702055 | 0.990553456 | Up |
| ZNF385D | 1.449285586 | 0.006728173 | 0.990553456 | Up |
| IL11 | 2.585630296 | 0.006743574 | 0.990553456 | Up |
| SPATA16 | 1.424623312 | 0.00697798 | 0.990553456 | Up |
| XLOC_012849 | 1.350278042 | 0.006994389 | 0.990553456 | Up |
| XLOC_013579 | -1.1663295 | 0.007020522 | 0.990553456 | Down |
| SAGE1 | 1.091180728 | 0.00702803 | 0.990553456 | Up |
| XLOC_005954 | 1.405037575 | 0.007040332 | 0.990553456 | Up |
| CEP78 | -1.003548824 | 0.00704532 | 0.990553456 | Down |
| STAB2 | 1.282905687 | 0.007088279 | 0.990553456 | Up |
| XLOC_012664 | 1.117506168 | 0.007155265 | 0.990553456 | Up |
| HOTAIRM1 | -1.29197918 | 0.007176504 | 0.990553456 | Down |
| XLOC_008222 | 1.449566062 | 0.007189996 | 0.990553456 | Up |
| ARMC2 | 1.012497767 | 0.00726697 | 0.990553456 | Up |
| ANKS4B | 1.620653818 | 0.007373459 | 0.990553456 | Up |
| CXCR2 | -1.966838048 | 0.007592853 | 0.990553456 | Down |
| CERS4 | -1.082117312 | 0.007650555 | 0.990553456 | Down |
| THSD4 | -1.216048106 | 0.007683988 | 0.990553456 | Down |
| TTC32 | -1.170198226 | 0.007774823 | 0.990553456 | Down |
| ATG14 | -1.082167989 | 0.007803532 | 0.990553456 | Down |
| CCL7 | 1.436606948 | 0.00787391 | 0.990553456 | Up |
| XLOC_003970 | 1.102491689 | 0.008016422 | 0.990553456 | Up |
| RBM34 | -1.336294813 | 0.008041709 | 0.990553456 | Down |
| FLJ40194 | -1.318300208 | 0.008045824 | 0.990553456 | Down |
| ORC1 | 1.231034516 | 0.008074301 | 0.990553456 | Up |
| CMYA5 | -1.630736216 | 0.008075778 | 0.990553456 | Down |
| C20orf194 | -1.127012167 | 0.008240225 | 0.990553456 | Down |
| IL36G | 1.345203958 | 0.008301047 | 0.990553456 | Up |
| XLOC_002122 | 1.122499242 | 0.008410099 | 0.990553456 | Up |
| PKMYT1 | 1.485845526 | 0.008412079 | 0.990553456 | Up |
| VSIG1 | -1.453210555 | 0.008508121 | 0.990553456 | Down |
| MYBPC2 | 1.223272617 | 0.008541744 | 0.990553456 | Up |
| XLOC_007186 | 1.175878642 | 0.008678843 | 0.990553456 | Up |
| AQR | -1.030323585 | 0.008680972 | 0.990553456 | Down |
| LCN12 | 1.305593635 | 0.008718695 | 0.990553456 | Up |
| XLOC_007389 | 1.071823497 | 0.008826014 | 0.990553456 | Up |
| XLOC_012876 | -1.381057268 | 0.008841887 | 0.990553456 | Down |
| XLOC_013467 | 1.484741875 | 0.008851901 | 0.990553456 | Up |
| SNORD72 | -1.23650532 | 0.008864756 | 0.990553456 | Down |
| GRHL2 | -1.734387814 | 0.008877229 | 0.990553456 | Down |
| MRGPRX2 | 1.374670833 | 0.008919377 | 0.990553456 | Up |
| CA5A | 1.165594709 | 0.008958576 | 0.990553456 | Up |
| XLOC_014115 | 1.171892436 | 0.008970068 | 0.990553456 | Up |
| C1orf49 | 1.216977912 | 0.008973292 | 0.990553456 | Up |
| DEFA11P | 1.186534079 | 0.008993288 | 0.990553456 | Up |
| XLOC_007883 | 1.02907505 | 0.009026603 | 0.990553456 | Up |
| XLOC_003385 | 1.265117592 | 0.009144195 | 0.990553456 | Up |
| ABCC6P1 | 1.159115549 | 0.009200927 | 0.990553456 | Up |
| XLOC_003838 | 1.221009447 | 0.009479995 | 0.990553456 | Up |
| XLOC_005180 | 1.142112724 | 0.009566684 | 0.990553456 | Up |
| KIAA1751 | 1.041167554 | 0.009639448 | 0.990553456 | Up |
| XLOC_005889 | -1.185556559 | 0.009656025 | 0.990553456 | Down |
| LPHN1 | 2.094048497 | 0.009681612 | 0.990553456 | Up |
| CDK1 | 1.24371239 | 0.009688334 | 0.990553456 | Up |
| GNG13 | 2.174714216 | 0.009706569 | 0.990553456 | Up |
| XLOC_006419 | 1.171492278 | 0.009709419 | 0.990553456 | Up |
| XLOC_014154 | 1.074400809 | 0.00977089 | 0.990553456 | Up |
| XLOC_001357 | 1.245380544 | 0.009777533 | 0.990553456 | Up |
| CAGE1 | 1.478945638 | 0.010014905 | 0.990553456 | Up |
| SNTG2 | 1.212419002 | 0.010029248 | 0.990553456 | Up |
| XLOC_012009 | -1.249185641 | 0.010054211 | 0.990553456 | Down |
| XLOC_001365 | 1.002607031 | 0.010061822 | 0.990553456 | Up |
| XLOC_007155 | 1.533292589 | 0.010066734 | 0.990553456 | Up |
| XLOC_006652 | 2.061359694 | 0.010149094 | 0.990553456 | Up |
| ARL10 | 1.09868374 | 0.010173784 | 0.990553456 | Up |
| XLOC_010427 | 1.232700528 | 0.010200889 | 0.990553456 | Up |
| XLOC_011023 | 2.196160229 | 0.010269993 | 0.990553456 | Up |
| OR4A16 | 1.123713798 | 0.010365593 | 0.990553456 | Up |
| XLOC_004914 | 1.305687594 | 0.010412953 | 0.990553456 | Up |
| FAM9A | 2.026583616 | 0.010417362 | 0.990553456 | Up |
| OR10K1 | 1.271485421 | 0.010454871 | 0.990553456 | Up |
| XLOC_011706 | 1.723333482 | 0.010650646 | 0.990553456 | Up |
| EFCAB6 | 1.187873103 | 0.010747124 | 0.990553456 | Up |
| XLOC_005210 | 1.264922944 | 0.010782641 | 0.990553456 | Up |
| NRXN3 | -1.009344273 | 0.010783001 | 0.990553456 | Down |
| PIH1D2 | 1.840275259 | 0.010849413 | 0.990553456 | Up |
| XLOC_011284 | -1.471261343 | 0.010906572 | 0.990553456 | Down |
| XLOC_000877 | 1.211223515 | 0.010960882 | 0.990553456 | Up |
| XLOC_010948 | 1.420790226 | 0.011011983 | 0.990553456 | Up |
| TMEM72 | 1.584508198 | 0.011139713 | 0.990553456 | Up |
| L3MBTL2 | 1.551815457 | 0.011151688 | 0.990553456 | Up |
| XLOC_007531 | 1.560596145 | 0.011194473 | 0.990553456 | Up |
| FAM18B2-CDRT4 | -1.19852085 | 0.0112416 | 0.990553456 | Down |
| KIF5C | -2.001047865 | 0.011256339 | 0.990553456 | Down |
| XLOC_004090 | 1.233549221 | 0.011274696 | 0.990553456 | Up |
| LDLRAD3 | 1.164301309 | 0.011310786 | 0.990553456 | Up |
| XLOC_014409 | -1.08723893 | 0.011355129 | 0.990553456 | Down |
| C7orf41 | -1.097886391 | 0.011359663 | 0.990553456 | Down |
| XLOC_010776 | 1.120769251 | 0.011388187 | 0.990553456 | Up |
| XLOC_008186 | -1.648602361 | 0.011404543 | 0.990553456 | Down |
| OR51B4 | 1.127824142 | 0.011507564 | 0.990553456 | Up |
| XLOC_002882 | 1.219604254 | 0.011626146 | 0.990553456 | Up |
| DAZL | 1.612695678 | 0.01163718 | 0.990553456 | Up |
| POFUT1 | 1.388944309 | 0.011642836 | 0.990553456 | Up |
| XLOC_001286 | 1.128637877 | 0.011754236 | 0.990553456 | Up |
| XLOC_004877 | -1.097564227 | 0.011779339 | 0.990553456 | Down |
| XLOC_l2_000915 | 1.004717002 | 0.011781934 | 0.990553456 | Up |
| ACVR2B | -1.34845041 | 0.011782661 | 0.990553456 | Down |
| KIAA1324 | 1.411439254 | 0.011921102 | 0.990553456 | Up |
| GRPR | 1.053748619 | 0.012076823 | 0.990553456 | Up |
| AIM2 | 1.542275009 | 0.012099703 | 0.990553456 | Up |
| OR11L1 | -1.009322041 | 0.012131977 | 0.990553456 | Down |
| PIP5K1P1 | 1.185105852 | 0.012299296 | 0.990553456 | Up |
| XLOC_013812 | 1.32356954 | 0.012345275 | 0.990553456 | Up |
| KLK1 | 1.910726447 | 0.012351875 | 0.990553456 | Up |
| IDI2 | 1.26603296 | 0.012369832 | 0.990553456 | Up |
| XLOC_009810 | 1.258463158 | 0.012374767 | 0.990553456 | Up |
| RNASEL | -1.253240621 | 0.012384562 | 0.990553456 | Down |
| XLOC_012616 | -1.39165059 | 0.012394498 | 0.990553456 | Down |
| ACSL6 | 1.409701333 | 0.012476524 | 0.990553456 | Up |
| ESCO1 | -1.328486894 | 0.012508618 | 0.990553456 | Down |
| TRIM33 | -1.06439726 | 0.012535838 | 0.990553456 | Down |
| XLOC_004872 | 1.315759239 | 0.0125402 | 0.990553456 | Up |
| XLOC_007268 | 1.335470079 | 0.012542845 | 0.990553456 | Up |
| XLOC_l2_013056 | 1.061300542 | 0.012672531 | 0.990553456 | Up |
| XLOC_l2_014959 | -1.462053121 | 0.012827185 | 0.990553456 | Down |
| XLOC_009458 | -1.25961337 | 0.012884673 | 0.990553456 | Down |
| ANKRD34A | 1.330297154 | 0.012890285 | 0.990553456 | Up |
| RBMY3AP | 1.786589725 | 0.012920184 | 0.990553456 | Up |
| XLOC_002571 | -1.152654485 | 0.012924654 | 0.990553456 | Down |
| XLOC_006316 | 1.522258256 | 0.012964175 | 0.990553456 | Up |
| XLOC_005910 | 1.925217372 | 0.012984071 | 0.990553456 | Up |
| XLOC_010767 | -1.912018847 | 0.013072035 | 0.990553456 | Down |
| XLOC_011807 | 1.04574098 | 0.013161122 | 0.990553456 | Up |
| XLOC_005160 | 1.081463212 | 0.013183544 | 0.990553456 | Up |
| XLOC_010248 | 1.23929702 | 0.013201023 | 0.990553456 | Up |
| C10orf108 | -1.47644917 | 0.013221577 | 0.990553456 | Down |
| UBQLN3 | 1.187639459 | 0.013228969 | 0.990553456 | Up |
| XLOC_006520 | 2.005264709 | 0.013289493 | 0.990553456 | Up |
| TTR | -1.559753905 | 0.013296676 | 0.990553456 | Down |
| C9 | 1.199913506 | 0.013341509 | 0.990553456 | Up |
| CRISP1 | 1.131252109 | 0.013390274 | 0.990553456 | Up |
| RGSL1 | 1.090678286 | 0.013434709 | 0.990553456 | Up |
| PRAMEF5 | -1.020573764 | 0.013548693 | 0.990553456 | Down |
| XLOC_007746 | 1.231238378 | 0.013554018 | 0.990553456 | Up |
| EPHA7 | 1.205882442 | 0.013626472 | 0.990553456 | Up |
| XLOC_013285 | 1.507044213 | 0.013729147 | 0.990553456 | Up |
| XLOC_011468 | 1.865366909 | 0.013788549 | 0.990553456 | Up |
| C3orf30 | 1.045501359 | 0.013798163 | 0.990553456 | Up |
| FATE1 | 1.101790596 | 0.013847222 | 0.990553456 | Up |
| TYW5 | 1.287181859 | 0.013851184 | 0.990553456 | Up |
| OR1L3 | 1.727228912 | 0.013852029 | 0.990553456 | Up |
| TESC | -1.540775703 | 0.013899988 | 0.990553456 | Down |
| XLOC_010357 | 1.076397229 | 0.013943488 | 0.990553456 | Up |
| XLOC_003987 | -1.02661076 | 0.013959369 | 0.990553456 | Down |
| XLOC_004350 | 1.437064547 | 0.013960296 | 0.990553456 | Up |
| TSHR | 1.098456047 | 0.014027747 | 0.990553456 | Up |
| XLOC_l2_007907 | 1.028744031 | 0.0142091 | 0.990553456 | Up |
| Q68PJ0 | 1.237912365 | 0.014551549 | 0.990553456 | Up |
| XLOC_004331 | -1.483756747 | 0.01459319 | 0.990553456 | Down |
| XLOC_012442 | 1.101303086 | 0.014640876 | 0.990553456 | Up |
| FAM170A | 1.187765638 | 0.014652463 | 0.990553456 | Up |
| XLOC_000641 | 1.220236359 | 0.014798607 | 0.990553456 | Up |
| XLOC_008823 | -1.034693605 | 0.014831159 | 0.990553456 | Down |
| PIGZ | -1.284137048 | 0.014835236 | 0.990553456 | Down |
| OR8D2 | 1.111151732 | 0.014843742 | 0.990553456 | Up |
| PODN | 1.37540982 | 0.014866877 | 0.990553456 | Up |
| CELF6 | 1.20683004 | 0.015035779 | 0.990553456 | Up |
| TOR1AIP1 | -1.018027084 | 0.01504708 | 0.990553456 | Down |
| XLOC_l2_006138 | 1.243566832 | 0.015071804 | 0.990553456 | Up |
| KDM4D | 1.40312235 | 0.015136222 | 0.990553456 | Up |
| XLOC_011179 | -1.639601784 | 0.015144686 | 0.990553456 | Down |
| MARC1 | -1.232661514 | 0.015167729 | 0.990553456 | Down |
| XLOC_l2_012932 | 1.036502412 | 0.015191459 | 0.990553456 | Up |
| XLOC_002924 | 1.296365912 | 0.015217076 | 0.990553456 | Up |
| CCDC109B | 1.066317353 | 0.015303743 | 0.990553456 | Up |
| NKX6-1 | 1.095437411 | 0.015355146 | 0.990553456 | Up |
| XLOC_007753 | 1.613487275 | 0.015450125 | 0.990553456 | Up |
| TMEM155 | 1.308526379 | 0.015637121 | 0.990553456 | Up |
| XLOC_002176 | 1.452166199 | 0.015669246 | 0.990553456 | Up |
| XLOC_011818 | 1.252016372 | 0.015708575 | 0.990553456 | Up |
| XLOC_l2_013315 | 1.417028622 | 0.015720948 | 0.990553456 | Up |
| XLOC_006253 | 1.058874861 | 0.015741165 | 0.990553456 | Up |
| MYT1L | 1.15951716 | 0.015761785 | 0.990553456 | Up |
| GRIK3 | -1.366300555 | 0.015800576 | 0.990553456 | Down |
| XLOC_003889 | 1.104288298 | 0.015954169 | 0.990553456 | Up |
| SCARNA22 | 1.078342536 | 0.015978722 | 0.990553456 | Up |
| MALAT1 | -1.298723763 | 0.016014068 | 0.990553456 | Down |
| XLOC_003840 | 1.018719776 | 0.016031908 | 0.990553456 | Up |
| XLOC_011405 | 1.173627398 | 0.016045316 | 0.990553456 | Up |
| NEAT1 | -1.256779531 | 0.016219301 | 0.990553456 | Down |
| KREMEN2 | 1.007755311 | 0.016265753 | 0.990553456 | Up |
| HTR2A | -1.642340818 | 0.0163141 | 0.990553456 | Down |
| COL19A1 | -1.716827682 | 0.016418415 | 0.990553456 | Down |
| LHX5 | 1.036748156 | 0.016455168 | 0.990553456 | Up |
| RNASEH1 | 1.223824216 | 0.016504242 | 0.990553456 | Up |
| XLOC_011136 | 1.043872663 | 0.016510533 | 0.990553456 | Up |
| SLC26A2 | -1.065852529 | 0.016526233 | 0.990553456 | Down |
| KRTAP4-2 | 1.169943315 | 0.016633618 | 0.990553456 | Up |
| IFNG | 1.073110194 | 0.016680806 | 0.990553456 | Up |
| XLOC_l2_001288 | 1.342028416 | 0.01678472 | 0.990553456 | Up |
| WNT9B | 1.254601995 | 0.01678506 | 0.990553456 | Up |
| RIC3 | -1.185564979 | 0.016912111 | 0.990553456 | Down |
| ZNF765 | -1.112348092 | 0.016927653 | 0.990553456 | Down |
| C1orf144 | 1.340418301 | 0.016965861 | 0.990553456 | Up |
| SPEM1 | 1.231376987 | 0.017030609 | 0.990553456 | Up |
| XLOC_012525 | 1.234825157 | 0.017252431 | 0.990553456 | Up |
| AHNAK2 | -1.362751622 | 0.017340904 | 0.990553456 | Down |
| WDFY3 | -1.297180621 | 0.017350846 | 0.990553456 | Down |
| PSG2 | 1.371516466 | 0.017364799 | 0.990553456 | Up |
| PCDHB17 | 1.350786506 | 0.01742343 | 0.990553456 | Up |
| XLOC_003548 | 1.06981268 | 0.017439958 | 0.990553456 | Up |
| XLOC_013274 | -1.419757013 | 0.017527997 | 0.990553456 | Down |
| XLOC_l2_001889 | 1.170781329 | 0.017583097 | 0.990553456 | Up |
| XLOC_006226 | 1.255158136 | 0.017641293 | 0.990553456 | Up |
| PPP2R1A | 1.441458142 | 0.017699682 | 0.990553456 | Up |
| CHN1 | 1.212572772 | 0.017765332 | 0.990553456 | Up |
| XLOC_001787 | 1.243368518 | 0.017869115 | 0.990553456 | Up |
| C2orf62 | 1.10414967 | 0.017981243 | 0.990553456 | Up |
| PATE2 | 1.474506276 | 0.017987987 | 0.990553456 | Up |
| LCK | 1.482365248 | 0.01800961 | 0.990553456 | Up |
| PPP1R2P9 | 1.356985214 | 0.018078306 | 0.990553456 | Up |
| CFL2 | -1.420035295 | 0.018103187 | 0.990553456 | Down |
| XLOC_001769 | 1.222338712 | 0.018140335 | 0.990553456 | Up |
| XLOC_003773 | 1.11470714 | 0.018163137 | 0.990553456 | Up |
| RPA4 | -1.196733149 | 0.018173148 | 0.990553456 | Down |
| CDH8 | 1.160977698 | 0.018218361 | 0.990553456 | Up |
| XLOC_001960 | 1.020349925 | 0.018243356 | 0.990553456 | Up |
| XLOC_011568 | 1.688917168 | 0.018593291 | 0.990553456 | Up |
| PDCD1LG2 | 1.383722755 | 0.018647971 | 0.990553456 | Up |
| XLOC_007681 | 1.420695281 | 0.018723221 | 0.990553456 | Up |
| XLOC_008351 | 2.088725349 | 0.018759122 | 0.990553456 | Up |
| XLOC_012708 | 1.272498606 | 0.018780906 | 0.990553456 | Up |
| TMPRSS6 | 1.243091233 | 0.018792554 | 0.990553456 | Up |
| XLOC_l2_005187 | 1.1802325 | 0.01879758 | 0.990553456 | Up |
| XLOC_l2_001557 | 1.298491674 | 0.018859322 | 0.990553456 | Up |
| MED13 | -1.021617508 | 0.01892798 | 0.990553456 | Down |
| KL | 1.077191894 | 0.01893015 | 0.990553456 | Up |
| LTA | 1.170524595 | 0.018934925 | 0.990553456 | Up |
| KLK13 | 1.029821576 | 0.018994684 | 0.990553456 | Up |
| PRAC | 1.042052549 | 0.019087372 | 0.990553456 | Up |
| ZNF91 | -1.160344015 | 0.019090448 | 0.990553456 | Down |
| KIF15 | 1.21609487 | 0.019202414 | 0.990553456 | Up |
| PPP1R3B | -1.156029171 | 0.019228924 | 0.990553456 | Down |
| XLOC_l2_002611 | 1.272441833 | 0.019468046 | 0.990553456 | Up |
| XLOC_001108 | 1.056002358 | 0.019470686 | 0.990553456 | Up |
| WARS2 | -1.055803957 | 0.019548752 | 0.990553456 | Down |
| XLOC_l2_001037 | 1.258469507 | 0.019551862 | 0.990553456 | Up |
| SPP2 | 1.165726136 | 0.019582886 | 0.990553456 | Up |
| XLOC_000138 | 1.062704063 | 0.019633947 | 0.990553456 | Up |
| XLOC_000849 | 1.442934705 | 0.019712397 | 0.990553456 | Up |
| GP1BB | -1.132354505 | 0.019751054 | 0.990553456 | Down |
| LONRF2 | -1.329905793 | 0.019798593 | 0.990553456 | Down |
| XLOC_l2_001954 | 1.210628109 | 0.019920739 | 0.990553456 | Up |
| CHI3L1 | 2.984879141 | 0.019964705 | 0.990553456 | Up |
| HTR5A | 1.160287565 | 0.020074531 | 0.990553456 | Up |
| MOV10L1 | 1.052899342 | 0.020087391 | 0.990553456 | Up |
| DYNC1LI2 | -1.024330085 | 0.020266724 | 0.990553456 | Down |
| TMTC3 | -1.147357176 | 0.02035754 | 0.990553456 | Down |
| XLOC_007643 | 1.773490348 | 0.020423518 | 0.990553456 | Up |
| RECQL5 | 1.353521687 | 0.020468941 | 0.990553456 | Up |
| XLOC_005306 | 1.201547776 | 0.020488494 | 0.990553456 | Up |
| XLOC_014368 | -1.159643312 | 0.020505254 | 0.990553456 | Down |
| MGC23270 | 1.091493224 | 0.020519318 | 0.990553456 | Up |
| FMO1 | 1.360708454 | 0.020542195 | 0.990553456 | Up |
| XLOC_l2_015661 | 2.252446641 | 0.02065041 | 0.990553456 | Up |
| XLOC_l2_014820 | -1.271420036 | 0.020713313 | 0.990553456 | Down |
| IYD | 1.077912099 | 0.020728722 | 0.990553456 | Up |
| XLOC_009900 | 1.205330666 | 0.020738301 | 0.990553456 | Up |
| TNMD | 1.514990855 | 0.020768933 | 0.990553456 | Up |
| SLC16A10 | 1.002814718 | 0.020800796 | 0.990553456 | Up |
| LEAP2 | -1.453262908 | 0.020818723 | 0.990553456 | Down |
| GPRC6A | 1.090603146 | 0.020906079 | 0.990553456 | Up |
| XLOC_007235 | 1.367122186 | 0.020940983 | 0.990553456 | Up |
| ERCC6L | 1.194050385 | 0.021123771 | 0.990553456 | Up |
| PDXP | 1.04892186 | 0.021136033 | 0.990553456 | Up |
| NEU2 | 1.019345652 | 0.021175327 | 0.990553456 | Up |
| ZFHX3 | -1.160900013 | 0.02119582 | 0.990553456 | Down |
| XLOC_008729 | 1.194239971 | 0.021209945 | 0.990553456 | Up |
| MED13L | -1.156516726 | 0.021218598 | 0.990553456 | Down |
| XLOC_011316 | 1.965858918 | 0.021234778 | 0.990553456 | Up |
| DOC2A | 1.2960854 | 0.021329709 | 0.990553456 | Up |
| XLOC_009689 | 1.091033573 | 0.021392066 | 0.990553456 | Up |
| RCOR2 | -1.160743539 | 0.021528811 | 0.990553456 | Down |
| BMX | 1.411467229 | 0.021562303 | 0.990553456 | Up |
| XLOC_005593 | 1.139457253 | 0.021704082 | 0.990553456 | Up |
| BOLL | 1.158624299 | 0.021718608 | 0.990553456 | Up |
| XLOC_013687 | 1.048820312 | 0.022055084 | 0.990553456 | Up |
| CXCR7 | -1.116797569 | 0.022128449 | 0.990553456 | Down |
| ZNF816-ZNF321P | -1.153282879 | 0.02224082 | 0.990553456 | Down |
| MCOLN3 | 1.44440165 | 0.022250541 | 0.990553456 | Up |
| CXCL9 | 2.109761613 | 0.022262093 | 0.990553456 | Up |
| SNAI3 | 1.591083058 | 0.022266379 | 0.990553456 | Up |
| XLOC_007528 | 1.142772656 | 0.022266701 | 0.990553456 | Up |
| CCDC14 | -1.146115481 | 0.022428998 | 0.990553456 | Down |
| ARSJ | 1.242027201 | 0.022434193 | 0.990553456 | Up |
| GLYCAM1 | -1.544576882 | 0.02247712 | 0.990553456 | Down |
| C7orf55 | -1.320062963 | 0.022502433 | 0.990553456 | Down |
| XLOC_006314 | 1.004824066 | 0.022613553 | 0.990553456 | Up |
| ADAMTS18 | 1.151376479 | 0.022684208 | 0.990553456 | Up |
| XLOC_009250 | 1.726656508 | 0.022685693 | 0.990553456 | Up |
| HCN1 | 1.082124979 | 0.022717855 | 0.990553456 | Up |
| C5orf25 | -1.011821298 | 0.022739407 | 0.990553456 | Down |
| PLOD3 | 1.316093125 | 0.02274749 | 0.990553456 | Up |
| XLOC_003796 | -1.042182595 | 0.022764916 | 0.990553456 | Down |
| PI15 | -2.543660361 | 0.022819197 | 0.990553456 | Down |
| OR4K17 | 1.186816175 | 0.022965401 | 0.990553456 | Up |
| FLJ41130 | 1.134903466 | 0.022994816 | 0.990553456 | Up |
| CLECL1 | 1.281398548 | 0.022997177 | 0.990553456 | Up |
| PCDHA11 | 1.284629966 | 0.023037114 | 0.990553456 | Up |
| ARMC8 | -1.363523112 | 0.023047531 | 0.990553456 | Down |
| XLOC_011204 | -1.729772269 | 0.023129916 | 0.990553456 | Down |
| CCDC155 | 1.01766748 | 0.023176989 | 0.990553456 | Up |
| XLOC_l2_003380 | 1.056731561 | 0.023191149 | 0.990553456 | Up |
| UGDH | -1.317300307 | 0.02327894 | 0.990553456 | Down |
| XLOC_l2_011145 | 1.022651133 | 0.023485015 | 0.990553456 | Up |
| C11orf82 | 1.112166836 | 0.023490678 | 0.990553456 | Up |
| XLOC_003829 | 1.279451953 | 0.023497221 | 0.990553456 | Up |
| XLOC_l2_001760 | 1.123356221 | 0.023557404 | 0.990553456 | Up |
| XLOC_005629 | 1.082568327 | 0.023716002 | 0.990553456 | Up |
| SIGLEC15 | -1.278338761 | 0.023723951 | 0.990553456 | Down |
| MFAP3L | -1.649470722 | 0.023847211 | 0.990553456 | Down |
| XLOC_013699 | 1.057350999 | 0.024008383 | 0.990553456 | Up |
| XLOC_002969 | 1.592276364 | 0.02408802 | 0.990553456 | Up |
| XLOC_l2_006574 | 1.078427158 | 0.02425506 | 0.990553456 | Up |
| CADM3 | -2.273007864 | 0.024317236 | 0.990553456 | Down |
| RPL23P8 | -1.290904092 | 0.024337977 | 0.990553456 | Down |
| XLOC_001944 | 1.104236139 | 0.024368964 | 0.990553456 | Up |
| XLOC_010774 | 1.465669648 | 0.024391076 | 0.990553456 | Up |
| ZNF528 | -1.000748054 | 0.024425491 | 0.990553456 | Down |
| XLOC_008401 | 1.684860734 | 0.024463152 | 0.990553456 | Up |
| XLOC_007726 | 1.25732846 | 0.024482233 | 0.990553456 | Up |
| XLOC_004065 | 1.355745267 | 0.024518144 | 0.990553456 | Up |
| XLOC_l2_003475 | 1.243824788 | 0.024577239 | 0.990553456 | Up |
| XLOC_004227 | -1.155134776 | 0.024597335 | 0.990553456 | Down |
| XLOC_010432 | -1.098989584 | 0.024643003 | 0.990553456 | Down |
| SRD5A2 | -1.002766665 | 0.0246496 | 0.990553456 | Down |
| XLOC_008552 | 1.055146095 | 0.024668451 | 0.990553456 | Up |
| CAMK2G | -1.021502197 | 0.024704112 | 0.990553456 | Down |
| XLOC_006863 | -1.252131237 | 0.024770847 | 0.990553456 | Down |
| RFPL4B | 1.413540411 | 0.024875442 | 0.990553456 | Up |
| SLC45A2 | 1.111396872 | 0.024876817 | 0.990553456 | Up |
| HAS2 | 1.444888006 | 0.025001997 | 0.990553456 | Up |
| OR1J2 | 1.034929635 | 0.025025251 | 0.990553456 | Up |
| XLOC_005575 | -1.87628129 | 0.025081415 | 0.990553456 | Down |
| LPAR1 | -1.232761312 | 0.025097352 | 0.990553456 | Down |
| PSG8 | 1.138037138 | 0.025106089 | 0.990553456 | Up |
| XLOC_011401 | -1.593031001 | 0.025167369 | 0.990553456 | Down |
| SRG7 | 1.222311995 | 0.025218591 | 0.990553456 | Up |
| CYP4F11 | -1.314924823 | 0.025240765 | 0.990553456 | Down |
| XLOC_004459 | 1.123710585 | 0.025339809 | 0.990553456 | Up |
| CDCA2 | 1.248747261 | 0.025421404 | 0.990553456 | Up |
| BMPR1A | -1.031082221 | 0.025484304 | 0.990553456 | Down |
| XLOC_013218 | 1.001876147 | 0.025638739 | 0.990553456 | Up |
| VPS36 | -1.08202377 | 0.025739732 | 0.990553456 | Down |
| LRRC66 | 1.004115604 | 0.025744145 | 0.990553456 | Up |
| CNTN5 | 1.013892867 | 0.025791504 | 0.990553456 | Up |
| DEFB125 | 1.895441679 | 0.025792304 | 0.990553456 | Up |
| XLOC_004752 | 1.127928297 | 0.025883561 | 0.990553456 | Up |
| RPPH1 | -1.322496744 | 0.025975982 | 0.990553456 | Down |
| HHIP | -1.705609535 | 0.026037291 | 0.990553456 | Down |
| XLOC_000545 | 1.11526589 | 0.026081012 | 0.990553456 | Up |
| C17orf6 | 1.084842422 | 0.026201048 | 0.990553456 | Up |
| XLOC_012241 | 1.012570651 | 0.026224948 | 0.990553456 | Up |
| LHX8 | -1.594791495 | 0.026431058 | 0.990553456 | Down |
| CLSPN | 1.155316492 | 0.026485304 | 0.990553456 | Up |
| FAM182A | 1.344783048 | 0.026567034 | 0.990553456 | Up |
| XLOC_002678 | 1.310516816 | 0.026650242 | 0.990553456 | Up |
| TSPAN8 | -1.239745312 | 0.026670963 | 0.990553456 | Down |
| XLOC_009531 | 1.130120527 | 0.026750713 | 0.990553456 | Up |
| XLOC_003521 | 1.183066515 | 0.026828559 | 0.990553456 | Up |
| XLOC_008481 | 1.445203371 | 0.02693529 | 0.990553456 | Up |
| CHPF | 1.060998736 | 0.026969802 | 0.990553456 | Up |
| ZMYM2 | -1.011410708 | 0.026973086 | 0.990553456 | Down |
| XLOC_001815 | 1.132427188 | 0.0270964 | 0.990553456 | Up |
| TXNIP | -1.085019043 | 0.027292276 | 0.990553456 | Down |
| DLGAP2 | 1.317153754 | 0.027335855 | 0.990553456 | Up |
| C20orf132 | -1.310605049 | 0.027352517 | 0.990553456 | Down |
| XLOC_008367 | 1.338510637 | 0.027432928 | 0.990553456 | Up |
| XLOC_000980 | 1.125653902 | 0.027593937 | 0.990553456 | Up |
| XLOC_009312 | 1.04617666 | 0.027596418 | 0.990553456 | Up |
| TTTY10 | 1.710148045 | 0.027605526 | 0.990553456 | Up |
| XLOC_008637 | 1.340869646 | 0.027655564 | 0.990553456 | Up |
| C7orf62 | 1.39395736 | 0.027681744 | 0.990553456 | Up |
| OR5I1 | 1.625908251 | 0.027764238 | 0.990553456 | Up |
| SKA3 | 1.707457473 | 0.027767721 | 0.990553456 | Up |
| UVRAG | -1.018262254 | 0.027797707 | 0.990553456 | Down |
| KLRF2 | 1.379770129 | 0.027842039 | 0.990553456 | Up |
| NEIL3 | 1.163077186 | 0.027915971 | 0.990553456 | Up |
| XLOC_001064 | -1.124314647 | 0.027923606 | 0.990553456 | Down |
| XLOC_008512 | -1.779615298 | 0.028150839 | 0.990553456 | Down |
| XLOC_010881 | -1.641104641 | 0.028192589 | 0.990553456 | Down |
| KIF20A | 1.11646956 | 0.028210383 | 0.990553456 | Up |
| XLOC_005224 | -1.061284595 | 0.028306878 | 0.990553456 | Down |
| HOMER2 | 1.100719185 | 0.028638528 | 0.990553456 | Up |
| BEND5 | -1.195427901 | 0.028718819 | 0.990553456 | Down |
| XLOC_l2_015205 | -1.033655997 | 0.02879373 | 0.990553456 | Down |
| XLOC_003803 | 1.019327144 | 0.028861308 | 0.990553456 | Up |
| ADAMTS9-AS1 | 1.672519782 | 0.028934587 | 0.990553456 | Up |
| L3MBTL3 | -1.093299089 | 0.02897009 | 0.990553456 | Down |
| XLOC_003093 | 1.551956564 | 0.029110334 | 0.990553456 | Up |
| XLOC_l2_014757 | -1.18448966 | 0.029203969 | 0.990553456 | Down |
| FLJ13197 | -1.134844109 | 0.029225142 | 0.990553456 | Down |
| KCNA10 | 1.154279939 | 0.029263625 | 0.990553456 | Up |
| CCDC73 | -1.078492748 | 0.029330075 | 0.990553456 | Down |
| EEF1DP3 | -1.079960746 | 0.029360776 | 0.990553456 | Down |
| IFNW1 | -1.109271315 | 0.029453936 | 0.990553456 | Down |
| TMPRSS11A | 1.168105107 | 0.029457968 | 0.990553456 | Up |
| GDF15 | -1.471428099 | 0.029461981 | 0.990553456 | Down |
| XLOC_l2_013633 | 1.181241981 | 0.029483227 | 0.990553456 | Up |
| XLOC_004951 | 1.341254472 | 0.029662066 | 0.990553456 | Up |
| PLEKHN1 | -1.332099759 | 0.02966669 | 0.990553456 | Down |
| LINC00313 | 1.411670077 | 0.029727633 | 0.990553456 | Up |
| ARHGAP11A | 1.013472019 | 0.029856287 | 0.990553456 | Up |
| SMAD6 | -1.111995761 | 0.02992093 | 0.990553456 | Down |
| MLNR | 1.130294915 | 0.029945472 | 0.990553456 | Up |
| XLOC_006177 | 1.202836144 | 0.030241752 | 0.990553456 | Up |
| XLOC_004569 | 1.0294532 | 0.03048729 | 0.990553456 | Up |
| XLOC_013480 | 1.068426395 | 0.030670487 | 0.990553456 | Up |
| BREA2 | -1.184850834 | 0.030742845 | 0.990553456 | Down |
| ACOX1 | -1.175943185 | 0.030743076 | 0.990553456 | Down |
| XLOC_013921 | 1.082838099 | 0.030823097 | 0.990553456 | Up |
| XLOC_002732 | 1.010487512 | 0.030949254 | 0.990553456 | Up |
| XLOC_013309 | -1.065388062 | 0.030989378 | 0.990553456 | Down |
| SEMA4G | 1.050454948 | 0.03108354 | 0.990553456 | Up |
| XLOC_005442 | 1.086021076 | 0.031091191 | 0.990553456 | Up |
| MMP12 | 1.880011713 | 0.03117176 | 0.990553456 | Up |
| TIGD3 | 1.108927113 | 0.031297914 | 0.990553456 | Up |
| SNORA1 | -1.015319806 | 0.031454901 | 0.990553456 | Down |
| IRX4 | 1.248682706 | 0.031493473 | 0.990553456 | Up |
| LSAMP-AS3 | -1.087392903 | 0.031562261 | 0.990553456 | Down |
| SFRP2 | 1.891714424 | 0.031649428 | 0.990553456 | Up |
| XLOC_011980 | -1.299667517 | 0.031780327 | 0.990553456 | Down |
| HHAT | -1.07529586 | 0.03182537 | 0.990553456 | Down |
| XLOC_006229 | -1.02989992 | 0.032126062 | 0.990553456 | Down |
| C1orf129 | 1.041317363 | 0.032194375 | 0.990553456 | Up |
| FLJ44511 | -1.070292332 | 0.03222451 | 0.990553456 | Down |
| XLOC_008986 | 1.173021105 | 0.03232638 | 0.990553456 | Up |
| XLOC_012296 | 1.047657282 | 0.032403413 | 0.990553456 | Up |
| BTLA | 1.172805474 | 0.032638741 | 0.990553456 | Up |
| XLOC_006811 | 1.042104257 | 0.032639066 | 0.990553456 | Up |
| XLOC_014372 | 1.250165342 | 0.032655456 | 0.990553456 | Up |
| CYP11B2 | 1.081874855 | 0.032736325 | 0.990553456 | Up |
| C18orf26 | -1.187070502 | 0.032790535 | 0.990553456 | Down |
| XLOC_006338 | -1.020122015 | 0.032802409 | 0.990553456 | Down |
| XLOC_l2_005517 | -1.141231983 | 0.033034062 | 0.990553456 | Down |
| RHOJ | 1.236722169 | 0.033079018 | 0.990553456 | Up |
| OR52K1 | 1.081422507 | 0.033309139 | 0.990553456 | Up |
| L1TD1 | 1.032675327 | 0.033334084 | 0.990553456 | Up |
| KRTAP25-1 | 1.162738094 | 0.033345387 | 0.990553456 | Up |
| XLOC_l2_013282 | 1.223608316 | 0.033404323 | 0.990553456 | Up |
| XLOC_l2_015239 | -1.742271682 | 0.033405237 | 0.990553456 | Down |
| RGS21 | 1.159304184 | 0.033568291 | 0.990553456 | Up |
| CTHRC1 | 1.428602336 | 0.033638524 | 0.990553456 | Up |
| ZNF204P | -1.114432239 | 0.033684241 | 0.990553456 | Down |
| XLOC_007012 | 1.170589438 | 0.034145829 | 0.990553456 | Up |
| LY6K | -1.340333364 | 0.034603471 | 0.990553456 | Down |
| LECT2 | 1.22016094 | 0.034688198 | 0.990553456 | Up |
| CST1 | 1.176783882 | 0.035022523 | 0.990553456 | Up |
| XLOC_006512 | -1.043733138 | 0.035115454 | 0.990553456 | Down |
| CDC42 | -1.057450279 | 0.035211008 | 0.990553456 | Down |
| C12orf77 | -1.044639491 | 0.035345585 | 0.990553456 | Down |
| KRTAP10-5 | 1.019652523 | 0.035372877 | 0.990553456 | Up |
| HSD17B7 | -1.039469254 | 0.035446134 | 0.990553456 | Down |
| PDP1 | -1.108605551 | 0.03552939 | 0.990553456 | Down |
| XLOC_002342 | 1.579189596 | 0.035551454 | 0.990553456 | Up |
| FRMD5 | 1.062119106 | 0.03568443 | 0.990553456 | Up |
| FOXP1-IT1 | -1.260983099 | 0.035921374 | 0.990553456 | Down |
| DNASE2B | 1.622108537 | 0.036165683 | 0.990553456 | Up |
| XLOC_001394 | 1.075164163 | 0.036254948 | 0.990553456 | Up |
| XLOC_l2_008783 | 1.501977259 | 0.036695119 | 0.990553456 | Up |
| XLOC_002252 | 1.015060651 | 0.036909338 | 0.990553456 | Up |
| SLC5A9 | 1.167278913 | 0.036913548 | 0.990553456 | Up |
| MIER1 | -1.145593878 | 0.036938586 | 0.990553456 | Down |
| TTTY9A | 1.038702268 | 0.037408961 | 0.990553456 | Up |
| TMPRSS11BNL | -1.126669541 | 0.037480288 | 0.990553456 | Down |
| DNASE1L3 | -1.085724918 | 0.037485861 | 0.990553456 | Down |
| SULT2B1 | 1.091304576 | 0.037533686 | 0.990553456 | Up |
| SNAPC4 | -1.198963255 | 0.037599441 | 0.990553456 | Down |
| CDC45 | 1.599630332 | 0.037700724 | 0.990553456 | Up |
| XLOC_012866 | 1.076334773 | 0.037729429 | 0.990553456 | Up |
| XLOC_007963 | 1.048441629 | 0.037786481 | 0.990553456 | Up |
| SHCBP1 | 1.096171006 | 0.037856813 | 0.990553456 | Up |
| MNX1 | 1.072591831 | 0.038093023 | 0.990553456 | Up |
| XLOC_010633 | 1.284692618 | 0.038157388 | 0.990553456 | Up |
| SORD | -1.12510218 | 0.038185371 | 0.990553456 | Down |
| XLOC_008374 | 1.18970194 | 0.038248173 | 0.990553456 | Up |
| XLOC_002433 | -1.099291064 | 0.038365933 | 0.990553456 | Down |
| E2F2 | 1.482002954 | 0.038377497 | 0.990553456 | Up |
| XLOC_008510 | 1.090604796 | 0.038450889 | 0.990553456 | Up |
| TMEM144 | -1.151340068 | 0.038488518 | 0.990553456 | Down |
| LINS | 1.284578764 | 0.038591647 | 0.990553456 | Up |
| CEP44 | 1.266056695 | 0.038699791 | 0.990553456 | Up |
| XLOC_011639 | 1.042407339 | 0.038726772 | 0.990553456 | Up |
| EMR4P | 1.123916082 | 0.038821283 | 0.990553456 | Up |
| XLOC_011858 | 1.797664238 | 0.03891361 | 0.990553456 | Up |
| COL25A1 | 1.251258244 | 0.039034231 | 0.990553456 | Up |
| CXorf57 | -1.517503597 | 0.039139783 | 0.990553456 | Down |
| RNASE8 | 1.086915679 | 0.039221135 | 0.990553456 | Up |
| Q8WY88 | -1.15380649 | 0.039255405 | 0.990553456 | Down |
| C12orf36 | 1.788468123 | 0.039299866 | 0.990553456 | Up |
| IRX5 | 1.642037776 | 0.039310972 | 0.990553456 | Up |
| TMCO5A | 1.129865484 | 0.039368262 | 0.990553456 | Up |
| XLOC_l2_015068 | 1.000672337 | 0.039498469 | 0.990553456 | Up |
| FAM59A | -1.114681997 | 0.03952207 | 0.990553456 | Down |
| PTPRD | -2.065829433 | 0.039583058 | 0.990553456 | Down |
| SNORA30 | 1.153783411 | 0.039629188 | 0.990553456 | Up |
| XLOC_013804 | 1.095962162 | 0.039767365 | 0.990553456 | Up |
| PLXNB3 | -1.805823628 | 0.039906599 | 0.990553456 | Down |
| LHX2 | 1.226243743 | 0.040191761 | 0.990553456 | Up |
| XLOC_010896 | 1.01224703 | 0.040249692 | 0.990553456 | Up |
| OR2W5 | 1.160195241 | 0.040325607 | 0.990553456 | Up |
| UBQLN1 | -1.023683895 | 0.040555298 | 0.990553456 | Down |
| XLOC_l2_011694 | 1.048100404 | 0.040559322 | 0.990553456 | Up |
| PDHA2 | 1.325501039 | 0.040575271 | 0.990553456 | Up |
| IRGM | 1.144629835 | 0.040598011 | 0.990553456 | Up |
| ANKRD6 | -1.185806572 | 0.040618608 | 0.990553456 | Down |
| SRY | 1.264221152 | 0.040791656 | 0.990553456 | Up |
| XLOC_011971 | 1.102230263 | 0.040819465 | 0.990553456 | Up |
| KRTAP1-5 | 1.141525084 | 0.040906631 | 0.990553456 | Up |
| XLOC_l2_010947 | 1.077140145 | 0.040998333 | 0.990553456 | Up |
| OR10A5 | 1.405539026 | 0.041091074 | 0.990553456 | Up |
| XLOC_010728 | 1.663530663 | 0.04128875 | 0.990553456 | Up |
| XLOC_010927 | 1.013531429 | 0.041351038 | 0.990553456 | Up |
| SNAP25 | 1.044453641 | 0.041599066 | 0.990553456 | Up |
| TMC5 | 1.895782216 | 0.04164179 | 0.990553456 | Up |
| XLOC_006666 | 1.011010107 | 0.041669472 | 0.990553456 | Up |
| XLOC_009820 | 1.002908171 | 0.041698822 | 0.990553456 | Up |
| MAPK4 | -1.076504005 | 0.041800253 | 0.990553456 | Down |
| PLIN5 | -1.279789561 | 0.041837135 | 0.990553456 | Down |
| ZXDB | -1.009610971 | 0.041946509 | 0.990553456 | Down |
| TRPA1 | 1.032383129 | 0.041977475 | 0.990553456 | Up |
| MS4A14 | 1.190073529 | 0.042007132 | 0.990553456 | Up |
| TMEM30B | -1.435841149 | 0.042034243 | 0.990553456 | Down |
| TRIM49L2 | 1.612135879 | 0.042163142 | 0.990553456 | Up |
| OGN | -1.788449064 | 0.042201655 | 0.990553456 | Down |
| XLOC_011017 | -1.086572366 | 0.042248863 | 0.990553456 | Down |
| SMCR6 | -1.015241565 | 0.042281539 | 0.990553456 | Down |
| TTYH2 | 1.074337647 | 0.042285419 | 0.990553456 | Up |
| CDY2A | 1.158793179 | 0.042331828 | 0.990553456 | Up |
| XLOC_012819 | 1.26425309 | 0.042515035 | 0.990553456 | Up |
| MS4A1 | 1.538040366 | 0.042662097 | 0.990553456 | Up |
| IFNA21 | 1.317935327 | 0.042733004 | 0.990553456 | Up |
| XLOC_000564 | 1.421476514 | 0.042738397 | 0.990553456 | Up |
| GRAMD2 | 1.260020935 | 0.042831367 | 0.990553456 | Up |
| KIT | -1.237535542 | 0.042862103 | 0.990553456 | Down |
| DTX1 | 1.011329965 | 0.04290241 | 0.990553456 | Up |
| SNORD60 | -1.210912329 | 0.042963706 | 0.990553456 | Down |
| RNF180 | -1.206453501 | 0.04307632 | 0.990553456 | Down |
| XLOC_001855 | 1.06037347 | 0.043410605 | 0.990553456 | Up |
| XLOC_013754 | 1.26971669 | 0.043593222 | 0.990553456 | Up |
| UBD | 1.819885435 | 0.043817465 | 0.990553456 | Up |
| PLEKHA6 | -1.01068577 | 0.043856141 | 0.990553456 | Down |
| XLOC_012623 | 1.129695602 | 0.043921931 | 0.990553456 | Up |
| XLOC_008975 | -1.2106992 | 0.043946745 | 0.990553456 | Down |
| XLOC_l2_014785 | -1.616831216 | 0.044121236 | 0.990553456 | Down |
| ABCD2 | -1.226492842 | 0.044288727 | 0.990553456 | Down |
| XLOC_003136 | -2.048549442 | 0.044351751 | 0.990553456 | Down |
| TMEM154 | -1.119690266 | 0.044488499 | 0.990553456 | Down |
| ZSCAN20 | -1.008002436 | 0.04459697 | 0.990553456 | Down |
| XLOC_l2_007898 | 1.314326174 | 0.044605865 | 0.990553456 | Up |
| KCNAB2 | 1.124896064 | 0.044742561 | 0.990553456 | Up |
| XLOC_011369 | 1.008833107 | 0.04484137 | 0.990553456 | Up |
| PLIN1 | -1.310742235 | 0.044842231 | 0.990553456 | Down |
| BPI | 1.037019169 | 0.045108075 | 0.990553456 | Up |
| EPHA5 | 1.095303748 | 0.045131199 | 0.990553456 | Up |
| RIPPLY2 | -1.247683004 | 0.04524697 | 0.990553456 | Down |
| XLOC_l2_010926 | 1.181962224 | 0.045416205 | 0.990553456 | Up |
| OR13C2 | -1.137259898 | 0.045430776 | 0.990553456 | Down |
| NR2F6 | -1.0519282 | 0.045433317 | 0.990553456 | Down |
| XLOC_000914 | 1.265584167 | 0.045520278 | 0.990553456 | Up |
| TET2 | -1.072718846 | 0.045639996 | 0.990553456 | Down |
| XLOC_l2_003881 | -1.008957343 | 0.04565183 | 0.990553456 | Down |
| SULT2A1 | -1.550704729 | 0.045707387 | 0.990553456 | Down |
| CCDC129 | -1.192600661 | 0.045772367 | 0.990553456 | Down |
| TAF1L | 1.136441045 | 0.045847813 | 0.990553456 | Up |
| RUNX1 | -1.304416886 | 0.045996912 | 0.990553456 | Down |
| XLOC_010634 | 1.042860426 | 0.046013939 | 0.990553456 | Up |
| XLOC_010677 | 1.070960442 | 0.046097752 | 0.990553456 | Up |
| BCHE | -1.88722667 | 0.046484593 | 0.990553456 | Down |
| SLAMF1 | 1.33234479 | 0.046536048 | 0.990553456 | Up |
| MC4R | 1.064279554 | 0.046584101 | 0.990553456 | Up |
| SPRR2F | 1.19534222 | 0.046593809 | 0.990553456 | Up |
| XLOC_012034 | 1.045798939 | 0.046596688 | 0.990553456 | Up |
| XLOC_007419 | 1.039510216 | 0.046825094 | 0.990553456 | Up |
| XLOC_000105 | 1.694506409 | 0.047072451 | 0.990553456 | Up |
| XLOC_009944 | 1.00920012 | 0.047072595 | 0.990553456 | Up |
| CYP4F2 | -1.327605065 | 0.047076118 | 0.990553456 | Down |
| CDT1 | 1.137097001 | 0.047116039 | 0.990553456 | Up |
| SGOL2 | 1.086302223 | 0.047289959 | 0.990553456 | Up |
| CCL8 | 1.615375956 | 0.047618701 | 0.990553456 | Up |
| TTTY13 | 1.513443337 | 0.047644674 | 0.990553456 | Up |
| DLEC1 | -1.085254425 | 0.047746995 | 0.990553456 | Down |
| XLOC_007540 | 1.530862119 | 0.0477622 | 0.990553456 | Up |
| C12orf33 | 1.080637769 | 0.047762286 | 0.990553456 | Up |
| XLOC_009365 | 1.037654564 | 0.047766082 | 0.990553456 | Up |
| KRT222 | 1.268330014 | 0.047882645 | 0.990553456 | Up |
| PFN2 | -1.157994498 | 0.048127946 | 0.990553456 | Down |
| FAM81B | 1.052471111 | 0.048288741 | 0.990553456 | Up |
| XLOC_004374 | 1.052543487 | 0.048428646 | 0.990553456 | Up |
| XLOC_010378 | -1.06807633 | 0.048621217 | 0.990553456 | Down |
| KIF16B | 1.060284136 | 0.04862774 | 0.990553456 | Up |
| XLOC_004529 | -1.142000694 | 0.048634551 | 0.990553456 | Down |
| XLOC_001734 | 1.066528792 | 0.048786903 | 0.990553456 | Up |
| FAM190A | -1.012045454 | 0.048864695 | 0.990553456 | Down |
| tAKR | 1.257975389 | 0.048904368 | 0.990553456 | Up |
| SCN3B | -1.051152859 | 0.048921693 | 0.990553456 | Down |
| PARVG | 1.009491803 | 0.048941562 | 0.990553456 | Up |
| PTPLAD1 | -1.02130968 | 0.048957082 | 0.990553456 | Down |
| BMP2 | -1.005175691 | 0.048962529 | 0.990553456 | Down |
| OR2L8 | 1.649810897 | 0.049110753 | 0.990553456 | Up |
| XLOC_l2_010843 | 1.289430992 | 0.049240412 | 0.990553456 | Up |
| CPB1 | 1.140419038 | 0.049302417 | 0.990553456 | Up |
| CEACAM6 | 1.028256115 | 0.049584121 | 0.990553456 | Up |
| SLC12A2 | -1.033153006 | 0.049592983 | 0.990553456 | Down |
| ZNF514 | -1.007547748 | 0.049667873 | 0.990553456 | Down |
| XLOC_007278 | 1.604632557 | 0.049766002 | 0.990553456 | Up |
| LRRIQ1 | 1.206000678 | 0.049842058 | 0.990553456 | Up |
| XLOC_000659 | -1.076292883 | 0.049875712 | 0.990553456 | Down |
