# Supplementary material for: CCL7 and olfactory transduction pathway activation play an important role in the formation of CaOx and CaP kidney stones
Source: Front Genet. 2024 Jan 3;14:1267545. doi: 10.3389/fgene.2023.1267545 (PMC10791818; doi:10.3389/fgene.2023.1267545)
Supplement: Supplementary file 4 [file Table5.docx]

Supplement Table5. Gene names of DEGs

| Both UP | Both Down |
| --- | --- |
| ABCC6P1 | ABCD2 |
| ANKRD34A | ACVR2B |
| C12orf36 | AHNAK2 |
| C17orf6 | BMPR1A |
| C1orf49 | C10orf108 |
| C2orf62 | C20orf194 |
| C7orf62 | C5orf25 |
| CA5A | C7orf41 |
| CBR1 | C7orf55 |
| CCDC155 | CAMK2G |
| CCL7 | CCDC14 |
| CD80 | CEP78 |
| CDH8 | CFL2 |
| CDKN3 | CXCR7 |
| CST1 | DNASE1L3 |
| DEC1 | DYNC1LI2 |
| ELAVL4 | ESCO1 |
| EMR4P | FLJ13197 |
| FAM170A | GATAD2B |
| FAM182A | HHIP |
| FAM83A | HOTAIRM1 |
| FLJ41130 | KIT |
| FMO1 | LEAP2 |
| GK2 | LHX8 |
| GRM8 | LPAR1 |
| HES2 | LY6K |
| HOXB1 | MALAT1 |
| HSFY2 | MFAP3L |
| IDI2 | MGC24103 |
| IRX4 | NEAT1 |
| KCNA10 | NEBL |
| KLHDC8A | NR2F6 |
| KRTAP25-1 | OGN |
| KRTAP4-2 | PI15 |
| LHX2 | PIGZ |
| LTA | PLEKHA6 |
| MC4R | PPP1R3B |
| MMP10 | Q8WY88 |
| MOV10L1 | RNASEL |
| NCR2 | RNF180 |
| OR10A5 | RPL23P8 |
| OR10K1 | RPPH1 |
| OR11H12 | SDHAP1 |
| OR1L3 | SMAD6 |
| OR2L8 | SNAPC4 |
| OR2W5 | SPIRE1 |
| OR4K17 | TET2 |
| OR52E2 | THSD4 |
| OR5I1 | TMEM30B |
| ORC1 | TOR1AIP1 |
| PATE2 | TXNIP |
| PAX5 | UBQLN1 |
| PDHA2 | UVRAG |
| PLSCR5 | VPS36 |
| PPAPDC3 | WDFY3 |
| PSG2 | XLOC_002433 |
| PSG8 | XLOC_002577 |
| Q68PJ0 | XLOC_013274 |
| RGSL1 | XLOC_l2_005517 |
| RNASE8 | XLOC_l2_014785 |
| SKA3 | ZMYM2 |
| SPEM1 | ZNF204P |
| SPP2 | ZNF765 |
| SRG7 | ZNF91 |
| SRY |  |
| tAKR |  |
| TMEM155 |  |
| TMEM63C |  |
| TMPRSS6 |  |
| TRIM49L2 |  |
| TTTY13 |  |
| XLOC_000564 |  |
| XLOC_000832 |  |
| XLOC_000877 |  |
| XLOC_001607 |  |
| XLOC_001734 |  |
| XLOC_001787 |  |
| XLOC_001815 |  |
| XLOC_001877 |  |
| XLOC_001944 |  |
| XLOC_002342 |  |
| XLOC_002524 |  |
| XLOC_002732 |  |
| XLOC_002882 |  |
| XLOC_002887 |  |
| XLOC_002906 |  |
| XLOC_002924 |  |
| XLOC_002969 |  |
| XLOC_003093 |  |
| XLOC_003166 |  |
| XLOC_003521 |  |
| XLOC_003548 |  |
| XLOC_004350 |  |
| XLOC_004374 |  |
| XLOC_004914 |  |
| XLOC_004951 |  |
| XLOC_005210 |  |
| XLOC_005306 |  |
| XLOC_005442 |  |
| XLOC_005910 |  |
| XLOC_005918 |  |
| XLOC_006226 |  |
| XLOC_006316 |  |
| XLOC_006383 |  |
| XLOC_006520 |  |
| XLOC_006652 |  |
| XLOC_006929 |  |
| XLOC_007268 |  |
| XLOC_007278 |  |
| XLOC_007496 |  |
| XLOC_007540 |  |
| XLOC_007643 |  |
| XLOC_007681 |  |
| XLOC_007753 |  |
| XLOC_008222 |  |
| XLOC_008351 |  |
| XLOC_008401 |  |
| XLOC_008481 |  |
| XLOC_008510 |  |
| XLOC_008552 |  |
| XLOC_009250 |  |
| XLOC_009312 |  |
| XLOC_010248 |  |
| XLOC_010674 |  |
| XLOC_010840 |  |
| XLOC_011252 |  |
| XLOC_011316 |  |
| XLOC_011468 |  |
| XLOC_011706 |  |
| XLOC_011807 |  |
| XLOC_011818 |  |
| XLOC_011951 |  |
| XLOC_011971 |  |
| XLOC_012525 |  |
| XLOC_012623 |  |
| XLOC_012819 |  |
| XLOC_013285 |  |
| XLOC_013687 |  |
| XLOC_013699 |  |
| XLOC_013754 |  |
| XLOC_013804 |  |
| XLOC_013812 |  |
| XLOC_013862 |  |
| XLOC_014372 |  |
| XLOC_l2_001037 |  |
| XLOC_l2_001954 |  |
| XLOC_l2_003475 |  |
| XLOC_l2_005187 |  |
| XLOC_l2_005759 |  |
| XLOC_l2_007907 |  |
| XLOC_l2_010843 |  |
| XLOC_l2_010926 |  |
| XLOC_l2_011694 |  |
| XLOC_l2_015143 |  |
| XLOC_l2_015661 |  |
