# Supplementary material for: CCL7 and olfactory transduction pathway activation play an important role in the formation of CaOx and CaP kidney stones
Source: Front Genet. 2024 Jan 3;14:1267545. doi: 10.3389/fgene.2023.1267545 (PMC10791818; doi:10.3389/fgene.2023.1267545)
Supplement: Supplementary file 5 [file Table3.docx]

Supplement Table 3. GO enrichment analyses

| ONTOLOGY | Description | pvalue | p.adjust | geneID | Count |
| --- | --- | --- | --- | --- | --- |
| BP | detection of chemical stimulus in sensory perception | 0.00034 | 0.25845 | CST1/OR10A5/OR10K1/OR11H12/OR1L3/OR2L8/OR4K17/OR52E2/OR5I1 | 9 |
| BP | detection of chemical stimulus in sensory perception of smell | 0.00083 | 0.25845 | OR10A5/OR10K1/OR11H12/OR1L3/OR2L8/OR4K17/OR52E2/OR5I1 | 8 |
| BP | mitral valve morphogenesis | 0.00091 | 0.25845 | BMPR1A/SMAD6 | 2 |
| BP | dorsal/ventral pattern formation | 0.00093 | 0.25845 | LHX2/BMPR1A/HHIP/SMAD6 | 4 |
| BP | mitral valve development | 0.00112 | 0.25845 | BMPR1A/SMAD6 | 2 |
| BP | sensory perception of smell | 0.00120 | 0.25845 | OR10A5/OR10K1/OR11H12/OR1L3/OR2L8/OR4K17/OR52E2/OR5I1 | 8 |
| CC | autophagosome | 0.0163 | 0.6229 | UBQLN1/UVRAG/WDFY3 | 3 |
| CC | autophagosome membrane | 0.0242 | 0.6229 | UVRAG/WDFY3 | 2 |
| CC | chromosomal region | 0.0342 | 0.6229 | ORC1/SKA3/DYNC1LI2/GATAD2B/UVRAG | 5 |
| CC | inclusion body | 0.0484 | 0.6229 | UBQLN1/WDFY3 | 2 |
| CC | outer kinetochore | 0.0541 | 0.6229 | SKA3 | 1 |
| CC | microfibril | 0.0585 | 0.6229 | THSD4 | 1 |
| MF | olfactory receptor activity | 0.000877447 | 0.2140 | OR10A5/OR10K1/OR11H12/OR1L3/OR2L8/OR4K17/OR52E2/OR5I1 | 8 |
| MF | endonuclease activity | 0.002788337 | 0.2829 | RNASE8/DNASE1L3/RNASEL/RPPH1 | 4 |
| MF | transmembrane receptor protein serine/threonine kinase activity | 0.003494508 | 0.2829 | ACVR2B/BMPR1A | 2 |
| MF | endoribonuclease activity | 0.004638124 | 0.2829 | RNASE8/RNASEL/RPPH1 | 3 |
| MF | transmembrane receptor protein kinase activity | 0.006007875 | 0.2931 | ACVR2B/BMPR1A/KIT | 3 |
| MF | nuclease activity | 0.016303337 | 0.4137 | RNASE8/DNASE1L3/RNASEL/RPPH1 | 4 |
