# Supplementary material for: CCL7 and olfactory transduction pathway activation play an important role in the formation of CaOx and CaP kidney stones
Source: Front Genet. 2024 Jan 3;14:1267545. doi: 10.3389/fgene.2023.1267545 (PMC10791818; doi:10.3389/fgene.2023.1267545)
Supplement: Supplementary file 6 [file DataSheet2.DOCX]

**热图（Heat map）：**

从NCBI GEO 公共数据库下载GSE73680的Series Matrix File 数据文件，注析文件为GPL17077，手工排序对照组和疾病组。

logFoldChange=1

adjustP=0.05

library(limma)

rt=read.table("out.expr.txt",sep="\t",header=T,check.names=F)

rt=as.matrix(rt)

rownames(rt)=rt[,1]

exp=rt[,2:ncol(rt)]

dimnames=list(rownames(exp),colnames(exp))

rt=matrix(as.numeric(as.matrix(exp)),nrow=nrow(exp),dimnames=dimnames)

rt=avereps(rt)

rt=normalizeBetweenArrays(as.matrix(rt))

modType=c(rep("con",33),rep("treat",29))

design <- model.matrix(~0+factor(modType))

colnames(design) <- c("con","treat")

fit <- lmFit(rt,design)

cont.matrix<-makeContrasts(treat-con,levels=design)

fit2 <- contrasts.fit(fit, cont.matrix)

fit2 <- eBayes(fit2)

allDiff=topTable(fit2,adjust='fdr',number=200000)

write.table(allDiff,file="limmaTab.xls",sep="\t",quote=F,row.names = T,col.names = NA)

diffSig <- allDiff[with(allDiff, (abs(logFC)>logFoldChange & P.Value < adjustP )), ]

write.table(diffSig,file="Diff.xls",sep="\t",quote=F,row.names = T,col.names = NA)

diffUp <- allDiff[with(allDiff, (logFC>logFoldChange & P.Value < adjustP )), ]

write.table(diffUp,file="Up.xls",sep="\t",quote=F,row.names = T,col.names = NA)

diffDown <- allDiff[with(allDiff, (logFC<(-logFoldChange) & P.Value < adjustP )), ]

write.table(diffDown,file="Down.xls",sep="\t",quote=F,row.names = T,col.names = NA)

hmExp=rt[rownames(diffSig),]

diffExp=rbind(id=colnames(hmExp),hmExp)

write.table(diffExp,file="heatmap.txt",sep="\t",quote=F,col.names=F)

得到的文件：heatmap.txt、Up.xls、Down.xls用于绘制热图；limmaTab用于绘制火山图；Diff用于绘制韦恩图。

library(limma)

library(pheatmap)

library(gplots)

plotdata=read.table("heatmap.txt",sep="\t",header=T,check.names=F,row.names = 1)

plotdata=plotdata[1:30,] #取前30行

annCol <- data.frame(Tissue = rep(c("Normal","Disease"),c(33,29)),

row.names = colnames(plotdata),

stringsAsFactors = F)

annRow <- data.frame(Direct = rep(c("Up","Down"),c(length(up$X),length(down$X))),

row.names = c(up$X,down$X),

stringsAsFactors = F)

annColors <- list("Tissue"=c("Normal"="blue", "Disease"="red"),"Direct"=c("Up"="yellow",

"Down"="green"))

plotdata <- plotdata[c(up$X,down$X),]

plotdata <- t(scale(t(plotdata)))

plotdata[plotdata > 5] <- 5

plotdata[plotdata < -5] <- -5

pdf(file="heatmap.pdf",width = 8,height = 8)

plotdata=na.omit(plotdata) #去掉NA

pheatmap(plotdata,

scale = "none",

annotation_row=annRow,

annotation_col=annCol,

annotation_colors = annColors,

color = colorRampPalette(c("navy", "white", "palevioletred1"))(20),

fontsize_row=7,

fontsize_col=5,

fontsize=9,

cluster_cols = FALSE,

cluster_rows = T,

show_colnames = F,

show_rownames = T)

dev.off()

**火山图（Volcano map）：**

基于GEO2R或者limma的结果

logFoldChange=1

adjustP=0.05

library(limma)

allDiff=read.table("out.expr.txt",sep="\t",header=T,check.names=F)

tiff(file="vol.tiff",

width = 12,

height =12,

units ="cm",

compression="lzw",

bg="white",

res=600)

xMax=max(abs(allDiff$logFC))

yMax=max(-log10(allDiff$P.Value))

plot(allDiff$logFC, -log10(allDiff$P.Value), xlab="log2FC",ylab="-log10(P.Value)",

main="Volcano",xlim=c(-xMax,xMax),ylim=c(0,yMax),yaxs="i",pch=20, cex=0.8)

diffSub=subset(allDiff, P.Value<adjustP & logFC>logFoldChange)

points(diffSub$logFC, -log10(diffSub$P.Value), pch=20, col="palevioletred1",cex=0.8)

diffSub=subset(allDiff, P.Value<adjustP & logFC<(-logFoldChange))

points(diffSub$logFC, -log10(diffSub$P.Value), pch=20, col="dodgerblue",cex=0.8)

abline(v=0,lty=2,lwd=3)

dev.off()

**韦恩图（Venn diagram）：**

基于GEO2R或limma的结果

library(VennDiagram)

library(dplyr) #管道符号

library(openxlsx) #写入xlsl

sam1 <- "CaOx vs Control.火山图用.table.tsv"

sam2 <- "CaP vs Control.火山图用.table.tsv"

outVenn <- "CaOx-CaP.png"

outXlsx <- "CaOx-CaP.xlsx"

tb.1 <- data.table::fread(sam1) %>% as.data.frame() %>%

filter(Gene.symbol != "") %>%

mutate(Disease = ifelse(logFC > 0, "Up","Down")) %>%

filter(P.Value <= 0.05) %>%

filter(logFC >= 1 | logFC <= -1) %>%

filter(!grepl("^MT-|^LOC",Gene.symbol)) %>%

mutate(Label = paste(Gene.symbol,Disease,sep = "_"))

tb.2 <- data.table::fread(sam2) %>% as.data.frame() %>%

filter(Gene.symbol != "") %>%

mutate(Disease = ifelse(logFC > 0, "Up","Down")) %>%

filter(P.Value <= 0.05) %>%

filter(logFC >= 1 | logFC <= -1) %>%

filter(!grepl("^MT-|^LOC",Gene.symbol)) %>%

mutate(Label = paste(Gene.symbol,Disease,sep = "_"))

同高或者同低的重复基因的只保留一个

tb.1 <- tb.1 %>% distinct(Label,.keep_all = T)

tb.2 <- tb.2 %>% distinct(Label,.keep_all = T)

一个基因既高表达又低表达直接剔除

dup1 <- unique(tb.1$Gene.symbol[duplicated(tb.1$Gene.symbol)])

dup2 <- unique(tb.2$Gene.symbol[duplicated(tb.2$Gene.symbol)])

tb.1 <- tb.1 %>% filter(!Gene.symbol %in% dup1)

tb.2 <- tb.2 %>% filter(!Gene.symbol %in% dup2)

g1up <- tb.1$Gene.symbol[tb.1$Disease == "Up"]

g1down <- tb.1$Gene.symbol[tb.1$Disease == "Down"]

g2up <- tb.2$Gene.symbol[tb.2$Disease == "Up"]

g2down <- tb.2$Gene.symbol[tb.2$Disease == "Down"]

venn.diagram(x= list(G1up = g1up,G1down = g1down,G2up = g2up,G2down = g2down),

filename = outVenn, height = 450, width = 450,resolution =300,

imagetype="png", col ="transparent",

fill =c("cornflowerblue","green","yellow","darkorchid1"),

alpha = 0.5,

cex = 0.45,fontface = "bold",

cat.col =c("darkblue", "darkgreen", "orange","darkorchid4"),

cat.cex = 0.45, cat.dist = 0.07)

写出到excel中

bothUp <- intersect(tb.1$Gene.symbol[tb.1$Disease == "Up"],

tb.2$Gene.symbol[tb.2$Disease == "Up"])

bothDown <- intersect(tb.1$Gene.symbol[tb.1$Disease == "Down"],

tb.2$Gene.symbol[tb.2$Disease == "Down"])

out.data <- list(G1data = tb.1,G2data = tb.2,

BothUP = bothUp,BothDown = bothDown)

write.xlsx(out.data,file = outXlsx)

**GO：**

library(dplyr)

library(tibble)

library(openxlsx)

library(ggplot2)

library(ComplexHeatmap)

library(clusterProfiler)

library(org.Hs.eg.db)

library(AnnotationDbi)

library(enrichplot)

diff_genes= read.xlsx("gene_list.xlsx")

entrzid = bitr(diff_genes$genename,

fromType = "SYMBOL",

toType = "ENTREZID",

OrgDb = "org.Hs.eg.db")

MF <- enrichGO(gene = entrzid$ENTREZID,

OrgDb = org.Hs.eg.db,

pvalueCutoff =0.05,

qvalueCutoff = 0.05,

ont="MF", readable =T)

CC <- enrichGO(gene = entrzid$ENTREZID,

OrgDb = org.Hs.eg.db,

pvalueCutoff =0.05,

qvalueCutoff = 0.05,

ont="CC", readable =T)

BP <- enrichGO(gene = entrzid$ENTREZID,

OrgDb = org.Hs.eg.db,

pvalueCutoff =0.05,

qvalueCutoff = 0.05,

ont="BP", readable =T)

ALL <- enrichGO(gene = entrzid$ENTREZID,

OrgDb = org.Hs.eg.db,

pvalueCutoff =0.05,

qvalueCutoff = 0.05,

ont="ALL", readable =T)

write.table(MF,file="MF.txt", sep="\t", quote=F, row.names = F)

write.table(CC,file="CC.txt", sep="\t", quote=F, row.names = F)

write.table(BP,file="BP.txt", sep="\t", quote=F, row.names = F)

write.table(ALL,file="ALL.txt", sep="\t", quote=F, row.names = F)

pdf(file="eGO_barplot.pdf",width = 8,height = 10)

barplot(eG, x = "GeneRatio", color = "p.adjust", #默认参数（x和color可以根据eG里面的内容更改）

showCategory =10, #只显示前10

split="ONTOLOGY") + #以ONTOLOGY类型分开

facet_grid(ONTOLOGY~., scale='free') #以ONTOLOGY类型分开绘图

dev.off()

因GO数目少，此步没有试过。

本次使用的方法是DAVID在线+R。

**KEGG：**

entrzid = bitr(diff_genes$genename,

fromType = "SYMBOL",

toType = "ENTREZID",

OrgDb = "org.Hs.eg.db")

kegg <- enrichKEGG(

gene = entrzid$ENTREZID, #基因列表文件中的基因名称

keyType = 'kegg',

organism = 'hsa',

pAdjustMethod = 'fdr', #指定 p 值校正方法

pvalueCutoff = 0.5, #指定 p 值阈值

qvalueCutoff = 0.5, #指定 q 值阈值

use_internal_data = F)

barplot(kegg)

**GESA：**

先做差异分析，求出LogFC

library(data.table)

library(clusterProfiler)

library(dplyr)

library(org.Hs.eg.db)

library(ggplot2)

library(enrichplot)

data <- read.table("GSEA1",sep="\t",header=T,check.names=F)

gene.df <- bitr(data$SYMBOL,

fromType = "SYMBOL",

toType = c("ENTREZID"),

OrgDb = org.Hs.eg.db)

data2=merge(data,gene.df,by.y="SYMBOL")

geneList<-data2$logFC

names(geneList)=data2$ENTREZID

geneList=sort(geneList,decreasing = T)

kk <- gseKEGG(geneList, organism = "hsa",pvalueCutoff = 0.2,

nPerm = 1000, minGSSize = 10, maxGSSize = 500,

verbose = TRUE, seed = FALSE, by = "fgsea")

gseaplot2(kk,1,color="red",pvalue_table = T,title="",base_size=10,ES_geom="line") #如果展示的多，1那里可以改，但不是按照P大小排的，故按照下面的代码

kk_d=as.data.frame(kk) #转换成数据框格式

t_index=order(kk_d$enrichmentScore,decreasing = T) #排序

path=rownames(kk[t_index,])[1:4] #选择展示的pathway

gseaplot2(kk,path,

subplots = 1:2, #展示前2个图（共3个）

pvalue_table = T, #显示p值

title = "", #设置title

base_size = 10, #字体大小

#color="red") #线条颜色可选
