# Supplementary material for: CCL7 and olfactory transduction pathway activation play an important role in the formation of CaOx and CaP kidney stones
Source: Front Genet. 2024 Jan 3;14:1267545. doi: 10.3389/fgene.2023.1267545 (PMC10791818; doi:10.3389/fgene.2023.1267545)
Supplement: Supplementary file 7 [file Table4.docx]

| Description | pvalue | p.adjust | geneID | Count |
| --- | --- | --- | --- | --- |
| Olfactory transduction | 0.000620825 | 0.068911571 | OR10A5/OR10K1/OR11H12/OR1L3/OR2L8/OR4K17/OR52E2/OR5I1/CAMK2G | 9 |
| TGF-beta signaling pathway | 0.003010868 | 0.167103148 | ACVR2B/BMPR1A/SMAD6/THSD4 | 4 |
| Type I diabetes mellitus | 0.023891195 | 0.719430013 | CD80/LTA | 2 |
| Phospholipase D signaling pathway | 0.049417962 | 0.719430013 | HOXB1/ACVR2B/BMPR1A | 3 |
| Central carbon metabolism in cancer | 0.058123292 | 0.719430013 | PDHA2/KIT | 2 |
| Cytokine-cytokine receptor interaction | 0.083351491 | 0.719430013 | CCL7/LTA/ACVR2B/BMPR1A | 4 |

Supplement Table 4. Kegg enrichment analyses
